# 1-Butyl-3-methylimidazolium tetrafluoroborate as suitable solvent for BF_3_: the case of alkyne hydration. Chemistry vs electrochemistry

**DOI:** 10.3762/bjoc.19.147

**Published:** 2023-12-28

**Authors:** Marta David, Elisa Galli, Richard C D Brown, Marta Feroci, Fabrizio Vetica, Martina Bortolami

**Affiliations:** 1 Department of Basic and Applied Sciences for Engineering (SBAI), Sapienza University of Rome, via Castro Laurenziano, 7, 00161 Rome, Italy; 2 School of Chemistry, University of Southampton, Southampton SO17 1BJ, UK; 3 Department of Chemistry, Sapienza University of Rome, piazzale Aldo Moro, 5, 00185 Rome, Italy

**Keywords:** alkyne hydration, boron trifluoride, electrochemical synthesis, ionic liquids

## Abstract

In order to replace the expensive metal/ligand catalysts and classic toxic and volatile solvents, commonly used for the hydration of alkynes, the hydration reaction of alkynes was studied in the ionic liquid 1-butyl-3-methylimidazolium tetrafluoroborate (BMIm-BF_4_) adding boron trifluoride diethyl etherate (BF_3_·Et_2_O) as catalyst. Different ionic liquids were used, varying the cation or the anion, in order to identify the best one, in terms of both efficiency and reduced costs. The developed method was efficaciously applied to different alkynes, achieving the desired hydration products with good yields. The results obtained using a conventional approach (i.e., adding BF_3_·Et_2_O) were compared with those achieved using BF_3_ electrogenerated in BMIm-BF_4_, demonstrating the possibility of obtaining the products of alkyne hydration with analogous or improved yields, using less hazardous precursors to generate the reactive species in situ. In particular, for terminal arylalkynes, the electrochemical route proved to be advantageous, yielding preferentially the hydration products vs the aldol condensation products. Importantly, the ability to recycle the ionic liquid in subsequent reactions was successfully demonstrated.

## Introduction

Alkynes are fundamental starting materials towards more complex organic compounds, widely used both in organic chemistry and in electrochemistry as raw materials for the preparation of different molecules of pharmaceutical and industrial interest [1–9]. Among the different organic transformations involving alkynes, their hydration is a well-known and useful reaction in organic chemistry, affording carbonyl compounds based on an atom-economical approach. Indeed, the addition of water to the triple bond of a terminal alkyne leads to the formation of the corresponding methyl ketone or aldehyde, in the case of Markovnikov or *anti*-Markovnikov addition, respectively. On the other hand, the hydration of an internal unsymmetrical alkyne can lead to the formation of the two possible regioisomeric ketones.

The hydration reaction requires a catalytic species, able to polarize the alkyne triple bond to facilitate water attack. Initially, in 1881, Kucherov identified mercury(II) salts in sulfuric acid as efficient promoters of the hydration of alkynes and this catalyst system has found applications in industrial scale synthesis [10]. However, the toxicity and the environmental issues associated with the use of mercury-based compounds have stimulated the search for alternative catalysts and conditions for the hydration of alkynes, in order to identify safer and more sustainable methods [11–13]. In particular, transition-metal catalysts containing Au(I) or (III) [14–24], Ru(II) [25–30], Pd(II) [31–33], Pt(II) [34–35], Fe(III) [36–37], Cu(I) [38–41], Co(III) [42–44], as well as other metals, have been widely studied. In addition, methods involving Brønsted acids, alone or in presence of Lewis acids as co-catalysts, have been developed [45–54]. However, some of these procedures suffer from major drawbacks, such as the toxicity and/or high cost of the metal catalysts, the need to use concentrated Brønsted acids in high excess, long reaction times, and high temperatures. In addition, these reactions have been studied mainly in classical volatile and, in some cases, toxic organic solvents, such as dioxane, tetrahydrofuran, methanol, dichloromethane or 1,2-dichloroethane.

The efficiency of the reported catalysts and of the examined reaction conditions are variable according to the alkynes considered and, nowadays, the identification of new catalysts as well as increasingly mild, economic and sustainable reaction conditions remain fundamental objectives for research in the field of organic chemistry. In recent years, alternative methods have been developed, including the use of different heterogeneous catalysts, to ensure their recovery and reusability after several reaction cycles [55–68], or the use of eco-friendly reaction media [69–72]. Recently, Zhang and co-workers reported an electrochemical procedure for the hydration of arylacetylenes, under mild reaction conditions, without transition metal catalysts, added oxidants, or strong acids involved, using Selectfluor (1-(chloromethyl)-4-fluoro-1,4-diazabicyclo[2.2.2]octane-1,4-diium ditetrafluoroborate) as essential additive [73].

With regard to the reaction medium, the idea of replacing classic organic solvents with alternative solvents could represent an important innovation for alkyne hydration. In particular, ionic liquids (ILs) could represent a valid alternative to conventional organic solvents. ILs are generally liquid salts at or near room temperature, formed by large unsymmetrical organic cations and weakly coordinating or not-coordinating organic or inorganic anions. They have interesting physicochemical properties that differentiate them from the organic solvents commonly used in synthesis [74–77]. Importantly, they have a very low vapour pressure, and therefore do not behave as air pollutants. This also facilitates their recovery and recycling. Furthermore, they generally exhibit low flammability, high thermal and chemical stability, good thermal and electrical conductivity, together with the ability to solubilize organic and inorganic compounds of different polarity [78–81]. Considering the intrinsic ionic nature of ILs, they act as very different chemical medium compared to molecular solvents, having the possibility of stabilizing charged or dipolar intermediates. Therefore, ILs can be used to modulate outcomes for some chemical reactions [82–83].

There are only a few reported examples of the hydration reaction of alkynes carried out in ILs. In one case, a dicationic IL, containing sulfuric acid as catalyst, was used as reaction medium to carry out the hydration of different alkynes under mild conditions (40–60 °C, 0.5–1 h) [84]. In a second case, different Brønsted acid ionic liquids (BAILs) have been used both as medium and as catalysts for the hydration of various alkynes (60 °C, 10–24 h) [85–86]. In these works, the ILs were efficiently reused for subsequent reaction cycles. Another research group reported the use of commercially available 1-butyl-3-methylimidazolium hexafluorophosphate (BMIm-PF_6_) as co-solvent with methanol and water to allow recycling of a phosphine-based Au(I) complex, as an efficient catalytic system for the hydration of terminal alkynes [87]. Moreover, the interesting properties of ILs have also been exploited to synthesize new solid polymeric catalysts for the hydration of alkynes, named poly(ionic liquid)s (PILs), using trifluoroethanol as solvent [88–89].

One of the most studied classes of ILs in organic chemistry are 1,3-disubstitued imidazolium cations, which are cheap, liquid over a wide range of temperatures, and possess good solvating properties [90–91]. Due to their wide electrochemical window, imidazolium ILs are commonly used in organic electrochemistry, simultaneously as solvents and supporting electrolytes [92–94]. In addition, the cathodic reduction (both in batch [95] and in flow [96]) can be exploited for the generation of N-heterocyclic carbenes (NHCs), extensively studied as organocatalysts as well as ligands for transition-metal-promoted synthetic methodologies [97–99]. Under anodic oxidation, the electrogeneration of boron trifluoride (BF_3_) from tetrafluoroborate ILs occurs [100–101]. Moreover, we have recently demonstrated the possibility to efficiently apply the electrogenerated BF_3_ in IL for different classical acid-catalysed reactions [102–103]. Specifically, electrogenerated BF_3_ in 1-butyl-3-methylimidazolium tetrafluoroborate (BMIm-BF_4_) appears as an alternative and less dangerous source of BF_3_ compared to commercially available BF_3_ diethyl etherate (BF_3_·Et_2_O), commonly used in organic synthesis. Indeed, the main advantages of the developed system are: 1) in situ generation of BF_3_, which avoids its storage and handling, 2) the possibility to control the amount of electrogenerated BF_3_ using current by simply starting or stopping the electrolysis, 3) the absence of fuming, most probably due to the ability of the IL to stabilize the Lewis acid, 4) reduced sensitivity to moisture, due to the protective action of the IL, and 5) the possibility of recycling the same sample of IL for subsequent reaction cycles. In addition, with computational studies we demonstrated the greater stability of BF_3_ in BMIm-BF_4_ compared to BF_3_·Et_2_O [103].

Based on the ever increasing need to identify new eco-friendly catalysts and/or reaction media for the hydration of alkynes, and considering our previous works on ILs and electrogeneration of BF_3_, the aim of this work was to explore the hydration of alkynes using ILs as reaction medium and BF_3_ as catalyst. First of all, we investigated the behaviour of diphenylacetylene in BMIm-BF_4_ containing BF_3_·Et_2_O. Then we evaluated the same reaction in different ILs, modifying the cation or the anion. Subsequently, we extended the method to different internal and terminal alkynes. Finally, we studied the reaction in the electrogenerated BF_3_/BMIm-BF_4_ system, comparing the results with those obtained with the chemical route (BF_3_·Et_2_O).

## Results and Discussion

### Optimization of the reaction conditions for hydration of diphenylacetylene in BMIm-BF_4_ with BF_3_·Et_2_O

In the initial investigation, the internal alkyne diphenylacetylene (**1a**) was selected as a model substrate to evaluate alkyne reactivity in the ionic liquid 1-butyl-3-methylimidazolium tetrafluoroborate (BMIm-BF_4_) catalysed by BF_3_·Et_2_O and to optimize the reaction conditions for hydration. All reactions were carried out in sealed vials, in 1 mL BMIm-BF_4_ at 80 °C for the time indicated in Table 1. At the end of the reaction the mixture was extracted with diethyl ether and the extracts were washed with water to obtain the crude, which was analysed using NMR spectroscopy.

**Table 1:** Optimization of the reaction conditions for hydration of diphenylacetylene (**1a**)^a^.

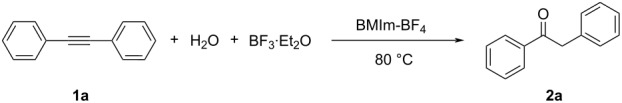						

Entry	BF_3_·Et_2_O^b^	H_2_O^b^	BMIm-BF_4_^c^	Time	Yield **2a** [%]^d^	Recovered **1a** [%]^d^

1^e^	5	–	dried	5 h	53	46
2^f^	5	–	not dried	5 h	73	24
3^e^	5	–	dried	18 h	87	10
4^f^	5	1	dried	5 h	72	24
5^f^	5	1	dried	18 h	95	4
6^f^	5	2	dried	5 h	73	26
7^g^	5	2	dried	18 h	96 (90)^h^	1
8^f^	4	1	dried	18 h	83	13
9^g^	3	1	dried	18 h	81	11
10	3	–	not dried	65 h	92	3
11	2	–	not dried	65 h	66	28

^a^All the reactions were carried out at 80 °C in BMIm-BF_4_, with 0.3 mmol of diphenylacetylene (**1a**); ^b^equivalents with respect to **1a**; ^c^BMIm-BF_4_ was kept under vacuum (7 mbar) for 16 h before each use (dried) or used as such (not dried); ^d^yields calculated from ^1^H NMR spectra of the crude extracts; ^e^the same recycled IL was used for the experiments in entry 1 and 3; ^f^the same recycled IL was used for the experiments in entries 2, 4–6 and 8; ^g^the same recycled IL was used for the experiments in entries 7 and 9; ^h^yield of the product **2a** isolated after column chromatography.

Initially the reaction was carried out without added water, in the presence of a large excess of BF_3_·Et_2_O (5 equiv) (as often reported in literature, see as an example [104]). The reaction was conducted for 5 h at 80 °C using either “stock” (undried) BMIm-BF_4_ (Table 1, entry 2) or “dry” BMIm-BF_4_ (kept under vacuum for 16 h before use, entry 1). Due to the hygroscopic nature of the ILs, the water present within the “stock” BMIm-BF_4_ was evidently enough to give the hydration product **2a** with 73% yield (Table 1, entry 2). However, even in the dried IL, without external addition of water, the product was obtained with 53% yield (Table 1, entry 1), demonstrating that the applied drying process was not sufficient to eliminate all the water present.

By increasing the reaction time, from 5 h to 18 h (Table 1, entry 3), recycling the IL used in the experiment in entry 1 (after drying the IL under vacuum for 16 h), there was a significant increase in product yield from 53% to 87%.

Then, we investigated addition of water to BMIm-BF_4_, as the literature reports that the hydrolysis of the anion of this IL is quite slow in the presence of excess water (less than 5% BF_4_^‒^ hydrolysis in a 1:1 in volume IL/water solution kept at 45 °C for 24 h) [105]. It should be noted that the same treatment carried out on 1-methyl-3-octylimidazolium tetrafluoroborate (OMIm-BF_4_) evidenced a much higher extent of BF_4_^‒^ hydrolysis. This is probably due to the weaker interaction between cation and anion of the IL as the length of the side alkyl chain increases, which makes the BF_4_^‒^/water interaction more effective. Although Saihara and co-workers demonstrated that BF_4_^‒^ hydrolysis generates HF, which reacts with the surrounding glass container yielding SiF_6_^2‒^ (signal at −130 ppm in ^19^F NMR spectrum) [106], we never detected such a peak in ^19^F NMR spectra of the neat IL, analysed after reaction work-up, keeping it under vacuum to completely eliminate diethyl ether traces before NMR analysis. It should be mentioned that the solution was kept in the NMR tubes only for the time necessary to record the NMR spectra. We cannot exclude that a much longer contact time between glass and solution could evidence such a signal.

Using dried IL and adding 1 equiv of water with respect to alkyne (Table 1, entries 4 and 5), the yield of **2a** improved from 53% to 72% after 5 h (Table 1, entry 1 vs 4), and from 87% to 95% after 18 h (Table 1, entry 3 vs 5). Therefore, comparable yields of **2a** can be obtained using the “stock” IL (Table 1, entry 2) or the dried IL by adding 1 equiv of water (Table 1, entry 4). Clearly, the amount of water contained in the IL can be affected by various factors, in particular how long the bottle has been opened and to how much moisture it has been exposed, so from the point of view of reproducibility it was preferred to dry the IL and add a defined amount of water. By increasing the amount of water to 2 equiv, the yields of the desired product did not change (compare Table 1, entries 4 vs 6, and 5 vs 7).

A modest decrease in the yield of **2a** was observed when the amount of BF_3_·Et_2_O was reduced (4 and 3 equiv) in the presence of 1 equiv of water for 18 h, although the yields of the reaction product still remained high (>80%, Table 1, entries 8 and 9). Further investigation using lower amounts of BF_3_·Et_2_O revealed that a 92% yield of **2a** could be realized using 3 equiv of the Lewis acid by extending the reaction time to 65 h (Table 1, entry 10). A further reduction in the amount of BF_3_·Et_2_O to 2 equiv resulted in a lower yield of 66% after the same reaction time (65 h, Table 1, entry 11). The experiments reported in Table 1 suggest that the best conditions for the hydration of diphenylacetylene (**1a**) are 5 equiv of BF_3_·Et_2_O, 1 or 2 equiv of H_2_O, at 80 °C for 18 h (Table 1, entries 5 and 7). Importantly, as shown in Table 1, the same samples of BMIm-BF_4_ were efficiently reused up to five times, without adversely affecting the reaction yields.

### Screening of different ionic liquids as media for the hydration of diphenylacetylene

After the optimization of the reaction conditions in BMIm-BF_4_, different ILs were considered as alternative solvent (Table 2 and Table 3). All the experiments were carried out under the conditions reported in entry 9 of Table 1, in order to observe possible variations in the yield of compound **2a**. ILs with different anions or cations (compared to BMIm-BF_4_) were investigated to probe potential interactions with the reagents, the intermediates or the reaction product. All the ILs were dried under vacuum for 16 h, prior to use.

**Table 2:** Structure of the ILs used as solvent for the hydration reaction of diphenylacetylene (**1a**).

Structure	Acronym	R	R^1^	X^−^

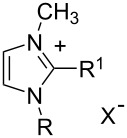	EMIm-BF_4_	CH_3_CH_2_-	H-	BF_4_^−^
	BMIm-BF_4_	CH_3_(CH_2_)_3_-	H-	BF_4_^−^
	HMIm-BF_4_	CH_3_(CH_2_)_5_-	H-	BF_4_^−^
	OMIm-BF_4_	CH_3_(CH_2_)_7_-	H-	BF_4_^−^
	DMIm-BF_4_	CH_3_(CH_2_)_9_-	H-	BF_4_^−^
	BDMIm-BF_4_	CH_3_(CH_2_)_3_-	CH_3_-	BF_4_^−^
	BMIm-Tf_2_N	CH_3_(CH_2_)_3_-	H-	(CF_3_SO_2_)_2_N^−^
	BMIm-PF_6_	CH_3_(CH_2_)_3_-	H-	PF_6_^−^
	BMIm-TfO	CH_3_(CH_2_)_3_-	H-	CF_3_SO_3_^−^
	BMIm-OAc	CH_3_(CH_2_)_3_-	H-	CH_3_COO^−^
	BMIm-OCOCF_3_	CH_3_(CH_2_)_3_-	H-	CF_3_COO^−^

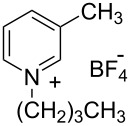	BMPy-BF_4_			

**Table 3:** Hydration reaction of diphenylacetylene **1a** in different ILs^a^.

Entry	Solvent^b^	Yield **2a** [%]^c^	Recovered **1a** [%]^c^

1	EMIm-BF_4_	76	13
2 ^d^	BMIm-BF_4_	81	11
3	HMIm-BF_4_	71	20
4	OMIm-BF_4_	37	54
5	DMIm-BF_4_	31	61
6	BDMIm-BF_4_	64	28
7	BMPy-BF_4_	35	60
8	BMIm-Tf_2_N	87	4
9	BMIm-PF_6_	87	3
10	BMIm-TfO	1	95
11	BMIm-OAc	traces	98
12	BMIm-OCOCF_3_	traces	97
13	dioxane	10	90

^a^All the reactions were carried out with 0.3 mmol of diphenylacetylene (**1a**), 3 equiv of BF_3_·Et_2_O, 1 equiv of H_2_O, at 80 °C for 18 h; ^b^the ILs were kept under vacuum for 16 h before use; ^c^yields calculated from the ^1^H NMR spectra of the crude extracts; ^d^replicate of experiment reported in entry 9 of Table 1, for comparison.

Considering the imidazolium tetrafluoroborate ILs, with the exception of BMIm-BF_4_, a progressive decrease in the yield of **2a**, from 76% to 31%, was observed by increasing the length of the aliphatic chain linked to the imidazolic ring (Table 3, entries 1–5). Although BMIm-BF_4_ gave a slightly higher yield than that obtained with EMIm-BF_4_, the general trend suggests that probably the increase in the lipophilicity of the ILs impairs the reaction, hindering the attack of water to the triple bond. Furthermore, the reaction in BDMIm-BF_4_, with an additional methyl group in 2 position of the imidazolic ring, gave **2a** with a lower yield compared to BMIm-BF_4_ (Table 3, entry 6 vs 2). Replacing the imidazolium cation with 1-butyl-3-methylpyridinium led to a drastic reduction of the yield of **2a**, to 35% (Table 3, entry 7).

By keeping the 1-butyl-3-methylimidazolium cation unchanged, anion variation also affected the reaction yield. Indeed, in the presence of triflate, acetate or trifluoroacetate anions the desired product was obtained only in trace amounts (Table 3, entries 10–12). This could be explained by the fact that these anions could coordinate the Lewis acid BF_3_ through the negatively charged oxygen [107], decreasing availability of BF_3_ for catalysis.

Otherwise, ILs possessing bis(trifluoromeylsulfonyl)imide and hexafluorophosphate anions afforded hydrated product **2a** with slightly better yields (87%) compared to those achieved with the BF_4_^–^ counter anion (Table 3, entries 8–9 vs 2), suggesting PF_6_^−^ and Tf_2_N^−^ do not hinder the reactivity of BF_3_ in the hydration reaction.

Based on these results, considering the higher cost of BMIm-Tf_2_N and BMIm-PF_6_, the preferred IL among those tested, in terms of both yield and cost, is BMIm-BF_4_.

The reaction was also carried out using dioxane as solvent [108]. In this case, the product was obtained with a very low yield of 10% (Table 3, entry 13). This result emphasizes the importance of the use of an IL as a solvent, not only for its green aspect, in particular for its very low vapour pressure and for the possibility of its recycling, but also for its ability to stabilize ionic or polar intermediates, improving the reaction efficiency.

### Hydration of different alkynes catalysed by BF_3_·Et_2_O in BMIm-BF_4_

Subsequently, the developed method was extended to different alkynes, both internal and terminal. The best results for the hydration reaction of each studied alkyne, catalysed by BF_3_·Et_2_O in BMIm-BF_4_, are summarized in Table 4, while all the experiments carried out are reported in Table S1 in Supporting Information File 1. In order to avoid the use of a large excess of the Lewis acid, the conditions reported in entry 9 of Table 1 were chosen as reference for the study of the reactivity of different alkynes.

**Table 4:** Hydration of different alkynes catalysed by BF_3_−Et_2_O in BMIm-BF_4_^a^.

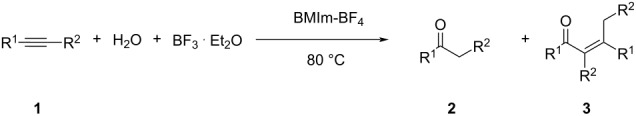					

Entry	Alkyne	BF_3_·Et_2_O^b^	Time	**2**, yield^c^	**3**, yield^c^

1^d^	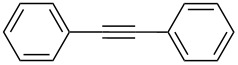	3	18 h	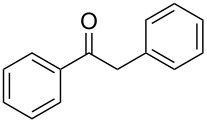	–
	**1a**			**2a**, 81%	

2	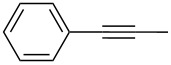	3	5 h	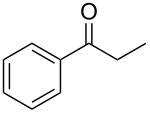	–
	**1b**			**2b**, 97%	

3	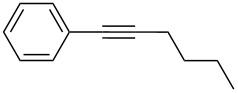	3	5 h	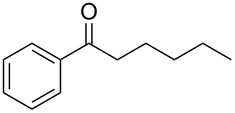	–
	**1c**			**2c**, 76%	

4	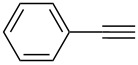	1	1 h	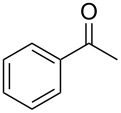	–
	**1d**			**2d**, 81%	

5	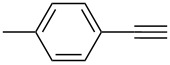	1	1 h	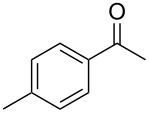	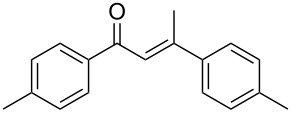
	**1e**			**2e**, 61%	**3e**, 38%

6	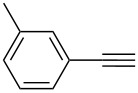	1	1 h	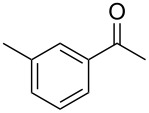	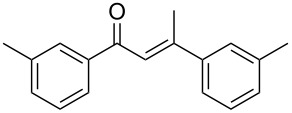
	**1f**			**2f**, 43%	**3f**, 56%

7	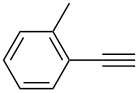	1	1 h	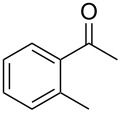	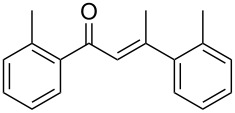
	**1g**			**2g**, 81%	**3g**, 4%

8	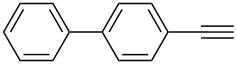	1	1 h	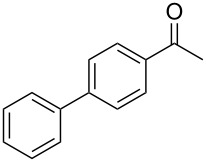	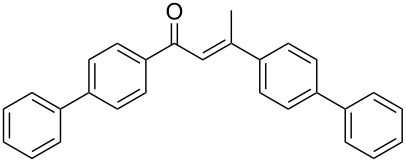
	**1h**			**2h**, 47%	**3h**, 43%

9	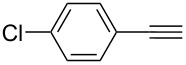	1	1 h	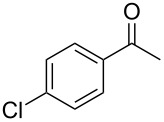	–
	**1i**			**2i**, 72%	

10^e^	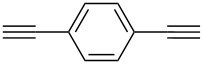 **1j**	2 ^f^	1 h	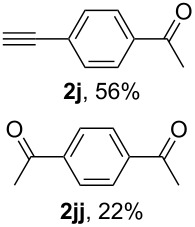	–

11	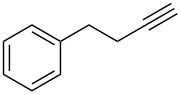	3	18 h	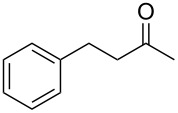	–
	**1k**			**2k**, 79%	

12	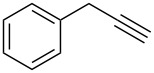	2	18 h	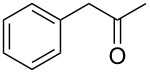	–
	**1l**			**2l**, 62%	

13	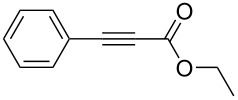	5	18 h	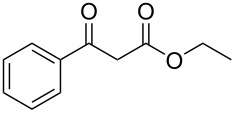	–
	**1m**			**2m**, 65%	

14	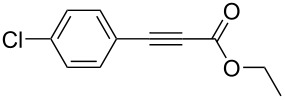	5	18 h	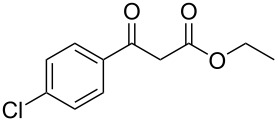	–
	**1n**			**2n**, 47%	

^a^All the reactions were carried out at 80 °C in BMIm-BF_4_, kept under vacuum for 16 h before each use, with 0.3 mmol of alkyne **1** and 0.3 mmol of H_2_O; ^b^equivalents with respect to **1**; ^c^yields calculated from ^1^H NMR spectra of the crude extracts; ^d^replicate of the experiment reported in entry 9 of Table 1; ^e^0.6 mmol of H_2_O were used; ^f^equivalents with respect to one alkyne group of **1j**.

For the internal alkyl(aryl)alkynes a regioselective hydration occurred, with the only generation of the corresponding aryl ketones, formed after the attack of water to the pseudobenzylic position, as observed in Lewis acid-assisted Brønsted acid (LBA) catalysis [47–50]. Internal alkynes afforded the corresponding products in good to excellent yields (Table 4, entries 1–3). In particular, the unsymmetrical alkyl(aryl)alkynes **1b** and **1c** showed a higher reactivity compared to diphenylacetylene (**1a**), affording the corresponding ketones in high yields after 5 h.

Otherwise, terminal alkynes generally showed higher reactivity compared to internal ones. For all the studied terminal alkynes, only ketone products (Markovnikov) were obtained, excluding the formation of the *anti*-Markovnikov ones. Hydration of phenylacetylene **1d** carried out with 3 equiv of BF_3_·Et_2_O for 5 h gave the aldol condensation product **3d** (58%) in addition to acetophenone **2d** with low yield (32%) (see Table S1, Supporting Information File 1). Assuming that enone **3d** is formed from acetophenone, catalysed by the excess Lewis acid present, the reaction was performed in presence of 1 equiv of BF_3_·Et_2_O and a reaction time of 1 h (Table 4, entry 4). In this way, the selectivity was improved and only the hydration product **2d** was obtained in 81% yield.

For electron-rich terminal alkynes, the corresponding ketones could not be selectively obtained without the aldol condensation products. Considering 4-methylphenylacetylene (**1e**), the reaction carried out with 3 equiv of BF_3_·Et_2_O for 5 h gave only the condensation product **3e** (70%, see Table S1, Supporting Information File 1). Reducing the amount of BF_3_·Et_2_O to 1 equiv and the reaction time to 1 h (Table 4, entry 5) gave a mixture of ketones **2e** and **3e** (61% and 38%, respectively). Even reducing the amount of BF_3_·Et_2_O to 0.5 equiv did not improve the yield of the hydration product (see Table S1, Supporting Information File 1). The presence of a methyl group in the *meta* position in **1f** decreased the selectivity with respect to formation of the hydration product **2f**, favouring the condensation product **3f** (Table 4, entry 6). On the other hand, an *ortho* methyl group in **1g** favoured formation of the ketone **2g**, with a good yield, probably due to the steric hindrance of the aldol condensation (Table 4, entry 7). As expected, based on the above consideration, 4-ethynyl-1,1'-biphenyl (**1h**) afforded both hydration and condensation products **2h** and **3h** in similar amounts (Table 4, entry 8), while the presence of a chlorine in the *para* position of the phenyl ring allowed to obtain the hydration product **2i** with good yield, reducing its reactivity (Table 4, entry 9).

With 1,4-diethynylbenzene (**1j**) both the products of mono (**2j**) and bis hydration (**2jj**) were obtained under all conditions tested (see Table S1, Supporting Information File 1). The highest selectivity for the generation of **2j** was achieved with 2 equiv of BF_3_·Et_2_O for 1 h (Table 4, entry 10).

Aliphatic alkyne **1k** showed a different reactivity compared to the other terminal alkynes. Indeed, in this case the corresponding condensation product was never obtained, while the hydration product **2k** was obtained in good yield using 3 equiv of BF_3_·Et_2_O and extending the reaction time to 18 h (Table 4, entry 11).

The aliphatic alkyne **1l** gave the corresponding hydration product **2l** in good yield with 2 equiv of BF_3_·Et_2_O and a reaction time of 18 h (Table 4, entry 12).

The following step was to study the reactivity of BF_3_·Et_2_O in BMIm-BF_4_ towards disubstituted alkynes containing a carbonyl group adjacent to the triple bond. This class of substrates, after water addition, yields 1,3-dicarbonyl compounds, which could yield BF_2_-chelates under our experimental conditions [109]. In order to study their behaviour, we decided to avoid water during an initial work-up, to prevent a possible BF_2_-chelate hydrolysis, and only ethereal extraction was carried out after the reaction.

When the reaction was carried out on ethyl 3-phenylpropiolate (**1m**, Table 4, entry 13), the analysis of the ethereal extracts showed the presence of the BF_2_-chelate. In fact, the following convincing peaks were found in the NMR spectra: a singlet at 6.11 ppm, along with a quartet at 4.68 ppm (^1^H NMR spectrum), a peak at 83.3 ppm (^13^C NMR spectrum) and a singlet at −139.1 ppm (^19^F NMR spectrum) [109]. A simple washing with distilled water gave the corresponding ethyl benzoylacetate (**2m**). Compared to the other studied alkynes, **1m** required a larger excess of BF_3_·Et_2_O (5 equiv) to give the corresponding hydration product with a satisfactory yield. Indeed, this behaviour could be explained by the formation of the BF_2_-chelate, which reduces the amount of BF_3_ available for catalysis.

A similar behaviour was observed with ethyl 3-(4-chlorophenyl)propiolate (**1n**), although with lower yield due to the deactivating effect of the chlorine substituent in the *para* position of the phenyl ring (Table 4, entry 14).

Importantly, for the experiments involving the same alkyne (see Table S1, Supporting Information File 1), the same sample of BMIm-BF_4_ was effectively reused, up to five times, demonstrating the advantage of using this IL as medium for this reaction.

### Hydration of alkynes in electrogenerated BF_3_/BMIm-BF_4_ system

Based on previous works, which demonstrated the possibility to electrogenerate BF_3_ in tetrafluoroborate ILs [100], and to efficiently use it to carry out different Lewis acid catalysed organic reactions [101–103], we investigated the applicability of the electrogenerated BF_3_/BMIm-BF_4_ system for the hydration reaction of alkynes. The best results for the hydration reaction of each studied alkyne, catalysed by electrogenerated BF_3_ in BMIm-BF_4_, are summarized in Table 5, while all the experiments carried out are reported in Table S2 in the Supporting Information File 1.

**Table 5:** Hydration of different alkynes catalysed by electrogenerated BF_3_ in BMIm-BF_4_.^a^

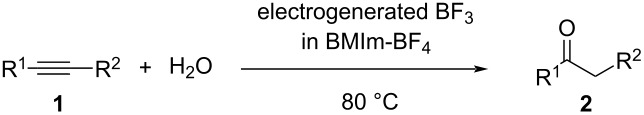				

Entry	Alkyne	Electrogenerated BF_3_ (F/mol)^b^	Time	Product **2**, yield^c^

1	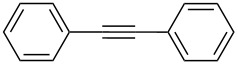	4	18 h	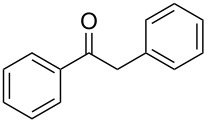
	**1a**			**2a**, 85%

2	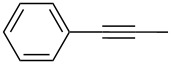	4	5 h	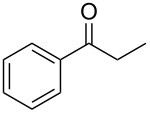
	**1b**			**2b**, 84%

3	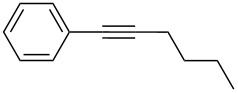	4	5 h	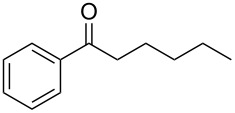
	**1c**			**2c**, 94%

4	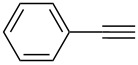	1	1 h	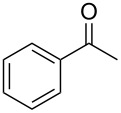
	**1d**			**2d**, 78%

5	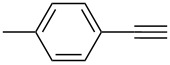	1	1 h	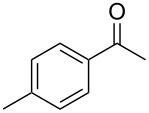
	**1e**			**2e**, 84%^d^

6	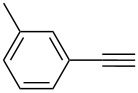	1	1 h	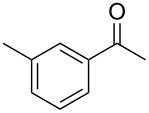
	**1f**			**2f**, 91%

7	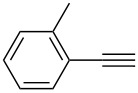	1	1 h	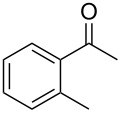
	**1g**			**2g**, 78%

8	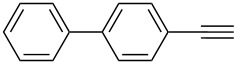	1	1 h	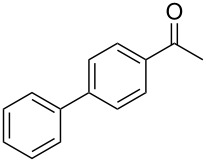
	**1h**			**2h**, 94%

9	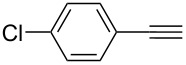	2	18 h	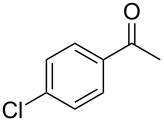
	**1i**			**2i**, 79%

10^e^	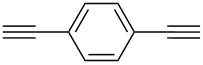 **1j**	4	5 h	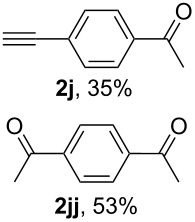

11	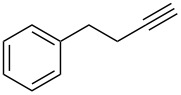	2	18 h	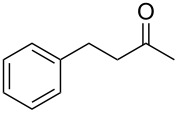
	**1k**			**2k**, 51%

12	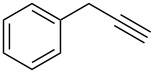	4	18 h	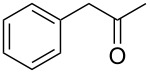
	**1l**			**2l**, 23%

13	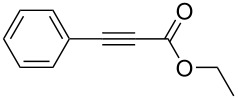	4^f^	18 h	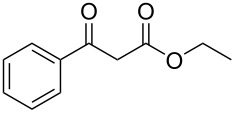
	**1m**			**2m**, 58%

^a^BMIm-BF_4_, kept under vacuum for 16 h before each use, was electrolyzed (galvanostatic conditions: 10 mA·cm^−2^) on platinum electrodes (rt, N_2_) in divided cell configuration. At the end of electrolysis, alkyne **1** (0.3 mmol) and H_2_O (0.3 mmol) were added to the anolyte. All the reactions were carried out at 80 °C for the time reported in table; ^b^amount of electrogenerated BF_3_ with respect to starting alkyne, admitting a 100% current efficiency (1 mF = 96.5 C = 1 mmol of BF_3_); ^c^yields calculated from ^1^H NMR spectra of the crude extracts; ^d^**3e**, 9%. ^e^0.6 mmol of H_2_O were used. ^f^the electrolysis was carried out in the presence of the alkyne (0.3 mmol) in the anodic compartment. At the end of electrolysis, H_2_O (0.3 mmol) was added to the anolyte, then the reaction was carried out at 80 °C for the time reported in table.

**Figure 1:**
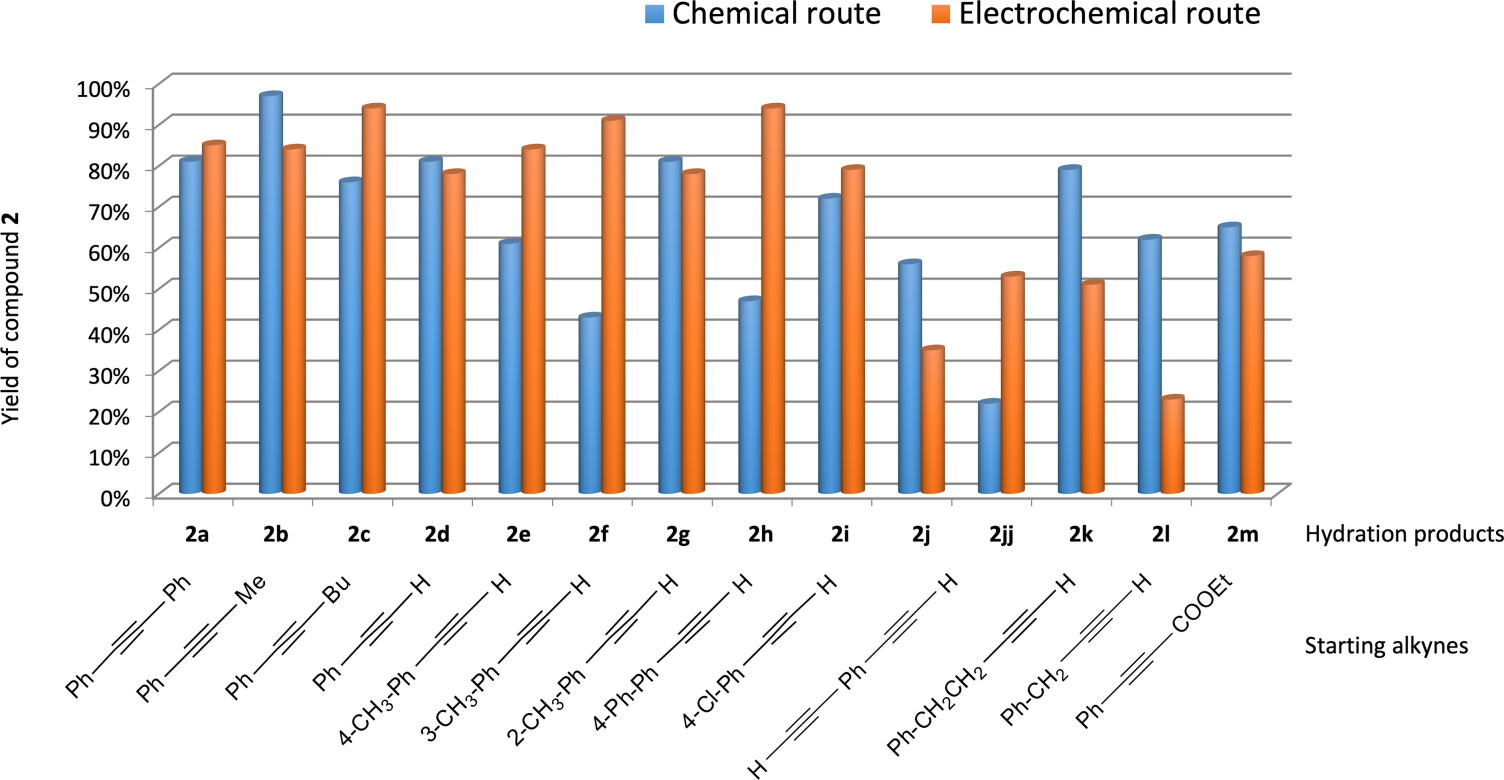
Comparison of the hydration reactions of different alkynes in BMIm-BF_4_ catalysed by BF_3_·Et_2_O (blue) and by electrogenerated BF_3_ (orange).

Regarding internal alkynes, the electrogenerated BF_3_ (4 F/mol)/BMIm-BF_4_ system proved to be highly efficient for **1a**, **1b** and **1c**, delivering the corresponding ketones in excellent yields, which were comparable or better than those achieved using BF_3_·Et_2_O (Table 5, entries 1–3, and Figure 1).

In contrast to earlier results, an interesting behaviour was observed with the terminal alkynes. Indeed, the terminal arylalkynes **1d–h** afforded the corresponding hydration products selectively in good to excellent yields by exploiting the electrogeneration of BF_3_ in BMIm-BF_4_ at 1 F/mol (Table 5, entries 4–8). It is important to remember that with BF_3_·Et_2_O these alkynes gave mixtures with the corresponding aldol condensation products, in some cases in considerable amounts (Table 4, entries 5, 6, and 8). Reduction in the amount of BF_3_·Et_2_O to 0.5 equiv did not improve the yields of the hydration products (see Table S1, Supporting Information File 1).

Interestingly, the alkynes **1e**, **1f** and **1h**, which in the chemical route provided considerable amounts of the condensation products and moderate yields for the hydration products, with the electrochemical route gave the corresponding hydration products with excellent yields, significantly better compared to those obtained with BF_3_·Et_2_O (Table 5, entries 5, 6, 8, and Figure 1). By exploiting the electrochemical generation of BF_3_, the alkynes **1d** and **1g** gave the corresponding ketones with similar yields compared to the chemical route (Table 5, entries 4 and 7, and Figure 1). The alkyne **1i** gave the ketone **2i** with a slightly better yield compared to the chemical route, when increasing the amount of the electrogenerated BF_3_ to 2 F/mol and the reaction time to 18 h (Table 5, entry 9, and Figure 1).

The application of the electrochemical conditions to 1,4-diethynylbenzene (**1j**) using 2 F/mol selectively afforded ketone **2j** after 1 h, after hydration of one alkyne group, in low yield (39%), with the majority of the starting alkyne being recovered (46%) (see Table S2, Supporting Information File 1). Increasing the amount of electrogenerated BF_3_ by applying 4 F/mol and extending the reaction time (5 h) reversed the selectivity in favour of the diketone **2jj** (Table 5, entry 10), which had not been achieved using BF_3_·Et_2_O as a reagent (see Table 4, entry 10).

For the aliphatic alkyne **1k** the hydration product **2k** was obtained with moderate yield by exploiting the electrogeneration of 2 F/mol of BF_3_ (Table 5, entry 11). Unfortunately, an increase in the amount of electrogenerated BF_3_ did not improve the yield of the desired product (see Table S2, Supporting Information File 1).

By exploiting the electrogeneration of 4 F/mol of BF_3_, with the aliphatic alkyne **1l** the corresponding hydration product **2l** was obtained with low yield (Table 5, entry 12).

Unfortunately, any attempt to hydrate disubstituted alkynes containing a carbonyl group adjacent to the triple bond (**1m**, **1n**) with electrogenerated BF_3_, according to the procedure adopted for the other alkynes, failed, yielding only starting material.

We then tried to electrogenerate BF_3_ in BMIm-BF_4_, directly in the presence of the alkyne **1m** or **1n** in the anodic compartment. Surprisingly, for alkyne **1m** this approach has allowed to obtain the hydration product **2m**, with a yield (58%) slightly lower than that observed in the chemical route (Table 5, entry 13, and Figure 1). Otherwise, with the alkyne **1n**, also in this way, the hydration product was not obtained. In addition to the different reactivity, due to the presence or not of chlorine in the *para* position of the phenyl group, the different physical state (liquid for **1m** vs solid for **1n**) and the possible different solubility in BMIm-BF_4_ at room temperature (according to the electrolysis conditions) may have affected the results obtained with these substrates. Further studies will be necessary to clarify the behaviour of alkynes containing a carbonyl group adjacent to the triple bond.

After work-up, the electrolysed IL was placed under vacuum to eliminate diethyl ether traces and then analysed by NMR to check for BMIm-F presence, whereas the fluoride ion could originate from IL decomposition in the presence of water or from the evolution of electrogenerated F_2_. However, the ^19^F NMR spectrum showed no detectable peak around −122 ppm, reported in the literature for BMIm-F [110]. The only difference between IL ^19^F NMR spectra before and after electrolysis is a peak at ‒148.7 ppm (referred to BF_4_^−^ at −150.6 ppm), possibly due to BF_3_OH^−^ or B_2_F_7_^−^ [111–112] (see Supporting Information File 1, Figure S1a vs f and b, c). This last hypothesis is corroborated by the ^19^F NMR analysis of BMIm-BF_4_ after anodic oxidation in a divided cell, which shows a peak at −147.3 ppm (besides the peak at −150.6 due to BF_4_^−^) (see Supporting Information File 1, Figure S1e), which is replaced by a peak at −144.0 ppm (referred to −150.6 ppm for BF_4_^−^) when the electrolysis is carried out in an undivided cell (see Supporting Information File 1, Figure S1d). In this last case, in fact, the NHC-BF_3_ adduct is formed between anodically electrogenerated BF_3_ and cathodically electrogenerated NHC [103].

### Evaluation of the current efficiency in the electrogeneration of BF_3_ in BMIm-BF_4_

**Scheme 1:**
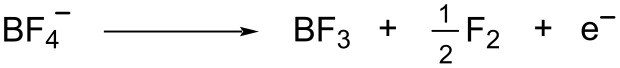
Anodic oxidation of tetrafluoroborate anion.

In order to have an idea of the current efficiency in the electrogeneration of BF_3_ in BMIm-BF_4_ (a monoelectronic process, Scheme 1), we tried to quantitatively capture the electrogenerated BF_3_ with a tertiary base just at the end of the electrolysis.

By a comparison between the ^13^C NMR peaks of the base and the base–BF_3_ adduct, we should obtain an approximate current yield. Our first choice was *N*,*N*-diisopropylethylamine (DIPEA), as the DIPEA-BF_3_ adduct is reported in the literature and fully characterized by NMR in CDCl_3_ [113]. To be consistent with literature data, the BF_4_^−^ peak in neat BMIm-BF_4_ was set at −150.6 ppm in ^19^F NMR spectrum [112].

We thus carried out the anodic oxidation of pure BMIm-BF_4_ (divided cell, galvanostatic conditions) and stopped the electrolysis after 60 C (corresponding to 0.6 mmol of electrons). At the end of the electrolysis, 0.6 mmol of DIPEA were added to the anolyte and the mixture was kept under stirring at room temperature for 30 min. Then, the neat anolyte was analysed by NMR (^19^F and ^13^C). The ^19^F NMR spectrum showed a new peak at −148.7 ppm and, to our great astonishment, we found only one set of signals in the ^13^C NMR spectrum (55.0, 42.8, 17.4, 16.0, 12.2), apart from those of the IL cation (see Supporting Information File 1, Figure S2). These signals are quite different from those of DIPEA in CDCl_3_ (48.5, 39.1, 20.6, 17.1) [114] (the ^13^C NMR spectrum of DIPEA in pure BMIm-BF_4_ is not reported), but quite similar to the ^13^C NMR spectrum of DIPEA-BF_3_ adduct in CDCl_3_ (53.8, 41.6, 19.5, 18.9, 9.9), inducing us to think to have the DIPEA-BF_3_ adduct in the solution. To confirm this assumption, we prepared a DIPEA solution in BMIm-BF_4_ to record the ^13^C NMR spectrum, but unfortunately DIPEA is not soluble enough in BMIm-BF_4_ to obtain a decent spectrum. Therefore, while confirming the presence of the adduct, we could not quantify it.

The next choice was DBU (1,8-diazabicyclo[5,4,0]undec-7-ene). The DBU-BF_3_ adduct is reported to be very stable in water and in air and not subjected to hydrolysis [115]. The DBU solubility in BMIm-BF_4_ was confirmed by NMR analysis (amidine carbon atom at 161.6 ppm in BMIm-BF_4_, taking as internal reference the imidazolium C2 at 136.4 ppm) [116]. The addition of an excess of BF_3_·Et_2_O to the solution of DBU in IL shifted the DBU amidine signal to 166.0 ppm, confirming the rapid formation of the adduct (see Supporting Information File 1, Figure S5c). Moreover, a new small peak at −146.1 ppm appeared in the ^19^F NMR spectrum [115], in addition to the peaks at −150.6 ppm (BF_4_^−^ signal), at −148.7 ppm (BF_3_OH^‒^) and −153.6 ppm (BF_3_·Et_2_O) (see Supporting Information File 1, Figures S3 and S4).

We thus carried out the anodic oxidation of pure BMIm-BF_4_ (divided cell, galvanostatic conditions) and stopped the electrolysis after 60 C (corresponding to 0.6 mmol of electrons). At the end of the electrolysis, 0.6 mmol of DBU were added to the anolyte and the mixture was kept under stirring at room temperature for 30 min. Then the neat anolyte was analysed by NMR (^19^F and ^13^C). A peak at 166.0 ppm in the ^13^C NMR spectrum appeared and no traces of starting DBU (peak at 161 ppm) were evidenced (see Supporting Information File 1, Figure S5a, b and d). As regards the ^19^F NMR spectrum, a new peak at −148.6 ppm appeared, consistent with the formation of B_2_F_7_^−^ or with the DBU-BF_3_ adduct (a direct comparison with literature data is not possible in this case, as the NMR data reported in previous papers were obtained in molecular solvents, while we carried out the experiments in pure ionic liquid) [115].

To our surprise, the addition of additional DBU to this solution did not show the signal of DBU in the ^13^C NMR spectrum (161 ppm), but increased the 166 ppm peak intensity (due to the DBU adduct) (see Supporting Information File 1, Figure S5e). We have no explanation for this behaviour, but the possibility of the coordination of more than one DBU molecule could be a hypothesis. In this regard, Hartman and co-workers reported the formation of BF_x_DBU_y_ positively charged adducts (y from 1 to 3) [115]. Although we cannot exclude that the signal is due to the [DBU-H]^+^, the ^13^C NMR of the reaction mixture did not highlight the presence of the NHC derived from the IL deprotonation.

## Conclusion

In conclusion, in this work we demonstrated the possibility to carry out the hydration of alkynes in imidazolium ILs, as alternative solvents until now still little explored for this reaction, employing the Lewis acid BF_3_ as catalyst. The catalyst was used both as BF_3_·Et_2_O and as BF_3_ directly electrogenerated in the IL. Among the investigated ILs, BMIm-BF_4_ provided the best reaction yields and is preferred on the basis of cost. The results obtained with BF_3_·Et_2_O were compared with those achieved using BF_3_ electrogenerated in BMIm-BF_4_, demonstrating the possibility of employing a less harmful system to obtain the products of alkyne hydration with analogous or improved yields. On the basis of the results obtained with the studied substrates, the electrochemical route would appear to be more advantageous for the more reactive terminal arylalkynes, in terms of selectivity and, in some cases, of yield.

The possibility of recycling the ionic liquid for subsequent reactions was successfully demonstrated, confirming the advantage of using BMIm-BF_4_ as a green solvent for this reaction.

Together, these results demonstrate the promise of BMIm-BF_4_/BF_3_ (either with electrogenerated BF_3_ or with BF_3_·Et_2_O) as an efficient and less harmful alternative to expensive metal/ligand catalysts, while avoiding conventional toxic and volatile solvents commonly used for the hydration of alkynes.

## Experimental

### General Information

All chemicals were commercial (Fluorochem, Aldrich) and used without further purification. Ionic liquids (ILs, Iolitec) were kept under vacuum (7 mbar) under stirring at 40 °C for 16 h before use. NMR spectra were recorded at ambient temperature on Bruker Avance spectrometer operating at 400 MHz (^1^H NMR) and 100 MHz (^13^C{^1^H} NMR) or on a Spinsolve 60 spectrometer operating at 62.5 MHz (^1^H NMR), 15.7 MHz (^13^C{^1^H} NMR) and 58.8 MHz (^19^F NMR) using the solvent as internal standard. All the NMR spectra of neat IL were performed on Spinsolve 60 spectrometer. The chemical shifts (δ) are given in ppm relative to TMS. Flash chromatography was carried out using silica (Merck; 40–63 μm particle size).

### General procedures

#### General procedure for the hydration of alkynes catalysed by BF_3_·Et_2_O in ILs

In a 10 mL vial, 1 mL of the IL, a magnetic stirring bar and the amount of alkyne, water and BF_3_·Et_2_O reported in Tables 1, 3, and 4 were added. The vial was sealed with a screw cap and the mixture was stirred at 80 °C in an oil bath. After the time indicated in Tables 1, 3, and 4, the mixture was extracted with diethyl ether (3 × 8 mL). The combined organic phase was washed with water (3 × 20 mL), dried on Na_2_SO_4_, filtered and then the solvent was removed under reduced pressure. The crude was analysed by ^1^H NMR and ^13^C NMR and then the products were purified by column chromatography.

#### General procedure for the electrochemical generation of BF_3_ in BMIm-BF_4_

All the experiments were carried out in a home-made divided glass cell separated through a porous glass plug; Pt spirals (apparent area 0.8 cm^2^) were used as anode and cathode. 2.0 mL of BMIm-BF_4_ and the magnetic stirring bar were put in the anodic compartment (test tube, *h* = 10.5 cm, *d* = 1.7 cm), and 1.0 mL of the same IL in the cathodic one. Electrolyses were performed at constant current (*I* = 10 mA·cm^−2^), under stirring at room temperature, under nitrogen atmosphere, using an Amel Model 552 potentiostat equipped with an Amel Model 731 integrator. When the desired Coulombs (reported in Table 5) had passed through the electrolysis cell, the current was switched off, the cathodic compartment removed and the amounts of alkyne and water reported in Table 5 were added to the anolyte. The test tube was sealed with a rubber cap and the mixture was stirred at 80 °C in an oil bath. After the time indicated in Table 5, the mixture was extracted with diethyl ether (3 × 8 mL). The combined organic phase was washed with water (3 × 20 mL), dried on Na_2_SO_4_, filtered and then the solvent was removed under reduced pressure. The crude was analysed by ^1^H NMR and ^13^C NMR and then the products were purified by column chromatography.

### Procedure for the evaluation of the current efficiency in the electrogeneration of BF_3_ in BMIm-BF_4_

Electrolyses were performed as reported above and stopped after the passage of 60 C. At the end of the electrolysis the cathodic compartment was removed and 0.6 mmol of the appropriate tertiary amine (DIPEA or DBU) were added to the anolyte. The mixture was stirred at room temperature under inert atmosphere (N_2_) for 30 min. Then the neat IL was analysed by ^13^C NMR and ^19^F NMR on Spinsolve 60 spectrometer. For the experiment with DBU, after the analysis of the sample thus prepared, another aliquot of DBU was added directly into the NMR test tube (about 0.1 mmol of DBU for 0.5 mL of IL).

The reference DIPEA/BMIm-BF_4_ or DBU/BMIm-BF_4_ solutions were prepared by mixing 0.1 mmol of the appropriate base with 0.5 mL of BMim-BF_4_.

### Recycling of ILs

The IL sample already used was recycled after the elimination of diethyl ether and water, by keeping the IL under vacuum (7 mbar) under stirring at 40 °C for 16 h.

### Procedure for the hydration of diphenylacetylene in dioxane

Water (0.3 mmol) and BF_3_·Et_2_O (0.9 mmol) were added to a solution of diphenylacetylene (**1a**, 0.3 mmol) in dioxane (2 mL) in a 5 mL flask. The reaction mixture was stirred at 80 °C in an oil bath for 18 h. Then, the reaction mixture was diluted with diethyl ether (20 mL) and washed with water (3 × 20 mL). The organic phase was dried on Na_2_SO_4_, filtered and then the solvent was removed under reduced pressure. The crude was analysed by ^1^H NMR and ^13^C NMR.

## Supporting Information

Integral tables of the experiments for the hydration of different alkynes catalysed by BF_3_·Et_2_O or by electrogenerated BF_3_ in BMIm-BF_4_. ^19^F and ^13^C NMR spectra for the evaluation of the current efficiency in the electrogeneration of BF_3_ in BMIm-BF_4_. Analytical data, ^1^H and ^13^C NMR spectra of synthetized compounds.

File 1 Additional experimental data.
