# Supplementary material for: 1-Butyl-3-methylimidazolium tetrafluoroborate as suitable solvent for BF3: the case of alkyne hydration. Chemistry vs electrochemistry
Source: Beilstein J Org Chem. 2023 Dec 28;19:1966–81. doi: 10.3762/bjoc.19.147 (PMC10760484; doi:10.3762/bjoc.19.147)
Supplement: File 1 — Additional experimental data. [file Beilstein_J_Org_Chem-19-1966-s001.pdf]

BEILSTEIN JOURNAL OF ORGANIC CHEMISTRY

Supporting Information

for

1-Butyl-3-methylimidazolium tetrafluoroborate as suitable solvent for BF_3: the case of alkyne hydration. Chemistry vs electrochemistry

Marta David, Elisa Galli, Richard C. D. Brown, Marta Feroci, Fabrizio Vetica and Martina Bortolami

Beilstein J. Org. Chem. **2023,** *19,* 1966–1981. doi:10.3762/bjoc.19.147

Additional experimental data

License and Terms: This is a supporting information file under the terms of the Creative Commons Attribution License (https://creativecommons.org/licenses/by/4.0). Please note that the reuse, redistribution and reproduction in particular requires that the author(s) and source are credited and that individual graphics may be subject to special legal provisions.
The license is subject to the *Beilstein Journal of Organic Chemistry* terms and conditions: (https://www.beilstein-journals.org/bjoc/terms)

Table of contents

Table S1. Hydration of different alkynes catalysed by $BF_3{\cdot}Et_2O$ in BMIm-BF_4............................... S2

Table S2. Hydration of different alkynes catalysed by electrogenerated BF_3 in BMIm-BF_4............ S4

Figure S1. ^{19}F-NMR spectra under neat conditions of (a) BMIm-BF_4, (b) $BF_3{\cdot}Et_2O$ in BMIm-BF_4, (c) $BF_3{\cdot}Et_2O$ and H_2O in BMIm-BF_4, (d) anodically oxidated BMIm-BF_4 in a undivided cell, (e) anodically oxidated BMIm-BF_4 in a divided cell, (f) recycled BMIm-BF_4 after anodical oxidation in a divided cell, reaction with diphenylacetylene, ethereal extraction and solvent evaporation........... S6

Figure S2. ^{13}C NMR spectra under neat conditions of (a) BMIm-BF_4, (b) DIPEA (0.6 mmol) added to anodically oxidated BMIm-BF_4 (60 C) in a divided cell.. S7

Figure S3. ^{19}F NMR spectra under neat conditions of (a) DBU and $BF_3{\cdot}Et_2O$ in BMIm-BF_4, (b) DBU (0.6 mmol) added to anodically oxidated BMIm-BF_4 (60 C) in a divided cell................................. S8

Figure S4. ^{19}F NMR spectra under neat conditions of DBU and $BF_3{\cdot}Et_2O$ in BMIm-BF_4................ S9

Figure S5. ^{13}C NMR spectra under neat conditions of (a) BMIm-BF_4, (b) DBU in BMIm-BF_4, (c) $BF_3{\cdot}Et_2O$ and DBU in BMIm-BF_4, (d) DBU (0.6 mmol) in anodically oxidated BMIm-BF_4 (60 C) in a divided cell, (e) DBU (1.2 mmol) in anodically oxidated BMIm-BF_4 (60 C) in a divided cell..... S10

Analytical data.. S11

^{1}H and ^{13}C{^{1}H} NMR spectra in $CDCl_3$.. S19

References.. S40

Table S1. Hydration of different alkynes catalysed by $BF_3 \cdot Et_2O$ in BMIm-BF_4. [a]

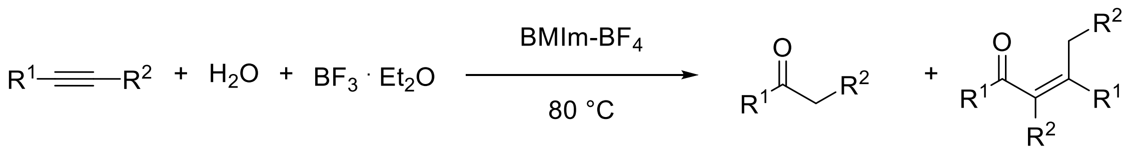

Entry	Alkyne	$BF_3 \cdot Et_2O$ [b]	Time	**2**, yield [c]	**3**, yield [c]	Recovered **1** [c]
1 [d]	**1a**	3	18 h	**2a**, 81%	-	11%
2	**1b**	3	5 h	**2b**, 97%	-	-
3	**1b**	3	18 h	**2b**, 100%	-	-
4	**1c**	3	5 h	**2c**, 76%	-	-
5	**1d**	3	5 h	**2d**, 32%	**3d**, 58%	-
6	**1d**	1	1 h	**2d**, 81%	-	-
7	**1e**	3	5 h	-	**3e**, 70%	-
8	**1e**	3	18 h	-	**3e**, 36%	-
9	**1e**	2	5 h	**2e**, 21%	**3e**, 74%	-
10	**1e**	1	1 h	**2e**, 61%	**3e**, 38%	-
11	**1e**	0.5	1 h	**2e**, 49%	**3e**, 31%	-
12	**1f**	1	1 h	**2f**, 43%	**3f**, 56%	-
13	**1f**	0.5	1 h	**2f**, 26%	-	37%
14	**1g**	1	1 h	**2g**, 81%	**3g**, 4%	-
15	**1h**	1	1 h	**2h**, 47%	**3h**, 43%	-
16	**1i**	1	1 h	**2i**, 72%	-	21%
17	**1i**	1	2 h	**2i**, 64%	**3i**, 29%	6%
18 [e]	**1j**	5 [f]	5 h	**2j**, 21%<br>**2jj**, 14%	-	5%

19 [e]	**1j**	5 [f]	18 h	**2j**, 13%<br>**2jj**, 14%	-	-
20 [e]	**1j**	3 [f]	1 h	**2j**, 57%<br>**2jj**, 16%	-	18%
21 [e]	**1j**	3 [f]	5 h	**2j**, 16%<br>**2jj**, 29%	-	2%
22 [e]	**1j**	2 [f]	1 h	**2j**, 56%<br>**2jj**, 22%	-	6%
23	**1k**	3	18 h	**2k**, 79%	-	-
25	**1l**	2	1 h	**2l**, 13%	-	63%
26	**1l**	2	18 h	**2l**, 62%	-	-
27	**1m**	5	18 h	**2m**, 65%	-	25%
28	**1m**	3	5 h	**2m**, 26%	-	73%
29	**1n**	5	18 h	**2n**, 47%	-	48%
30	**1n**	3	18 h	**2n**, 23%	-	68%

[a] All the reactions were carried out at 80 °C in BMIm-BF_4, kept under vacuum for 16 h before each use, with 0.3 mmol of alkyne **1** and 0.3 mmol of H_2O. [b] Equivalents with respect to **1**. [c] Yields calculated from ^{1}H NMR spectra of the crude extracts. [d] Replicate of experiment reported in entry 9 of Table 1, for comparison. [e] 0.6 mmol of H_2O were used. [f] Equivalents with respect to one alkyne group of **1j**.

Table S2. Hydration of different alkynes catalysed by electrogenerated BF_3 in BMIm-BF_4. [a]

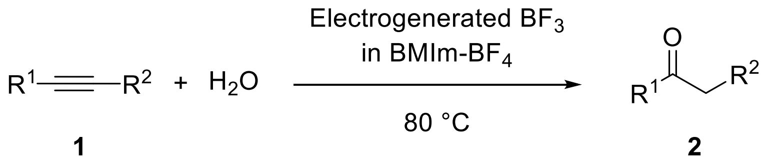

1 **2**

Entry	Alkyne	Electrogenerated BF_3 (F/mol) [b]	Time	**2**, yield [c]	Recovered **1** [c]
1	**1a**	4	18 h	**2a**, 85%	3%
2	**1b**	4	5 h	**2b**, 84%	-
3	**1c**	4	5 h	**2c**, 94%	-
4	**1d**	1	1 h	**2d**, 78%	-
5	**1e**	1	1 h	**2e**, 84% [d]	-
6	**1f**	1	1 h	**2f**, 91%	-
7	**1g**	1	1 h	**2g**, 78%	-
8	**1h**	1	1 h	**2h**, 94%	-
9	**1i**	1	1 h	**2i**, 20%	61%
10	**1i**	1	18 h	**2i**, 40%	12%
11	**1i**	2	18 h	**2i**, 79%	-
12 [e]	**1j**	2	1 h	**2j**, 39%	46%
13 [e]	**1j**	4	5 h	**2j**, 35%<br>**2jj**, 53%	3%
14	**1k**	1	5 h	-	54%
15	**1k**	2	5 h	**2k**, 13%	73%
16	**1k**	2	18 h	**2k**, 51%	-
17	**1k**	4	18 h	**2k**, 45%	-
18	**1l**	1	1 h	-	68%

19	**1l**	2	18 h	**2l**, 11%	43%
20	**1l**	4	18 h	**2l**, 23%	33%
21	**1m**	4	18 h	**-**	100%
22	**1m**	4 [f]	18 h	**2m**, 58%	40%
23	**1n**	4	18 h	**-**	100%
24	**1n**	8	18 h	**2n**, 2%	78%
25	**1n**	4 [f]	18 h	**-**	100%

[a] BMIm-BF_4, kept under vacuum for 16 h before each use, was electrolyzed (galvanostatic conditions: 10 mA·cm^{-2}) on platinum electrodes (rt, N_2) in divided cell configuration. At the end of electrolysis, alkyne **1** (0.3 mmol) and H_2O (0.3 mmol) were added to the anolyte. All the reactions were carried out at 80 °C for the time reported in table. [b] Amount of electrogenerated BF_3 with respect to starting alkyne, admitting a 100% current efficiency (1 mF = 96.5 C = 1 mmol of BF_3). [c] Yields calculated from ^{1}H NMR spectra of the crude extracts. [d] **3e**, 9%. [e] 0.6 mmol of H_2O were used. [f] The electrolysis was carried out in the presence of the alkyne (0.3 mmol) in the anodic compartment. At the end of electrolysis, H_2O (0.3 mmol) was added to the anolyte, then the reaction was carried out at 80 °C for the time reported in the table.

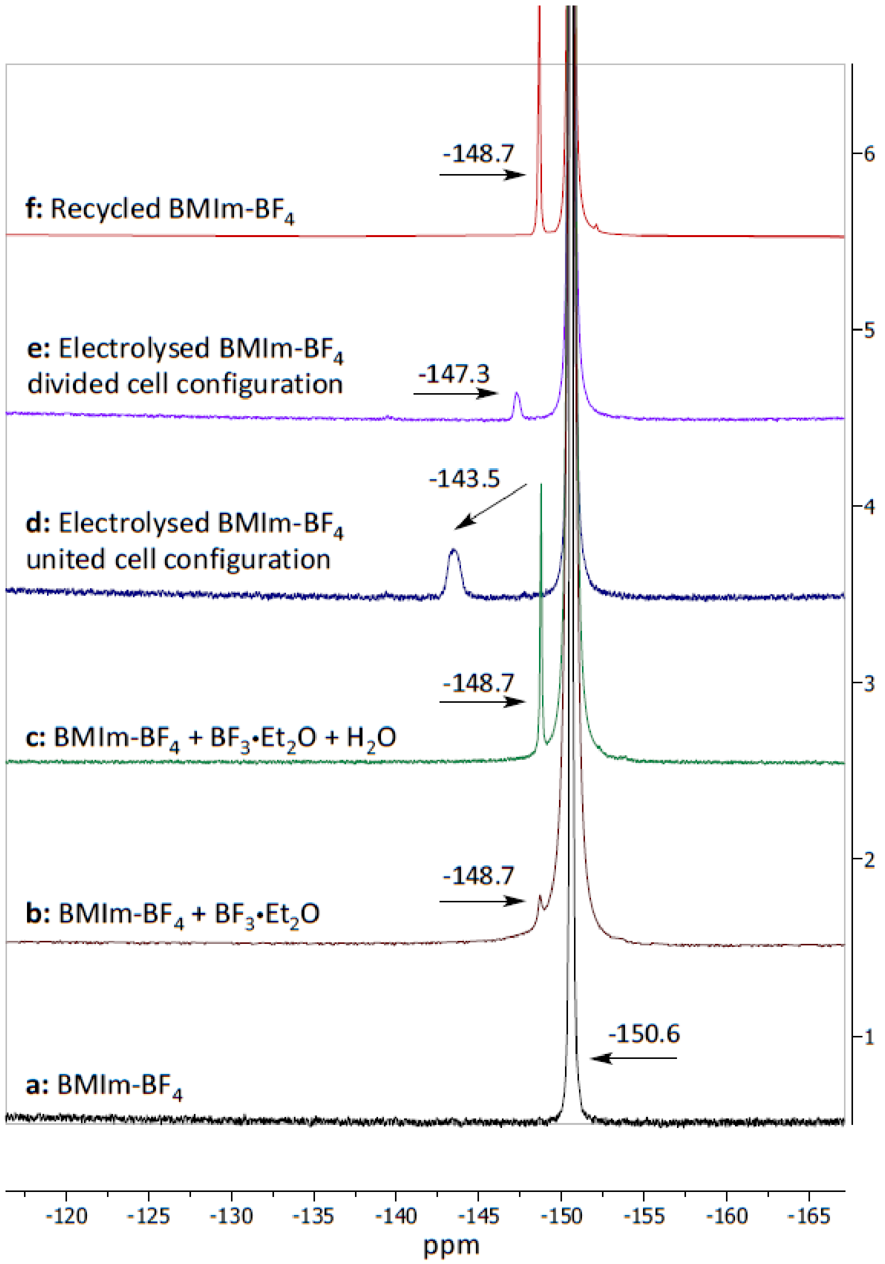

Figure S1. ^{19}F NMR spectra under neat conditions of (a) BMIm-BF_4, (b) $BF_3 \cdot Et_2O$ in BMIm-BF_4, (c) $BF_3 \cdot Et_2O$ and H_2O in BMIm-BF_4, (d) anodically oxidated BMIm-BF_4 in a undivided cell, (e) anodically oxidated BMIm-BF_4 in a divided cell, (f) recycled BMIm-BF_4 after anodical oxidation in a divided cell, reaction with diphenylacetylene, ethereal extraction and solvent evaporation.

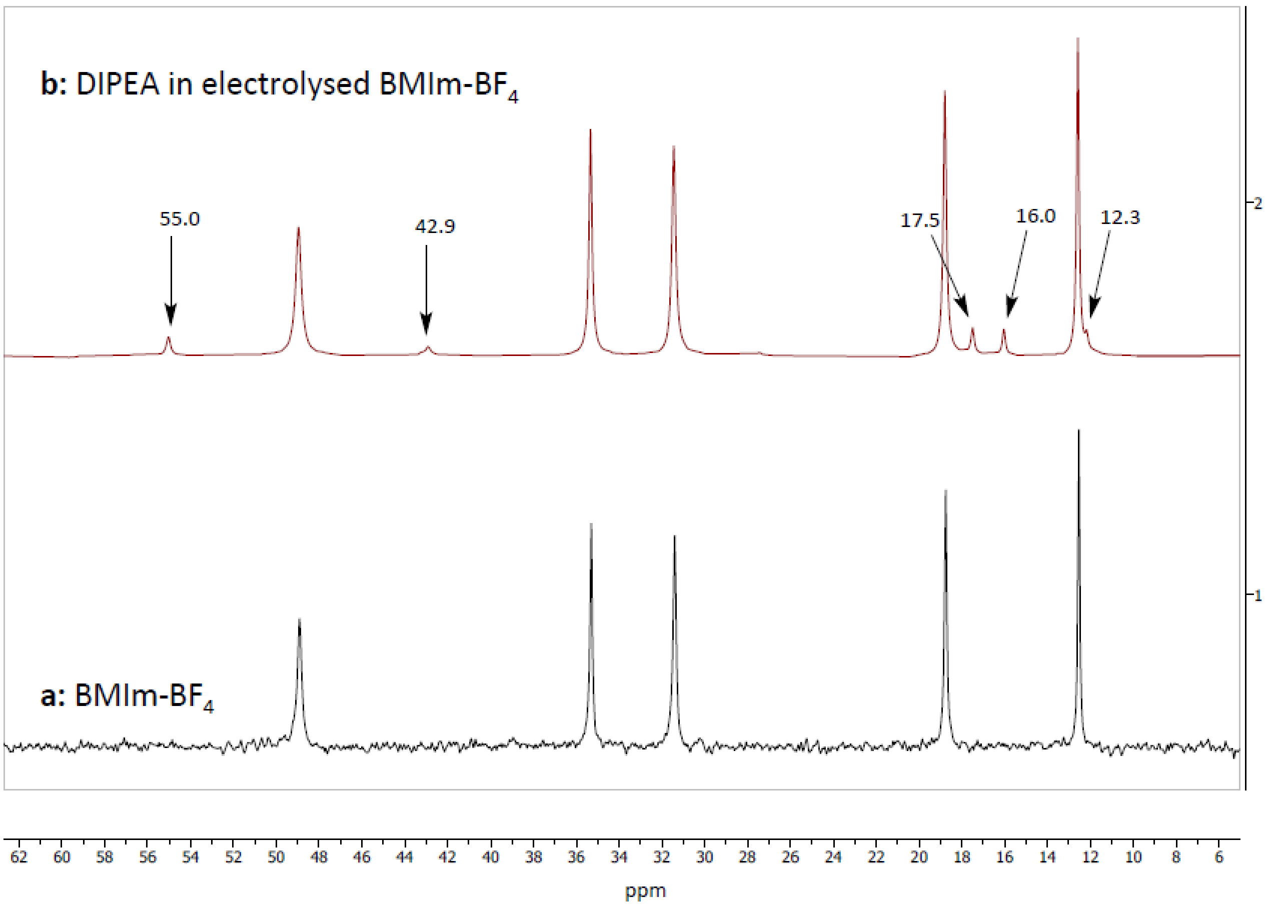

Figure S2. ^{13}C NMR spectra under neat conditions of (a) BMIm-BF_4, (b) DIPEA (0.6 mmol) added to anodically oxidated BMIm-BF_4 (60 C) in a divided cell.

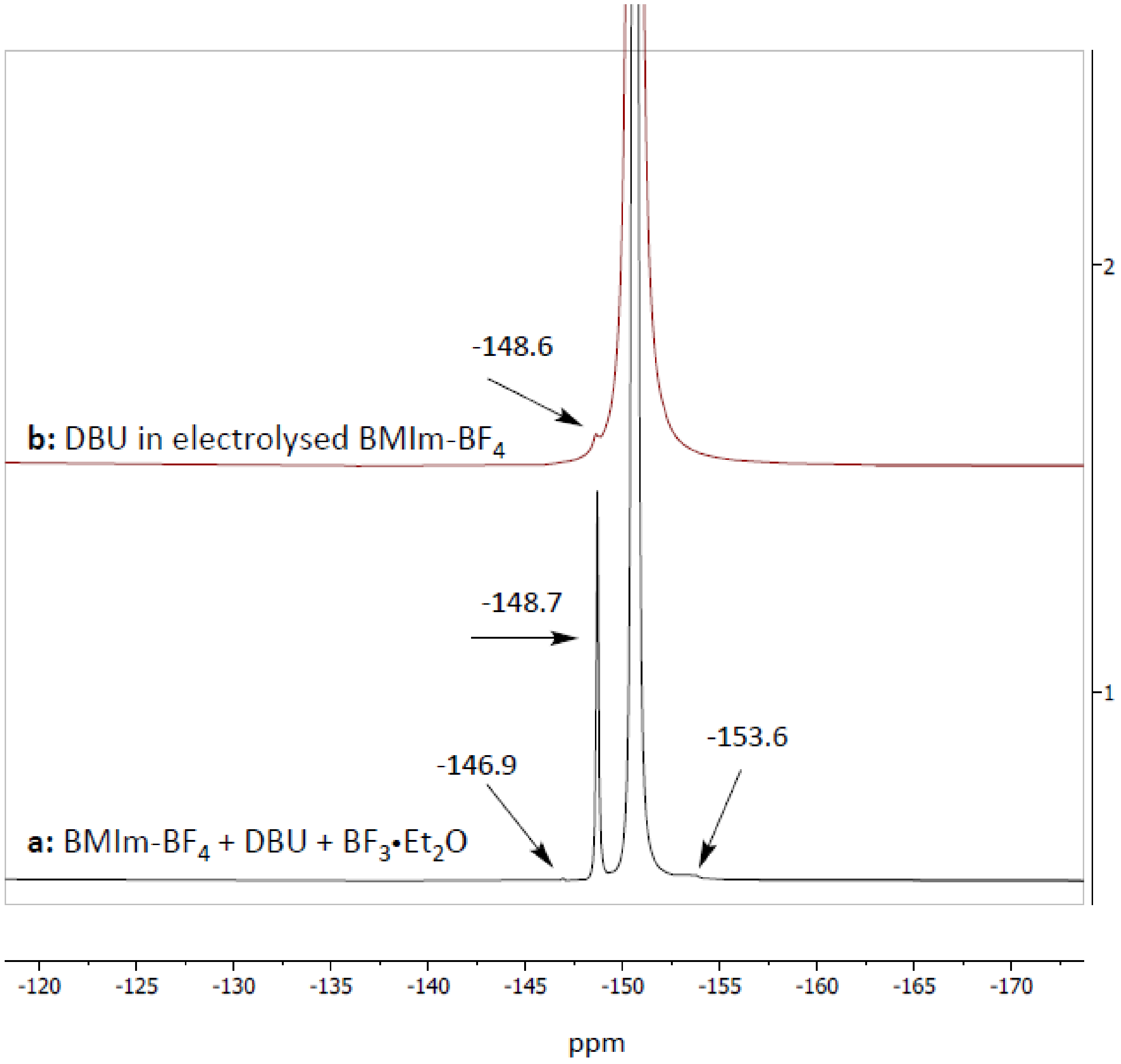

Figure S3. ^{19}F NMR spectra under neat conditions of (a) DBU and $BF_3{\cdot}Et_2O$ in BMIm-BF_4, (b) DBU (0.6 mmol) added to anodically oxidated BMIm-BF_4 (60 C) in a divided cell.

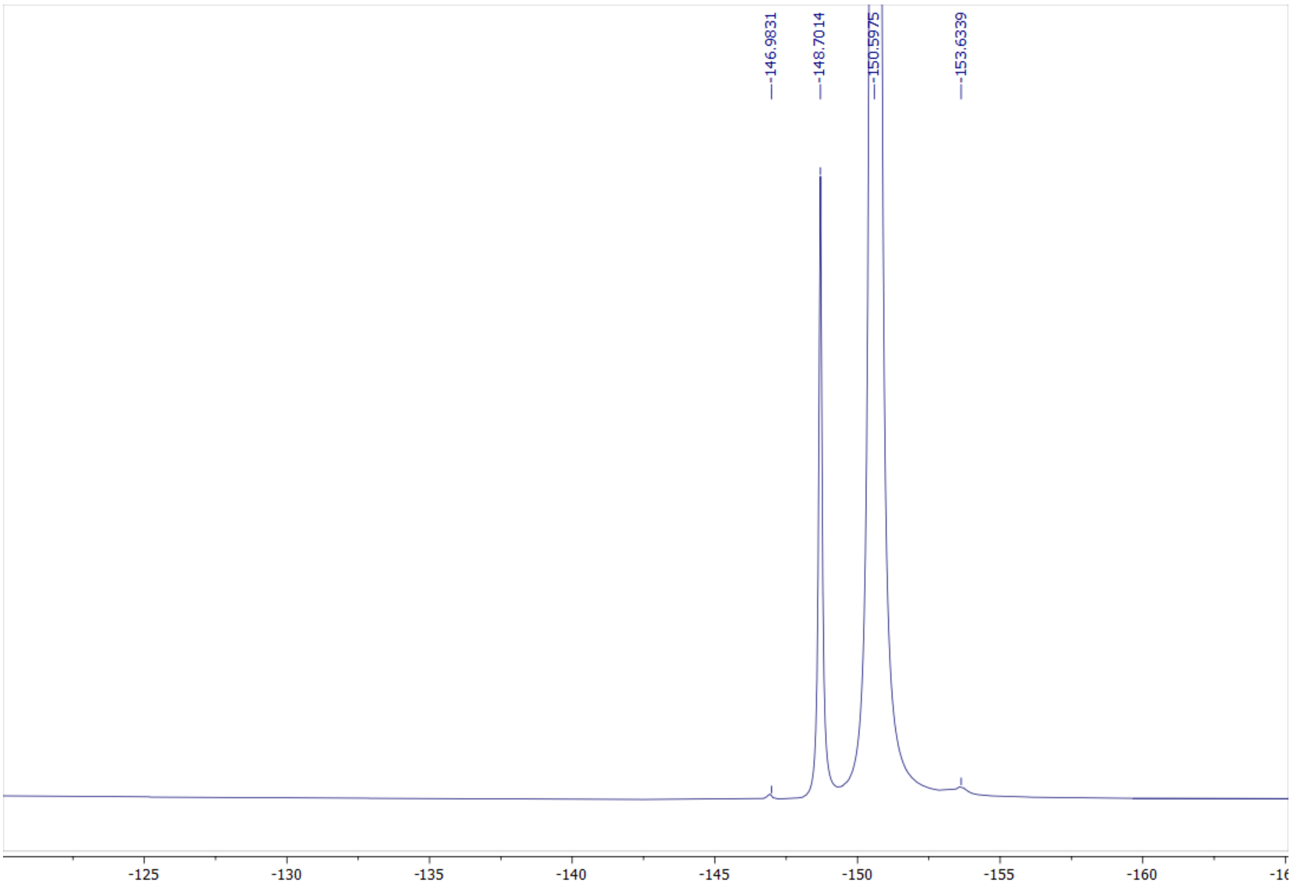

Figure S4. ^{19}F NMR spectra under neat conditions of DBU and $BF_3{\cdot}Et_2O$ in BMIm-BF_4.

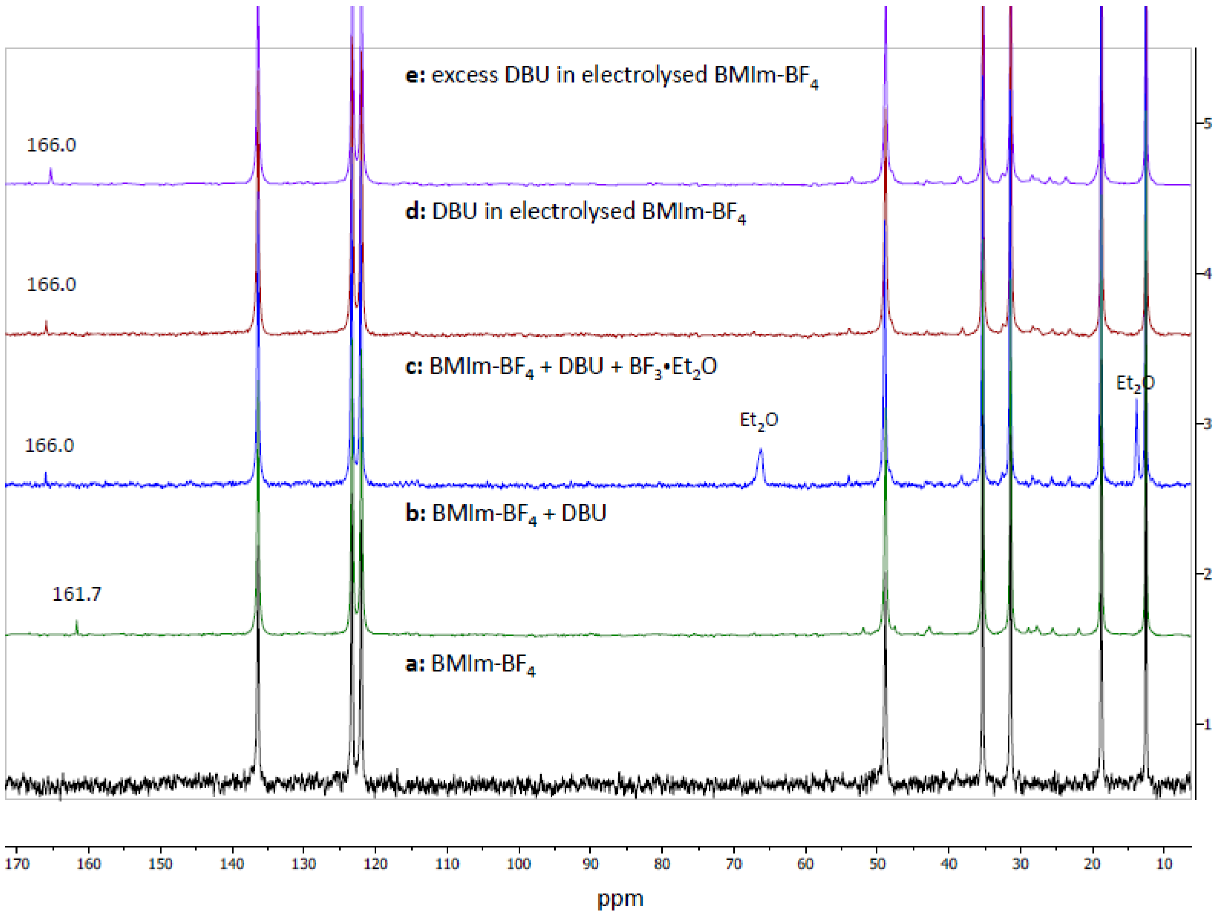

Figure S5. ^{13}C NMR spectra under neat conditions of (a) BMIm-BF_4, (b) DBU in BMIm-BF_4, (c) $BF_3{\cdot}Et_2O$ and DBU in BMIm-BF_4, (d) DBU (0.6 mmol) in anodically oxidated BMIm-BF_4 (60 C) in a divided cell, (e) DBU (1.2 mmol) in anodically oxidated BMIm-BF_4 (60 C) in a divided cell.

Analytical data

All isolated products were known and their spectral data were in accordance with those reported in the literature.

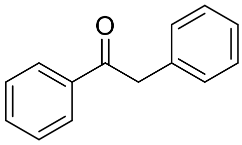

1,2-Diphenylethan-1-one (**2a**):

The product was isolated after flash chromatography on silica gel (light petroleum ether/EtOAc 95:5) as a white solid.[1]

^{1}H NMR (400 MHz, $CDCl_3$) δ 8.04-8.01 (m, 2H); 7.58-7.54 (m, 1H); 7.48-7.44 (m, 2H); 7.35-7.31 (m, 2H); 7.28-7.24 (m, 3H); 4.29 (s, 2H).

^{13}C{^{1}H} NMR (101 MHz, $CDCl_3$) δ 197.77; 136.75; 134.68; 133.30; 129.61; 128.81; 128.79; 128.76; 127.03; 45.64.

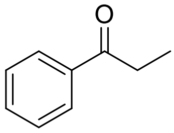

Propiophenone (**2b**):

The product was isolated after flash chromatography on silica gel (CH_2Cl_2/light petroleum ether 6:4) as a colourless oil.[2]

^{1}H NMR (400 MHz, $CDCl_3$) δ 7.98-7.96 (m, 2H); 7.57-7.53 (m, 1H); 7.48-7.44 (m, 2H); 3.01 (q, J = 7.24 Hz, 2H); 1.23 (t, J = 7.24 Hz, 3H).

^{13}C{^{1}H} NMR (101 MHz, $CDCl_3$) δ 200.99; 137.07; 133.01; 128.69; 128.11; 31.92; 8.38.

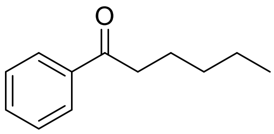

1-Phenylhexan-1-one (**2c**):

The product was isolated after flash chromatography on silica gel (light petroleum ether/EtOAc 97:3) as a colourless oil.[3]

^{1}H NMR (400 MHz, $CDCl_3$) δ 7.97-7.94 (m, 2H); 7.57-7.52 (m, 1H); 7.47-7.43 (m, 2H); 2.96 (t, J = 7.36 Hz, 2H); 1.78-1.70 (m, 2H); 1.40-1.34 (m, 4H); 0.93-0.89 (m, 3H).

$^{13}C\{^1H\}$ NMR (101 MHz, $CDCl_3$) δ 200.74; 137.21; 132.97; 128.66; 128.16; 38.70; 31.67; 24.18; 22.65; 14.08.

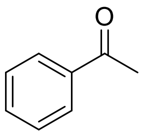

Acetophenone (**2d**):

The product was isolated after flash chromatography on silica gel (light petroleum ether/EtOAc 95:5) as a colourless oil.[2]

^1H NMR (400 MHz, $CDCl_3$) δ 7.94-7.92 (m, 2H); 7.54-7.51 (m, 1H); 7.44-7.41 (m, 2H); 2.56 (s, 3H).

$^{13}C\{^1H\}$ NMR (101 MHz, $CDCl_3$) δ 198.07; 137.17; 133.09; 128.57; 128.30; 26.55.

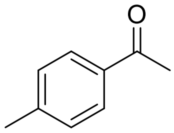

1-(p-Tolyl)ethan-1-one (**2e**):

The product was isolated after flash chromatography on silica gel (light petroleum ether/EtOAc 95:5) as a pale yellow oil.[2]

^1H NMR (400 MHz, $CDCl_3$) δ 7.85-7.82 (m, 2H); 7.24-7.22 (m, 2H); 2.55 (s, 3H); 2.38 (s, 3H).

$^{13}C\{^1H\}$ NMR (101 MHz, $CDCl_3$) δ 197.94; 143.92; 134.73; 129.27; 128.48; 26.53; 21.64.

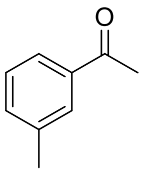

1-(m-Tolyl)ethan-1-one (**2f**):

The product was isolated after flash chromatography on silica gel (light petroleum ether/EtOAc 95:5) as a pale yellow oil.[2]

^1H NMR (400 MHz, $CDCl_3$) δ 7.77-7.74 (m, 2H); 7.38-7.32 (m, 2H); 2.59 (s, 3H); 2.41 (s, 3H).

$^{13}C\{^1H\}$ NMR (101 MHz, $CDCl_3$) δ 198.54; 138.46; 137.27; 133.98; 128.90; 128.55; 125.70; 26.79; 21.44.

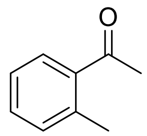

1-(o-Tolyl)ethan-1-one (**2g**):

The product was isolated after flash chromatography on silica gel (light petroleum ether/EtOAc 95:5) as a colourless oil.[4]

^{1}H NMR (400 MHz, $CDCl_3$) δ 7.60 (d, *J* = 7.80 Hz, 1H); 7.28 (td, J_1 = 7.48, J_2 = 1.16 Hz, 1H); 7.19-7.14 (m, 2H); 2.49 (s, 3H); 2.44 (s, 3H).

^{13}C{^{1}H} NMR (101 MHz, $CDCl_3$) δ 201.80; 138.49; 137.70; 132.12; 131.60; 129.45; 125.78; 29.61; 21.67.

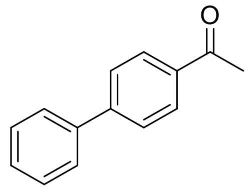

1-([1,1'-Biphenyl]-4-yl)ethan-1-one (**2h**):

The product was isolated after flash chromatography on silica gel (light petroleum ether/EtOAc 9:1) as a white solid.[5]

^{1}H NMR (400 MHz, $CDCl_3$) δ 8.05-8.02 (m, 2H); 7.71-7.68 (m, 2H); 7.65-7.62 (m, 2H); 7.50-7.45 (m, 2H); 7.43-7.38 (m, 1H); 2.64 (s, 3H).

^{13}C{^{1}H} NMR (101 MHz, $CDCl_3$) δ 197.89; 145.90; 139.98; 135.96; 129.08; 129.04; 128.36; 127.39; 127.34; 26.79.

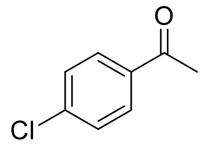

1-(4-Chlorophenyl)ethan-1-one (**2i**):

The product was isolated after flash chromatography on silica gel (light petroleum ether/EtOAc 95:5) as a colourless oil.[4]

^{1}H NMR (400 MHz, $CDCl_3$) δ 7.88 (d, *J* = 8.56 Hz, 2H); 7.41 (d, *J* = 8.52 Hz, 2H); 2.57 (s, 3H).

^{13}C{^{1}H} NMR (101 MHz, $CDCl_3$) δ 196.90; 139.64; 135.51; 129.82; 128.97; 26.65.

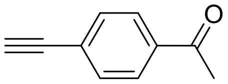

1-(4-Ethynylphenyl)ethan-1-one (**2j**):

The product was isolated after flash chromatography on silica gel (light petroleum ether/EtOAc 8:2) as a yellow solid.[6]

^{1}H NMR (400 MHz, $CDCl_3$) δ 7.89 (d, *J* = 8.40 Hz, 2H); 7.55 (d, *J* = 8.40 Hz, 2H); 3.25 (s, 1H); 2.58 (s, 3H).

^{13}C{^{1}H} NMR (101 MHz, $CDCl_3$) δ 197.37; 136.84; 132.37; 128.28; 127.00; 82.84; 80.51; 26.72.

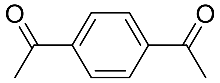

1,1'-(1,4-Phenylene)bis(ethan-1-one) (**2jj**):

The product was isolated after flash chromatography on silica gel (light petroleum ether/EtOAc 8:2) as a pinkish solid.[7]

^{1}H NMR (400 MHz, $CDCl_3$) δ 8.02 (s, 4H); 2.63 (s, 6H).

^{13}C{^{1}H} NMR (101 MHz, $CDCl_3$) δ 197.61; 140.28; 128.61; 27.01.

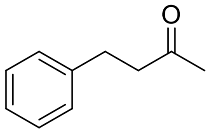

4-Phenylbutan-2-one (**2k**):

The product was isolated after flash chromatography on silica gel (light petroleum ether/EtOAc 95:5) as a colourless oil.[4]

^{1}H NMR (400 MHz, $CDCl_3$) δ 7.30-7.28 (m, 2H); 7.21-7.17 (m, 3H); 2.90 (t, *J* = 7.40 Hz, 2H); 2.76 (t, *J* = 7.40 Hz, 2H); 2.14 (s, 3H).

^{13}C{^{1}H} NMR (101 MHz, $CDCl_3$) δ 208.15; 141.13; 128.65; 128.44; 126.26; 45.34; 30.24; 29.88.

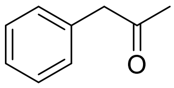

1-Phenylpropan-2-one (**2l**):

The product was isolated after flash chromatography on silica gel (light petroleum ether/EtOAc 9:1) as a pale yellow oil.[4]

^{1}H NMR (400 MHz, $CDCl_3$) δ 7.34-7.30 (m, 2H); 7.27-7.24 (m, 1H); 7.20-7.18 (m, 2H); 3.68 (s, 2H); 2.14 (s, 3H).

$^{13}C\{^1H\}$ NMR (101 MHz, $CDCl_3$) δ 206.61; 134.37;129.53; 128.90; 127.21; 51.18; 29.41.

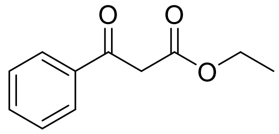

Ethyl 3-oxo-3-phenylpropanoate (**2m**):

The product was isolated after flash chromatography on silica gel (light petroleum ether/EtOAc 9:1) as a mixture of ketone and enol forms (5:1), as a yellow oil.[8]

Ketone: ^1H NMR (400 MHz, $CDCl_3$) δ 7.95-7.93 (m, 2H); 7.61-7.57 (m, 1H); 7.49-7.46 (m, 2H); 4.21 (q, J = 7.16 Hz, 2H); 3.99 (s, 2H); 1.25 (t, J = 7.12 Hz, 3H).

$^{13}C\{^1H\}$ NMR (101 MHz, $CDCl_3$) δ 192.65; 167.63; 131.34; 128.88; 128.61; 126.15; 61.58; 46.11; 14.17.

Enol: ^1H NMR (400 MHz, $CDCl_3$) δ 12.58 (s, 1H); 7.78-7.76 (m, 2H); 7.44-7.39 (m, 3H); 5.66 (s, 1H); 4.26 (q, J = 7.16 Hz, 2H); 1.33 (t, J = 7.12 Hz, 3H).

$^{13}C\{^1H\}$ NMR (101 MHz, $CDCl_3$) δ 173.31; 171.54; 136.14; 133.84; 133.56; 128.64; 87.50; 60.43; 14.41.

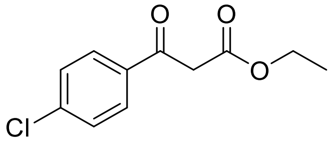

Ethyl 3-(4-chlorophenyl)-3-oxopropanoate (**2n**):

The product was isolated after flash chromatography on silica gel (CH_2Cl_2/light petroleum ether 1:1) as a mixture of ketone and enol forms (3.8:1), as a yellow oil.[9]

Ketone: ^1H NMR (400 MHz, $CDCl_3$) δ 7.87 (d, J = 8.60 Hz, 2H); 7.45 (d, J = 8.56 Hz, 2H); 4.20 (q, J = 7.16 Hz, 2H); 3.95 (s, 2H); 1.25 (t, J = 7.16 Hz, 3H).

$^{13}C\{^1H\}$ NMR (101 MHz, $CDCl_3$) δ 191.45; 167.34; 140.43; 134.46; 130.06; 129.25; 61.74; 46.08; 14.18.

Enol: ^1H NMR (400 MHz, $CDCl_3$) δ 12.57 (s, 1H); 7.69 (d, J = 8.64 Hz, 2H); 7.38 (d, J = 8.60, 2H); 5.63 (s, 1H); 4.26 (q, J = 7.12 Hz, 2H); 1.33 (t, J = 7.12 Hz, 3H).

$^{13}C\{^1H\}$ NMR (101 MHz, $CDCl_3$) δ 173.18; 170.25; 137.42; 132.02; 128.94; 127.48; 87.79; 60.59; 14.40.

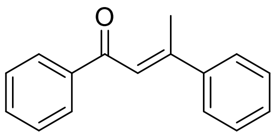

(E)-1,3-Diphenylbut-2-en-1-one (**3d**):

The product was isolated after flash chromatography on silica gel (light petroleum ether/EtOAc 95:5) as a pale yellow oil.[10]

^{1}H NMR (400 MHz, $CDCl_3$) δ 8.02-7.99 (m, 2H); 7.59-7.54 (m, 3H); 7.50-7.40 (m, 5H); 7.17 (q, *J* = 1.28 Hz, 1H); 2.60 (d, *J* = 1.28 Hz, 3H).

^{13}C{^{1}H} NMR (101 MHz, $CDCl_3$) δ 192.04; 155.25; 142.93; 139.50; 132.69; 129.27; 128.75; 128.69; 128.43; 126.64; 122.27; 19.03.

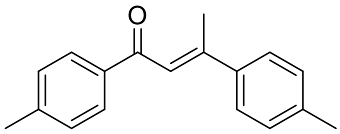

(E)-1,3-Di-p-tolylbut-2-en-1-one (**3e**):

The product was isolated after flash chromatography on silica gel (light petroleum ether/EtOAc 98:2) as a pale yellow solid.[10]

^{1}H NMR (400 MHz, $CDCl_3$) δ 7.92-7.89 (m, 2H); 7.50-7.47 (m, 2H); 7.28-7.26 (m, 2H); 7.24-7.22 (m, 2H); 7.16 (q, *J* = 1.28 Hz, 1H); 2.58 (d, *J* = 1.24 Hz, 3H); 2.42 (s, 3H); 2.40 (s, 3H).

^{13}C{^{1}H} NMR (101 MHz, $CDCl_3$) δ 191.75; 154.64; 143.32; 140.04; 139.37; 137.09; 129.42; 129.34; 128.54; 126.54; 121.61; 21.76; 21.36; 18.87.

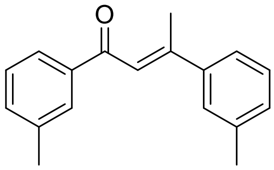

(E)-1,3-di-m-tolylbut-2-en-1-one (**3f**):

The product was isolated after flash chromatography on silica gel (light petroleum ether/EtOAc 95:5) as a pale yellow oil.[10]

^{1}H NMR (400 MHz, $CDCl_3$) δ 7.80-7.78 (m, 2H); 7.38-7.29 (m, 5H); 7.23-7.21 (m, 1H); 7.14 (q, *J* = 1.24 Hz, 1H); 2.58 (d, *J* = 1.28 Hz, 3H); 2.43 (s, 3H); 2.42 (s, 3H).

^{13}C{^{1}H} NMR (101 MHz, $CDCl_3$) δ 192.32; 155.22; 143.02; 139.59; 138.47; 138.38; 133.43; 129.99; 128.96; 128.64; 128.55; 127.34; 125.64; 123.78; 122.33; 21.66; 21.55; 19.08.

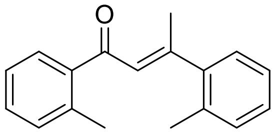

1,3-Di-o-tolylbut-2-en-1-one (*E*/*Z* mixture 1/0.25) (**3g**):

The product was isolated after flash chromatography on silica gel (light petroleum ether/EtOAc 95:5).[10]

^{1}H NMR (400 MHz, $CDCl_3$) δ 7.57 (d, *J* = 7.76 Hz, 1H *E* isomer); 7.48-7.45 (m, 1H *Z* isomer); 7.36-7.33 (m, 1H *E* isomer); 7.26-7.06 (m, 6H *E* isomer + 6H *Z* isomer); 6.94-6.92 (m, 1H, *Z* isomer); 6.64 (d, *J* = 1.32 Hz, 1H *Z* isomer); 6.53 (d, *J* = 1.36 Hz, 1H *E* isomer); 2.54 (s, 3H *E* isomer); 2.45 (d, *J* = 1.32 Hz, 3H *E* isomer); 2.36 (s, 3H *E* isomer); 2.32 (s, 3H *Z* isomer); 2.22 (s, 3H *Z* isomer); 2.19-2.17 (m, 3H, *Z* isomer).

^{13}C{^{1}H} NMR (101 MHz, $CDCl_3$) δ 196.00; 157.38; 144.41; 137.58; 134.11; 131.74; 131.31; 130.93; 130.65; 130.00; 128.60; 127.90; 127.74; 127.57; 127.41; 127.30; 126.63; 125.95; 125.77; 125.64; 125.22; 27.15; 21.62; 20.84; 20.52; 20.08; 19.54.

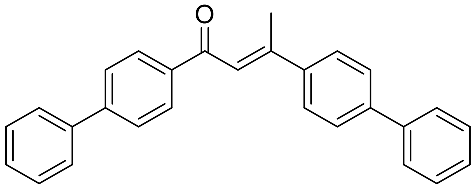

(E)-1,3-di([1,1'-biphenyl]-4-yl)but-2-en-1-one (**3h**):

The product was isolated after flash chromatography on silica gel (CH_2Cl_2/light petroleum ether 7:3) as a yellow solid.[11]

^{1}H NMR (400 MHz, $CDCl_3$) δ 8.11-8.09 (m, 2H); 7.73-7.63 (m, 10H); 7.50-7.37 (m, 6H); 7.29 (d, *J* = 1.24 Hz, 1H); 2.66 (d, *J* = 1.16 Hz, 3H).

^{13}C{^{1}H} NMR (101 MHz, $CDCl_3$) δ 190.51; 153.69; 144.42; 141.20; 140.69; 139.45; 139.18; 137.27; 128.10; 128.05; 127.30; 126.86; 126.44; 126.39; 126.22; 126.15; 121.06; 17.94.

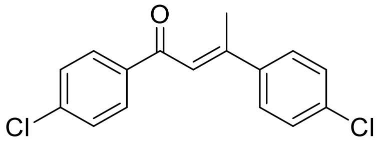

(E)-1,3-Bis(4-chlorophenyl)but-2-en-1-one (**3i**):

The product was isolated after flash chromatography on silica gel (light petroleum ether/EtOAc 95:5) as a pale yellow solid.[10]

^{1}H NMR (400 MHz, $CDCl_3$) δ 7.92 (d, *J* = 8.56 Hz, 2H); 7.50 (d, *J* = 8.60 Hz, 2H); 7.45 (d, *J* = 8.52 Hz, 2H); 7.39 (d, *J* = 8.60 Hz, 2H); 7.08 (d, *J* = 1.12 Hz, 1H); 2.56 (d, *J* = 1.08 Hz, 3H).
$^{13}C\{^{1}H\}$ NMR (101 MHz, $CDCl_3$) δ 190.46; 154.53; 141.08; 139.22; 137.62; 135.45; 129.84; 129.04; 129.01; 127.94; 121.92; 18.99.

^{1}H and $^{13}C\{^1H\}$ NMR spectra in $CDCl_3$

1,2-Diphenylethan-1-one (2a)

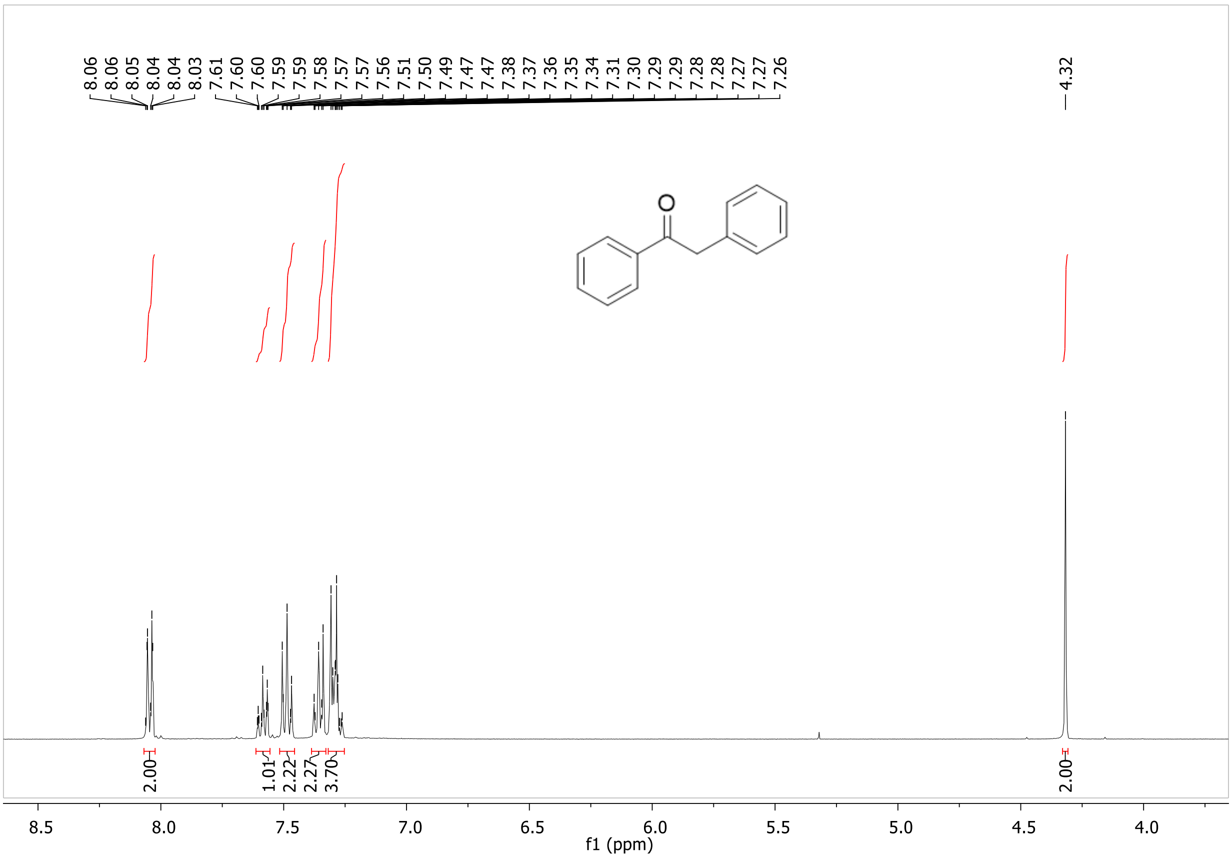

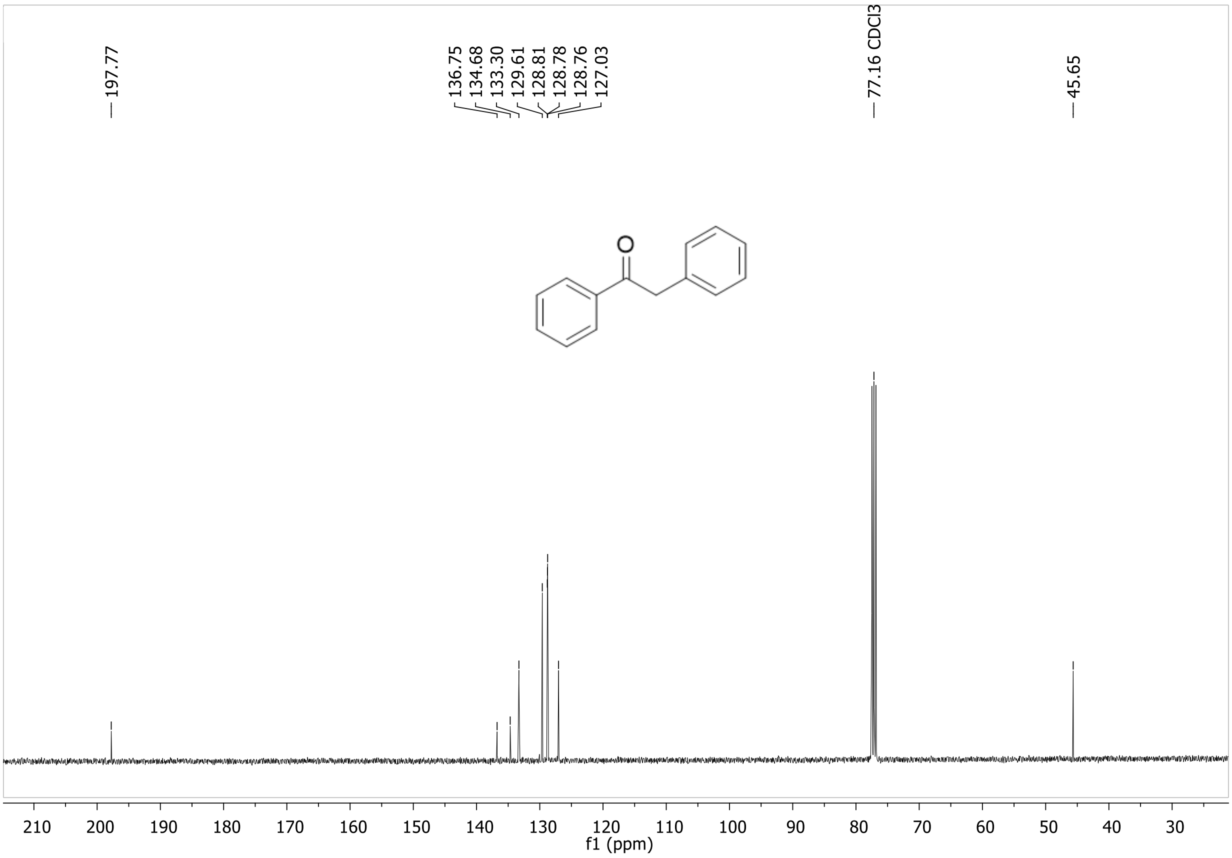

Propiophenone (2b)

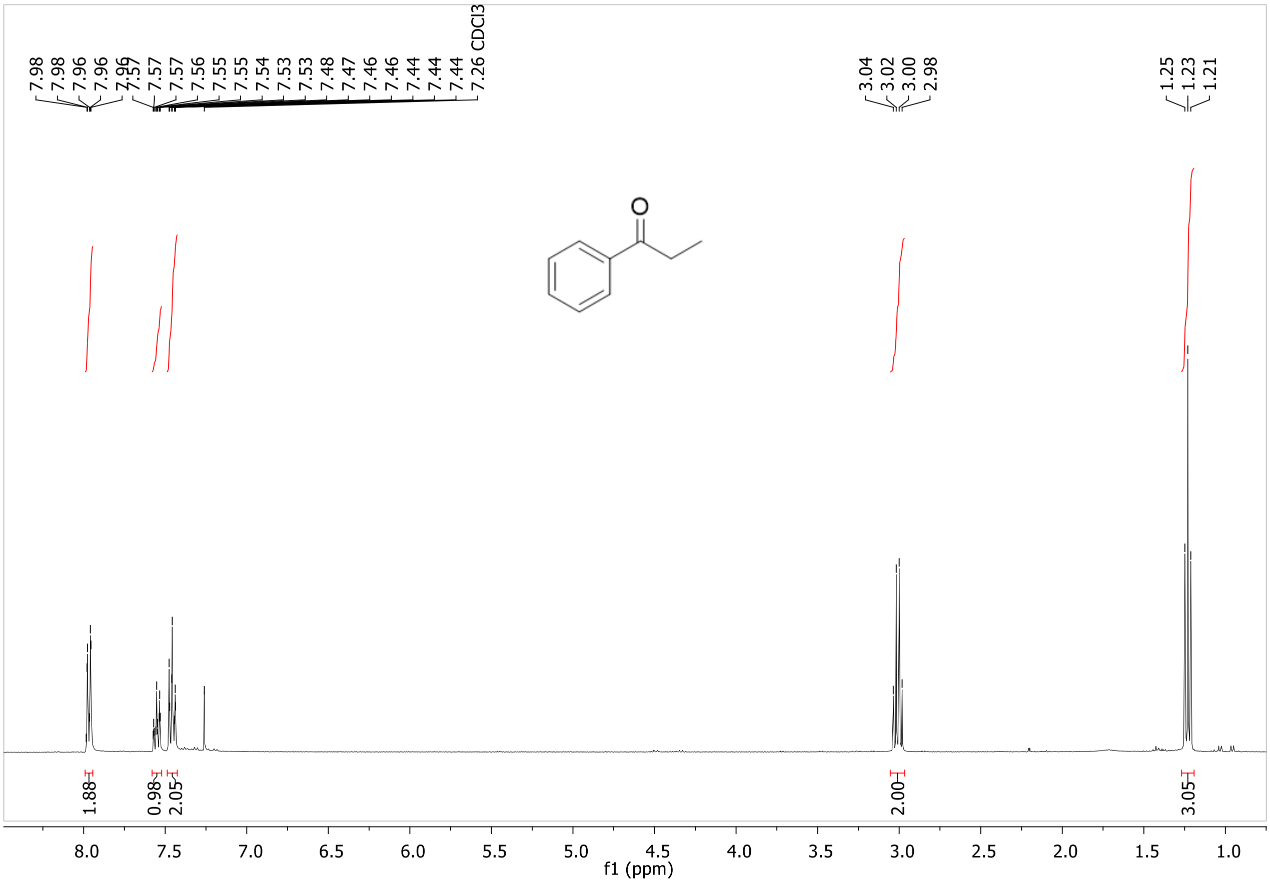

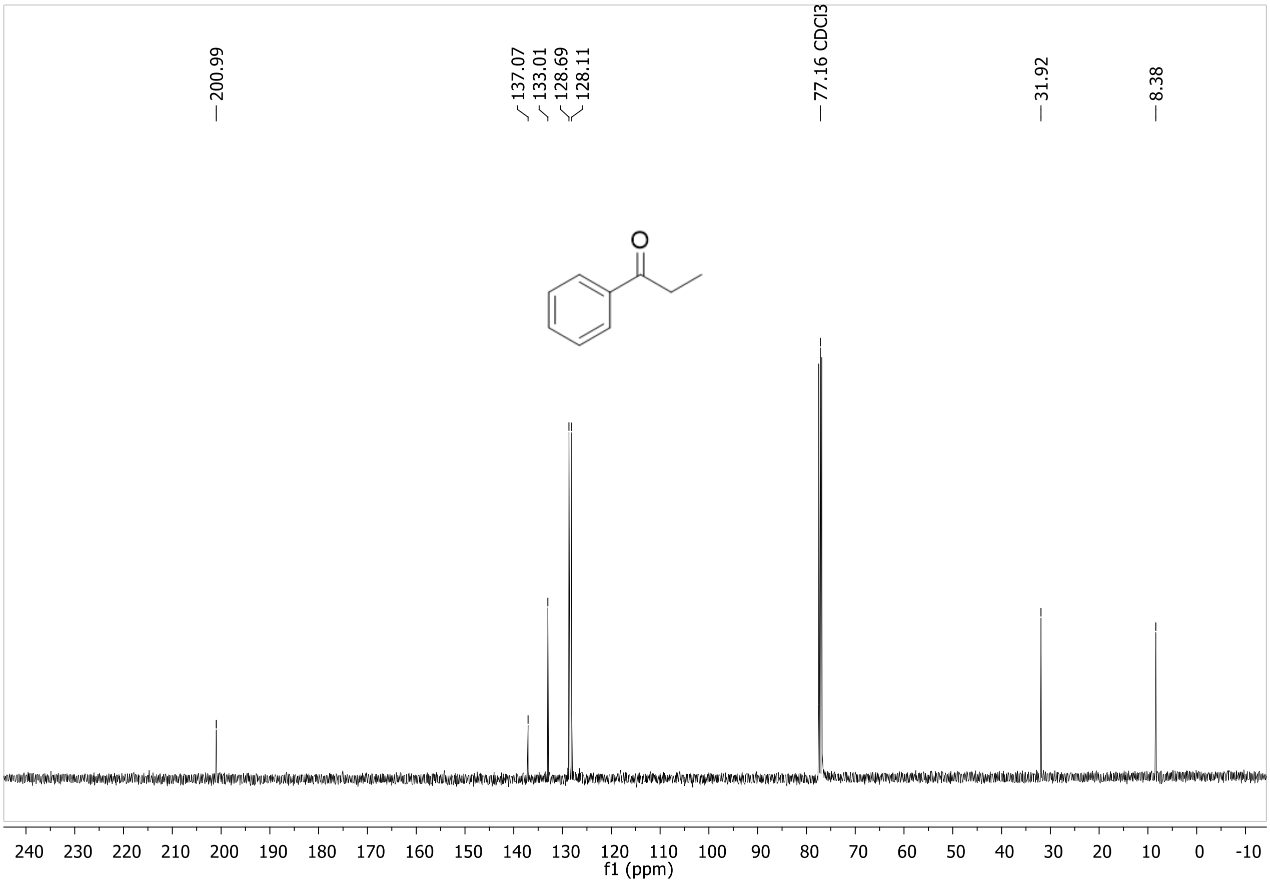

1-Phenylhexan-1-one (2c)

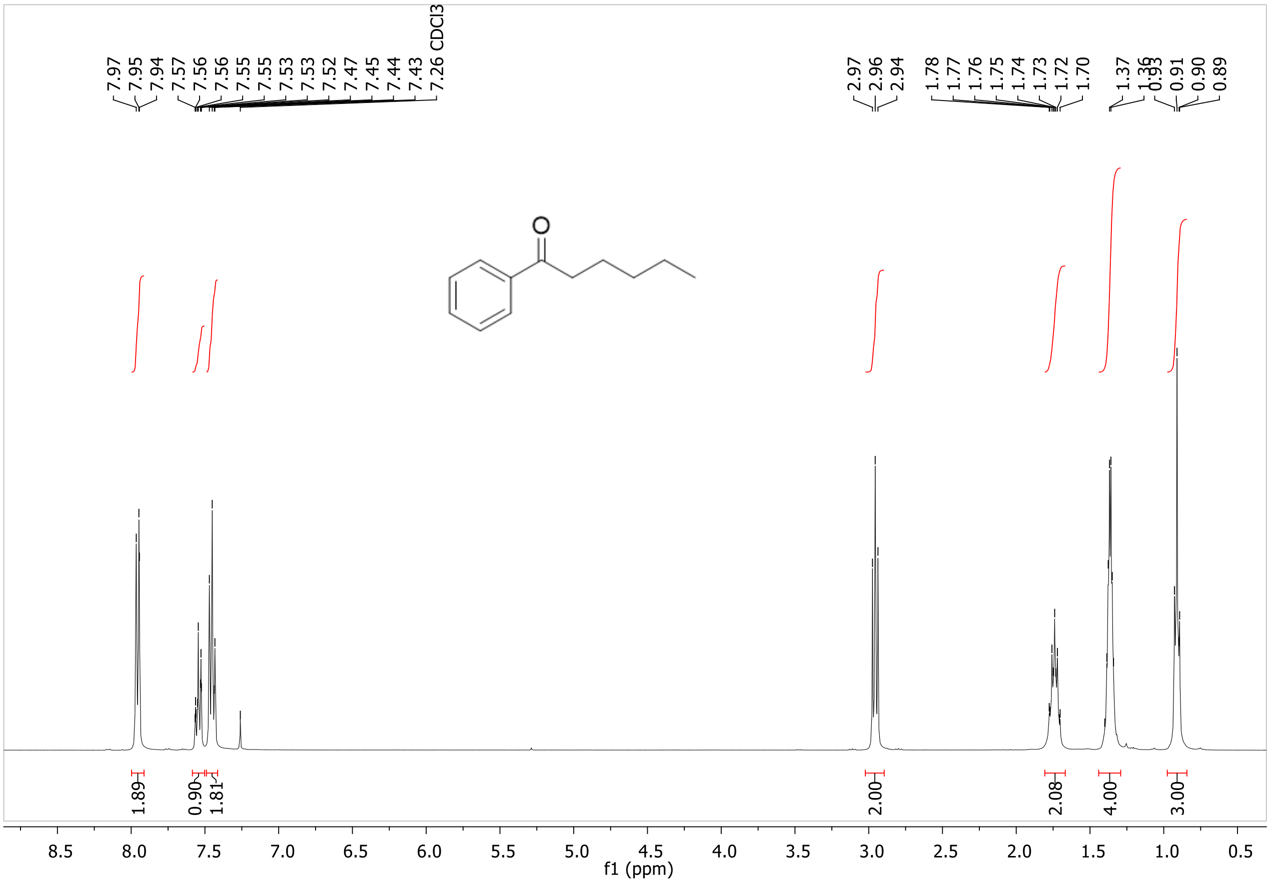

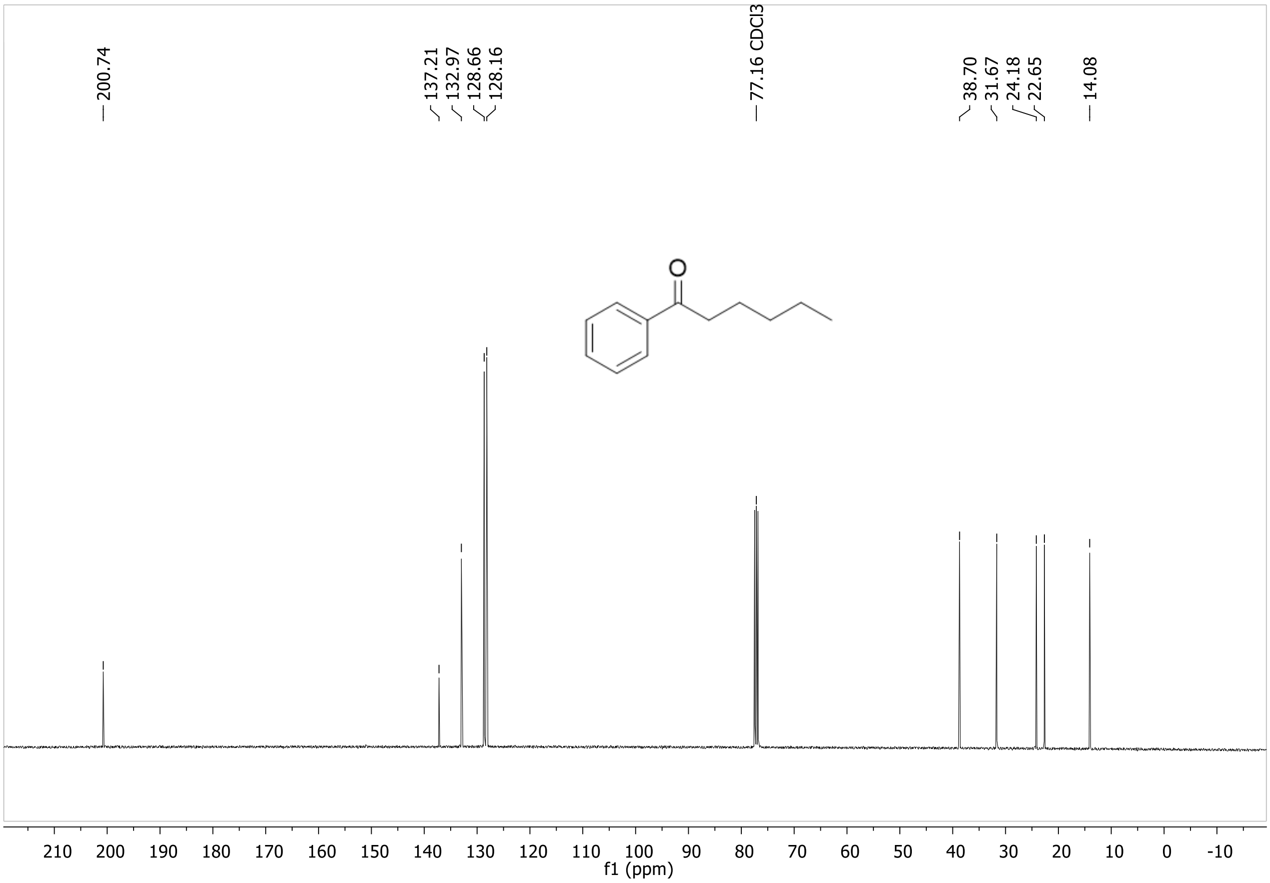

Acetophenone (2d)

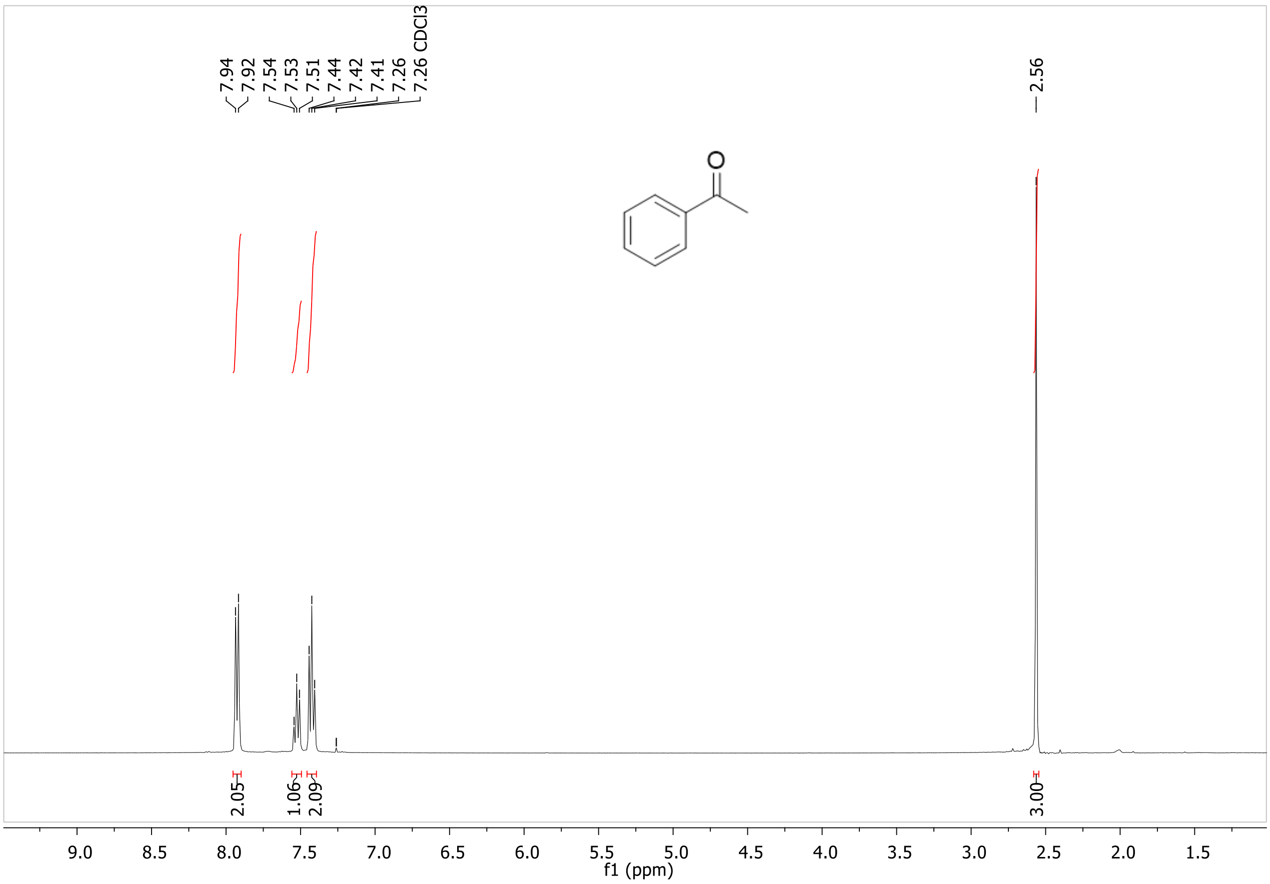

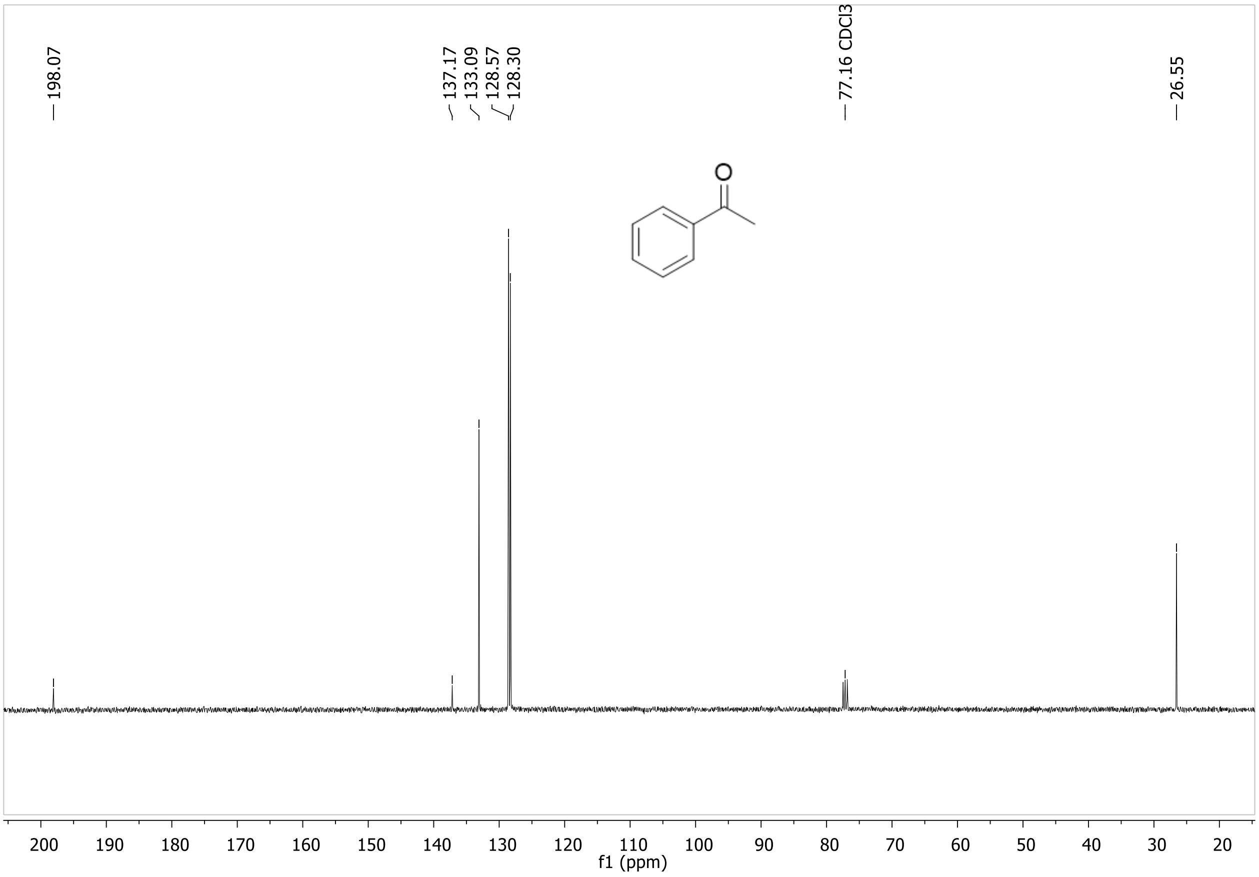

1-(*p*-Tolyl)ethan-1-one (2e)

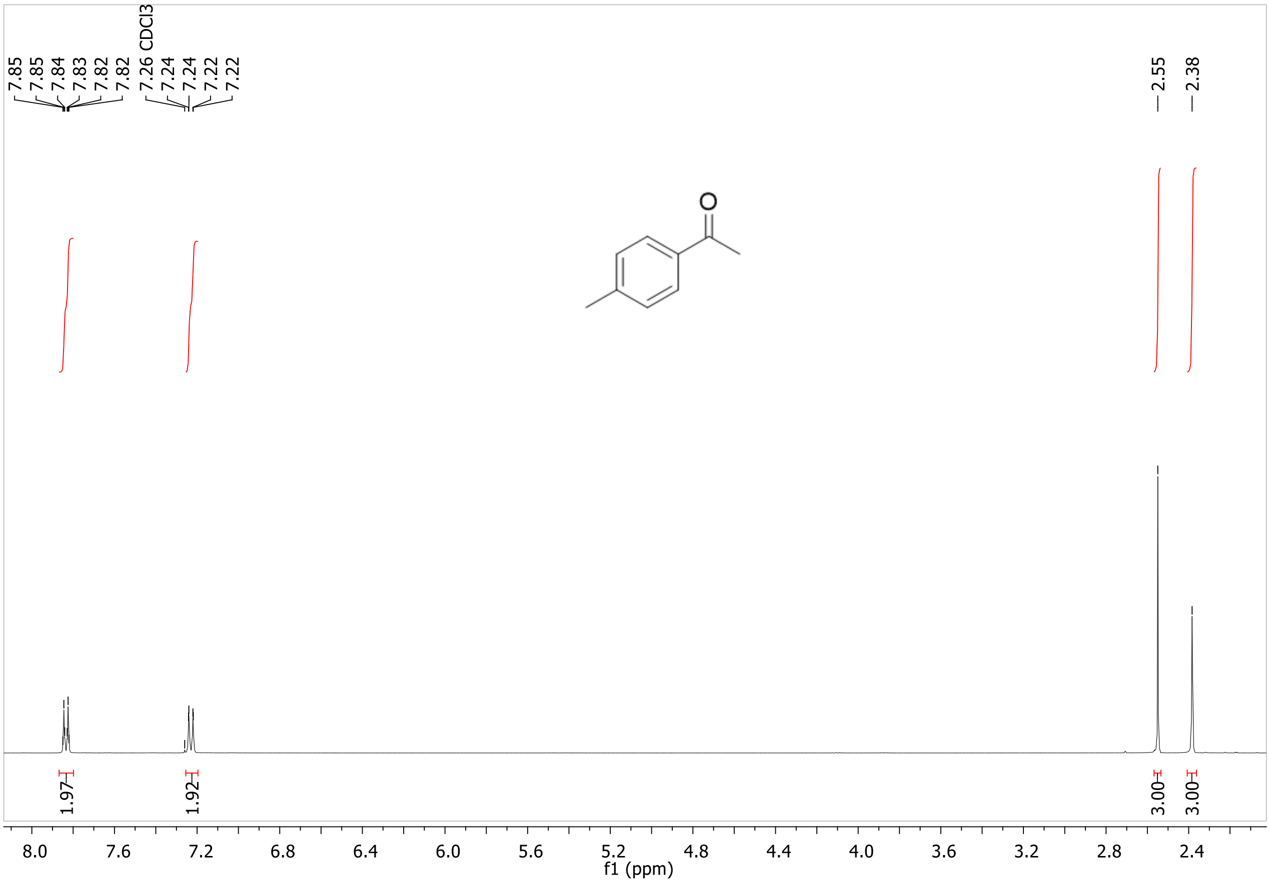

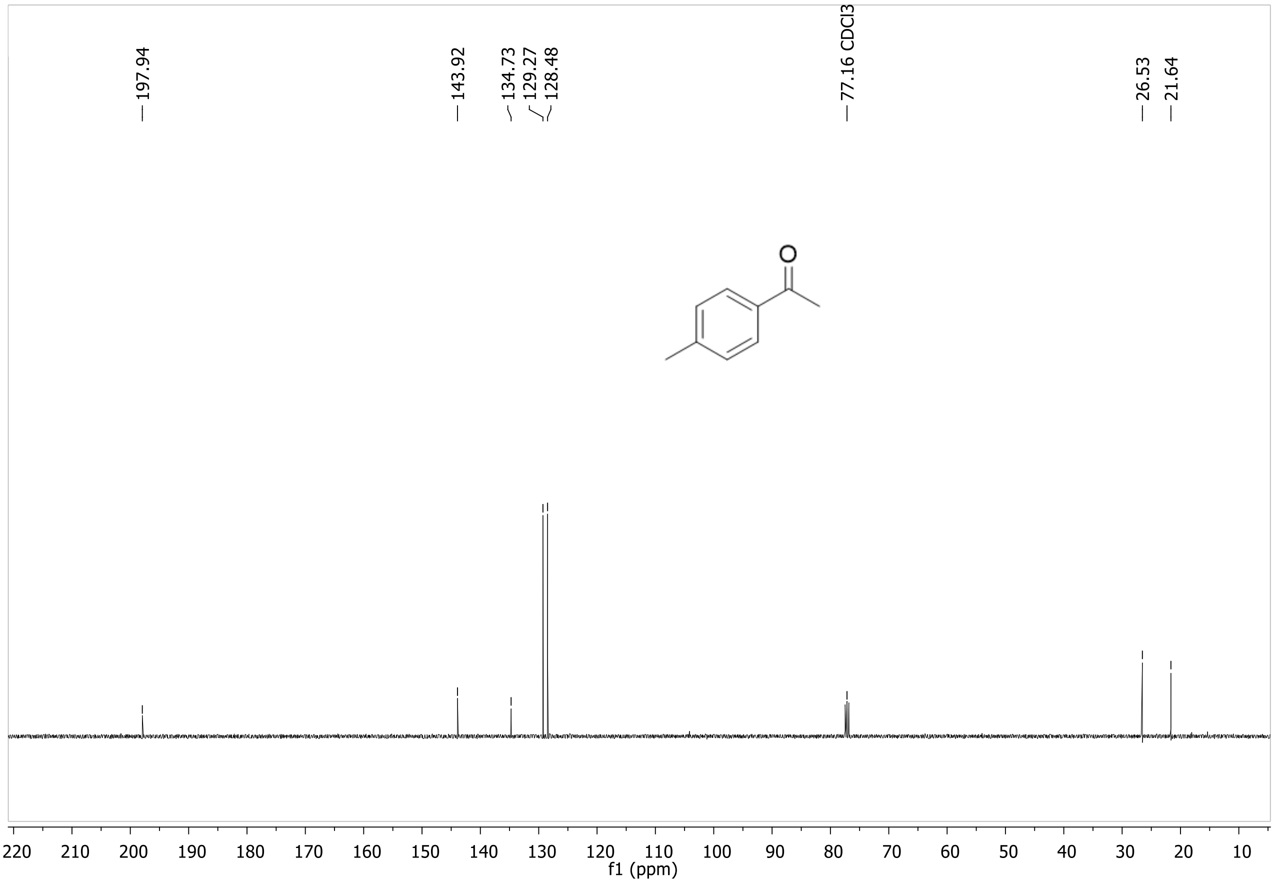

1-(*m*-Tolyl)ethan-1-one (2f)

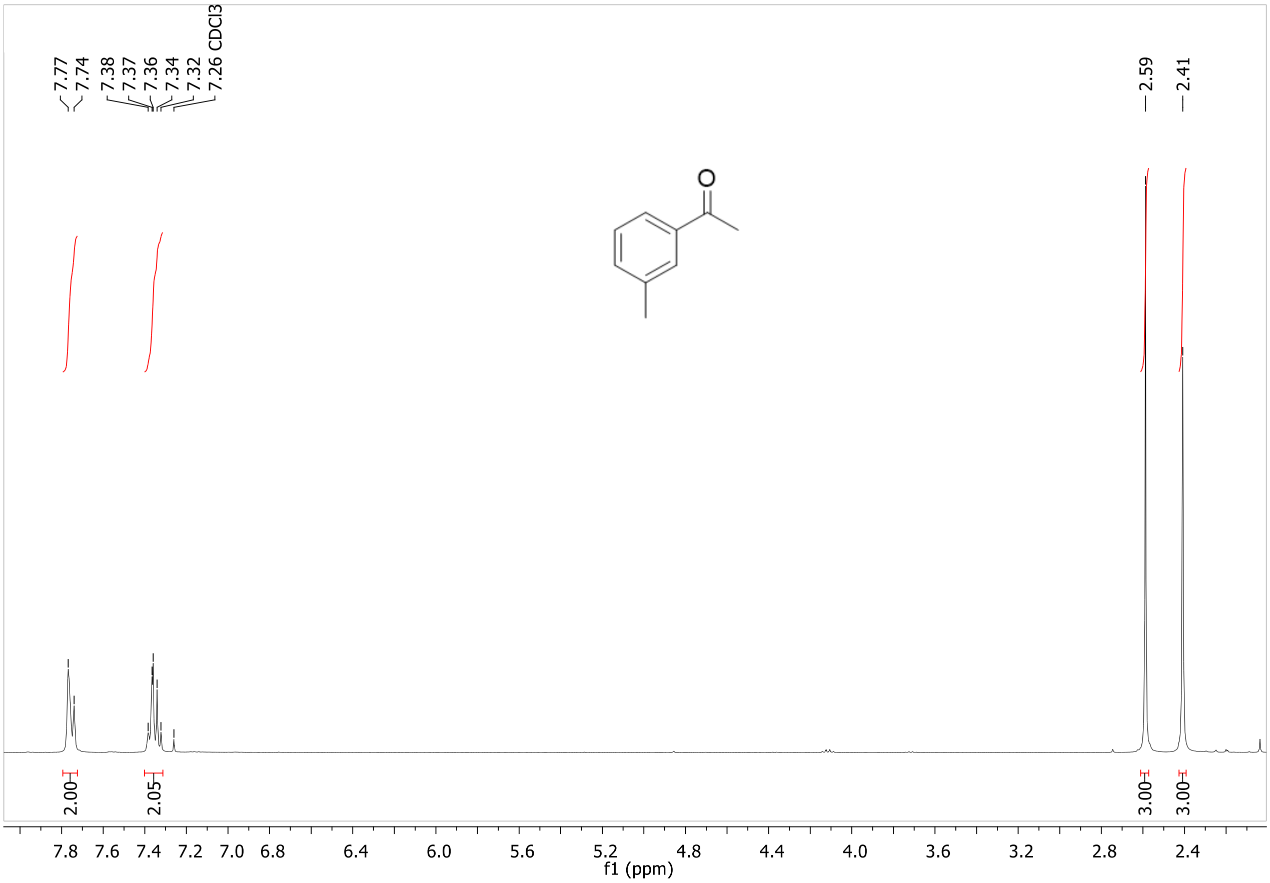

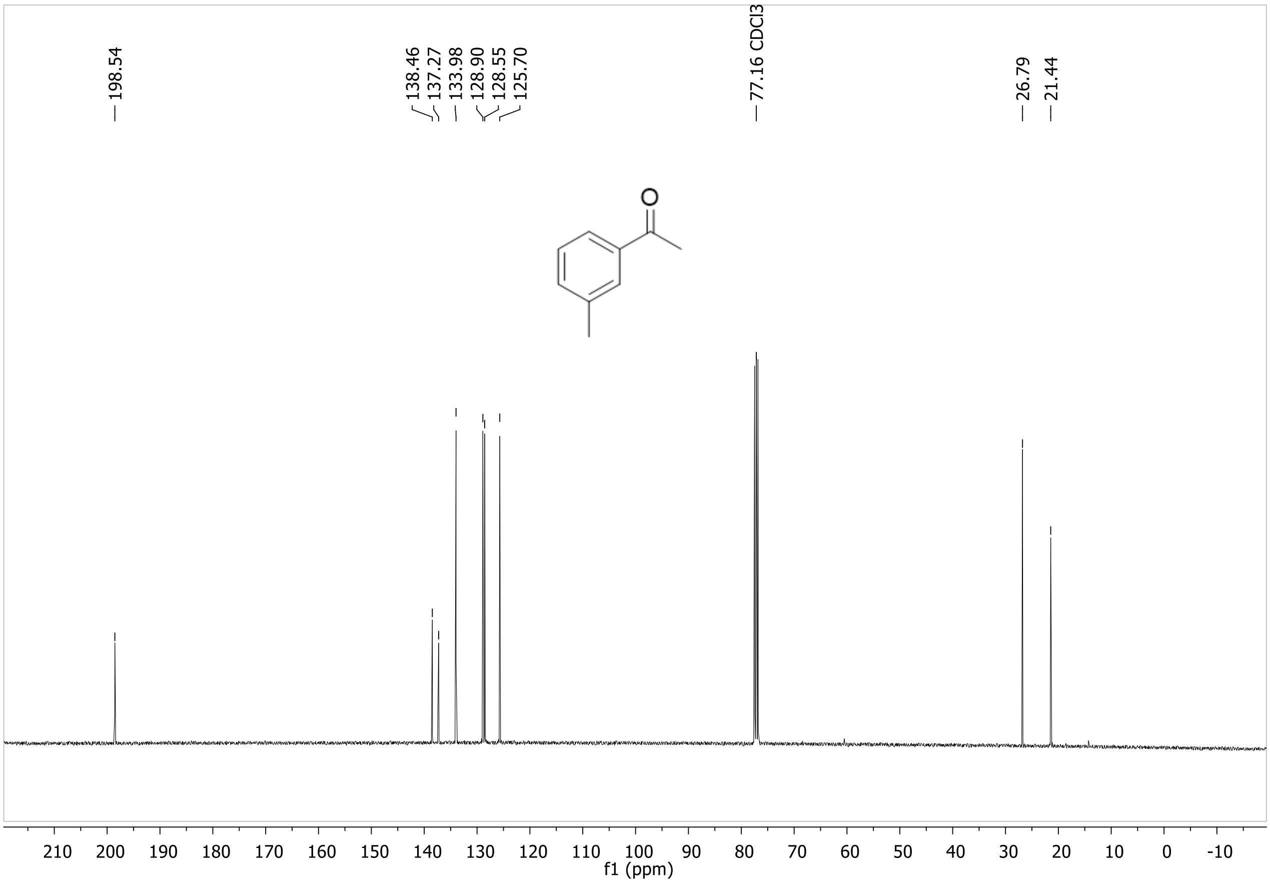

1-(*o*-Tolyl)ethan-1-one (2g)

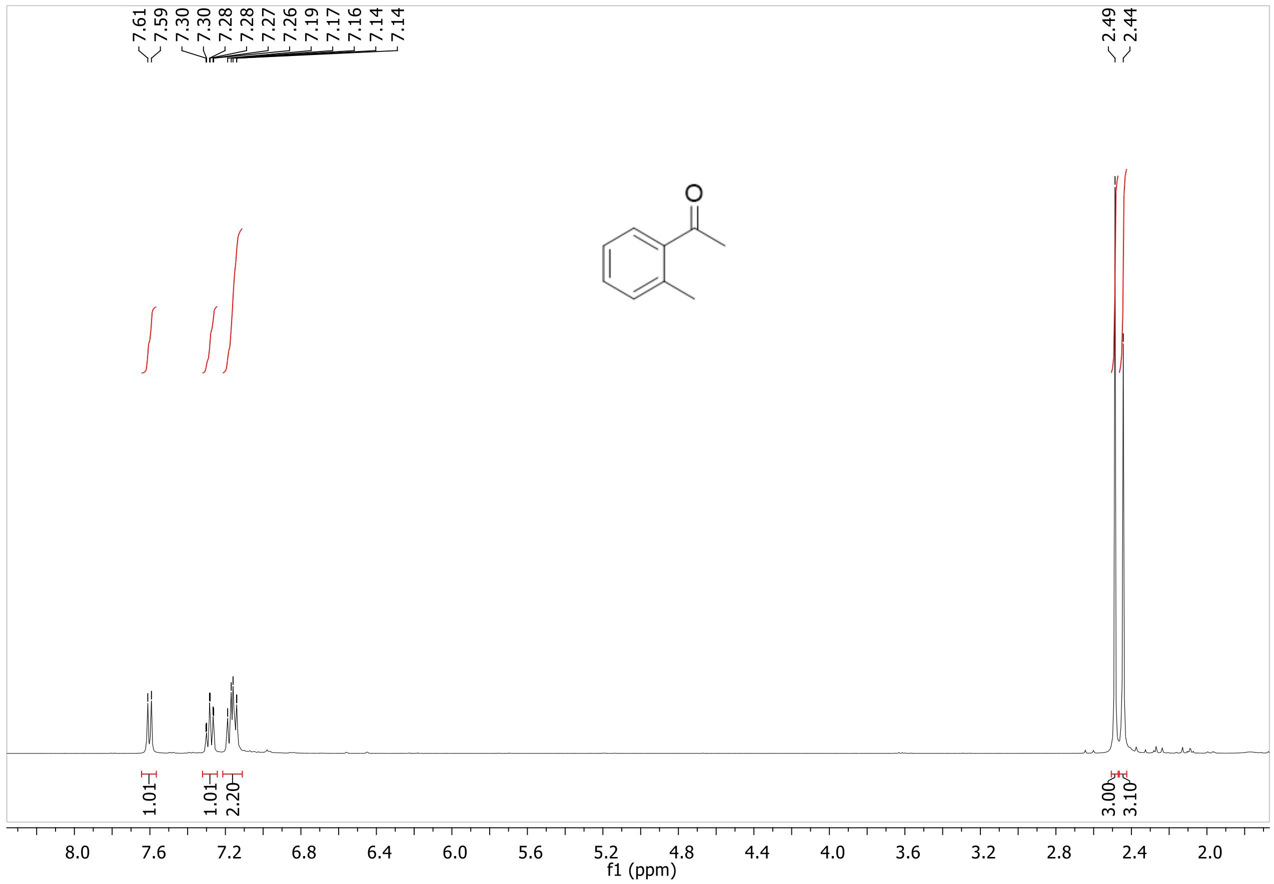

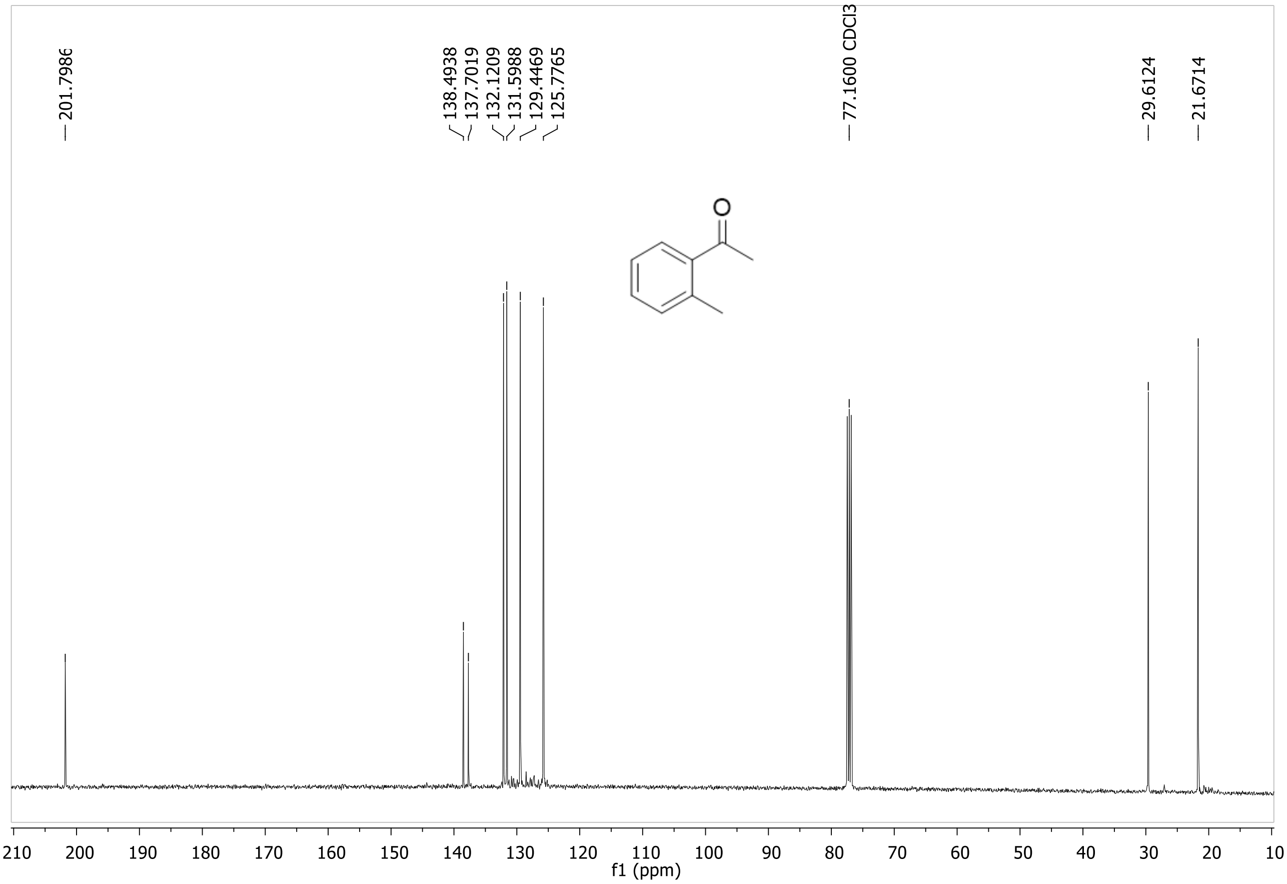

1-([1,1'-Biphenyl]-4-yl)ethan-1-one (2h)

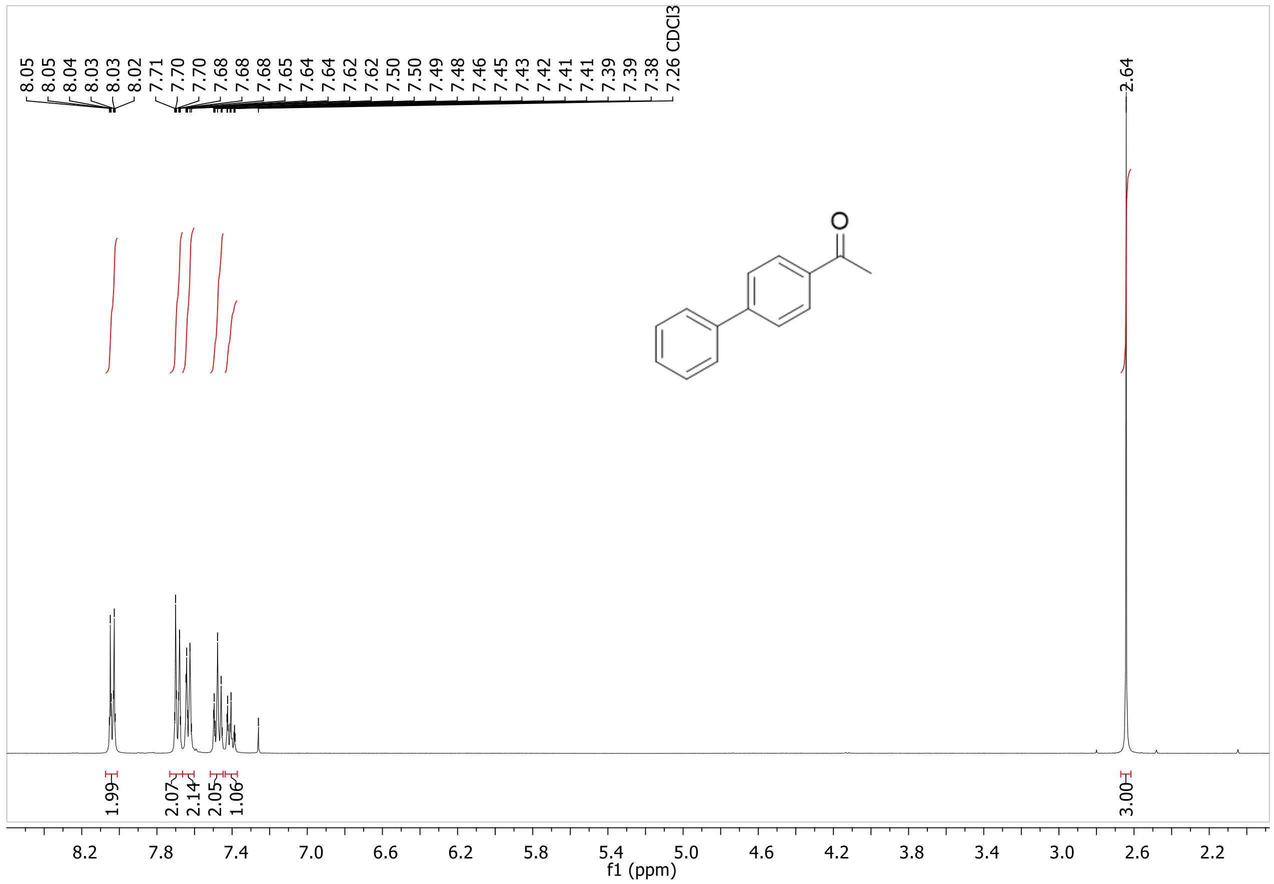

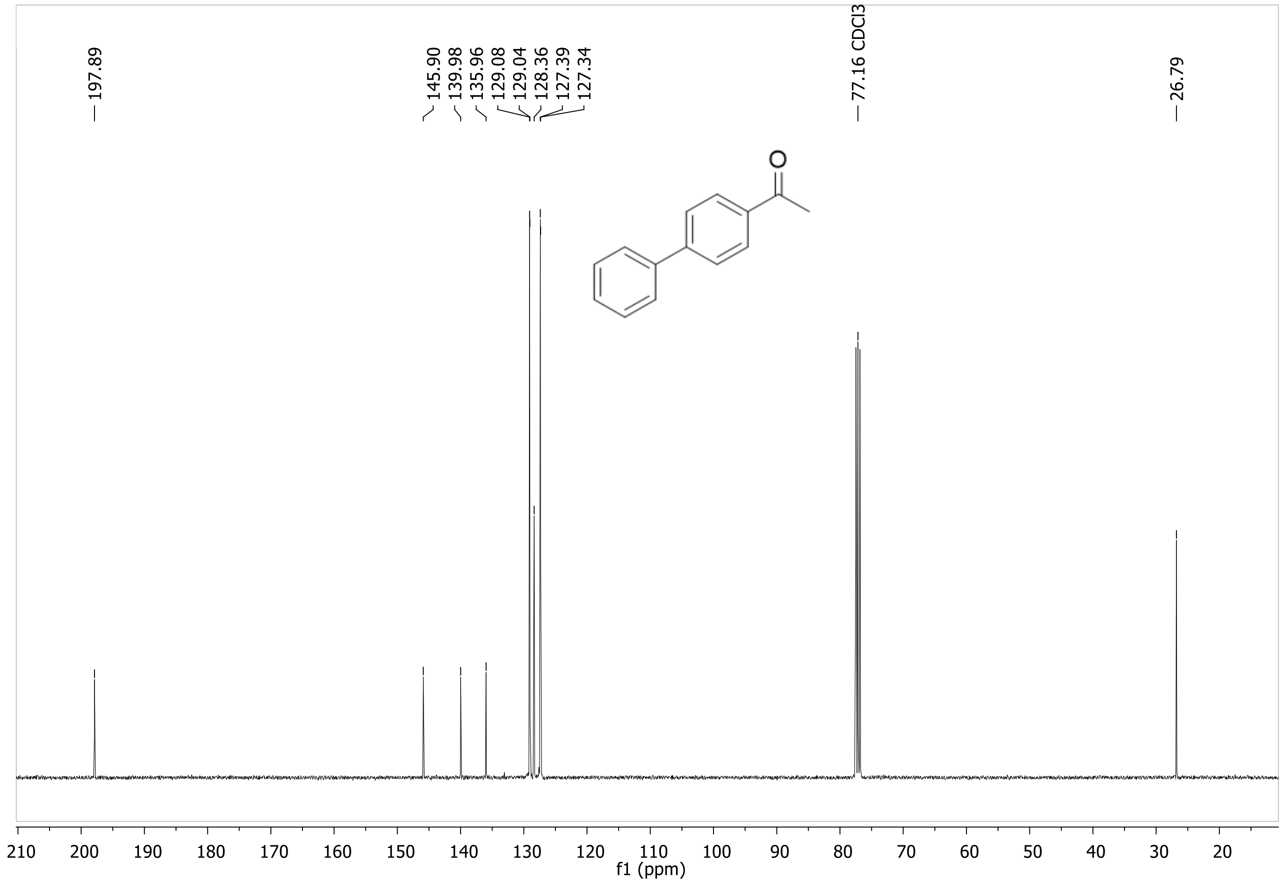

1-(4-Chlorophenyl)ethan-1-one (2i)

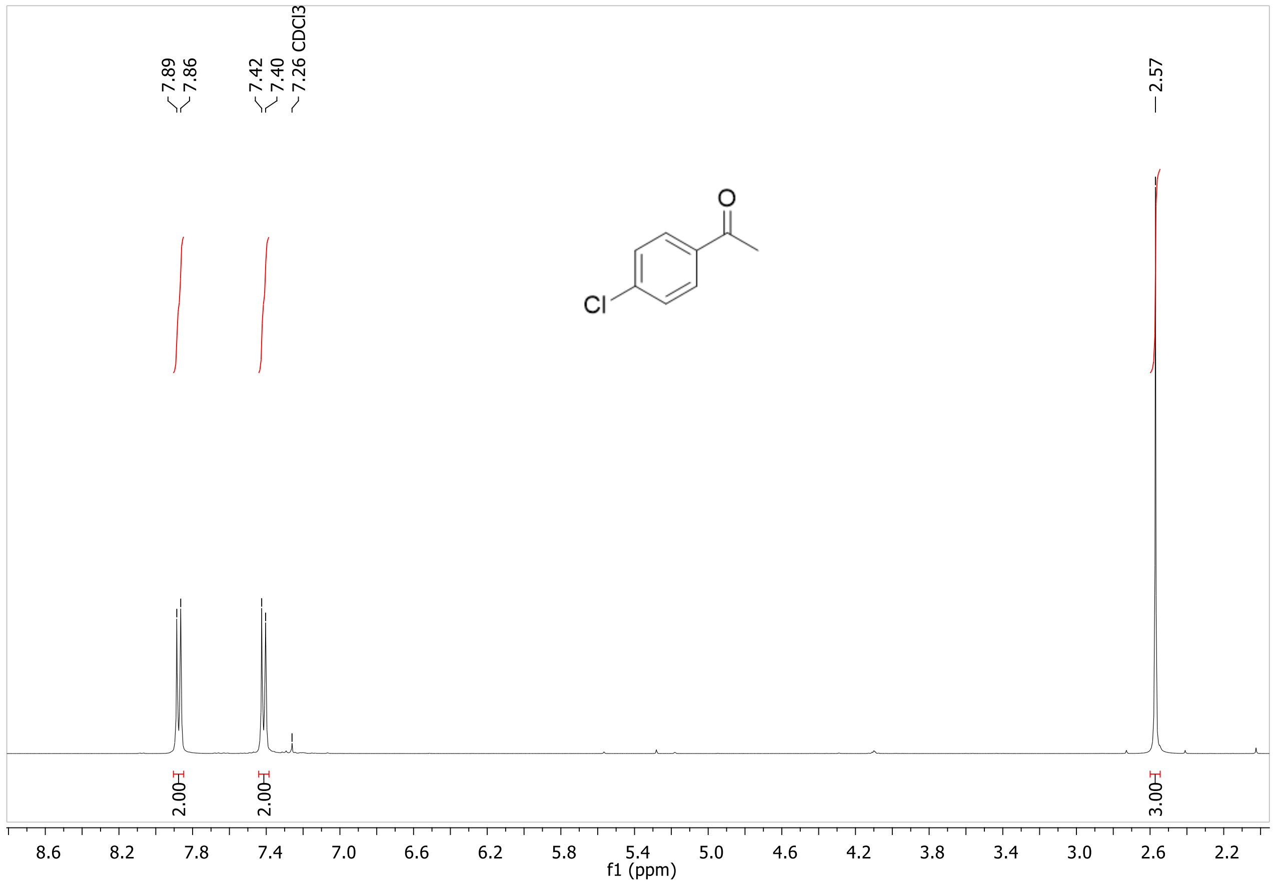

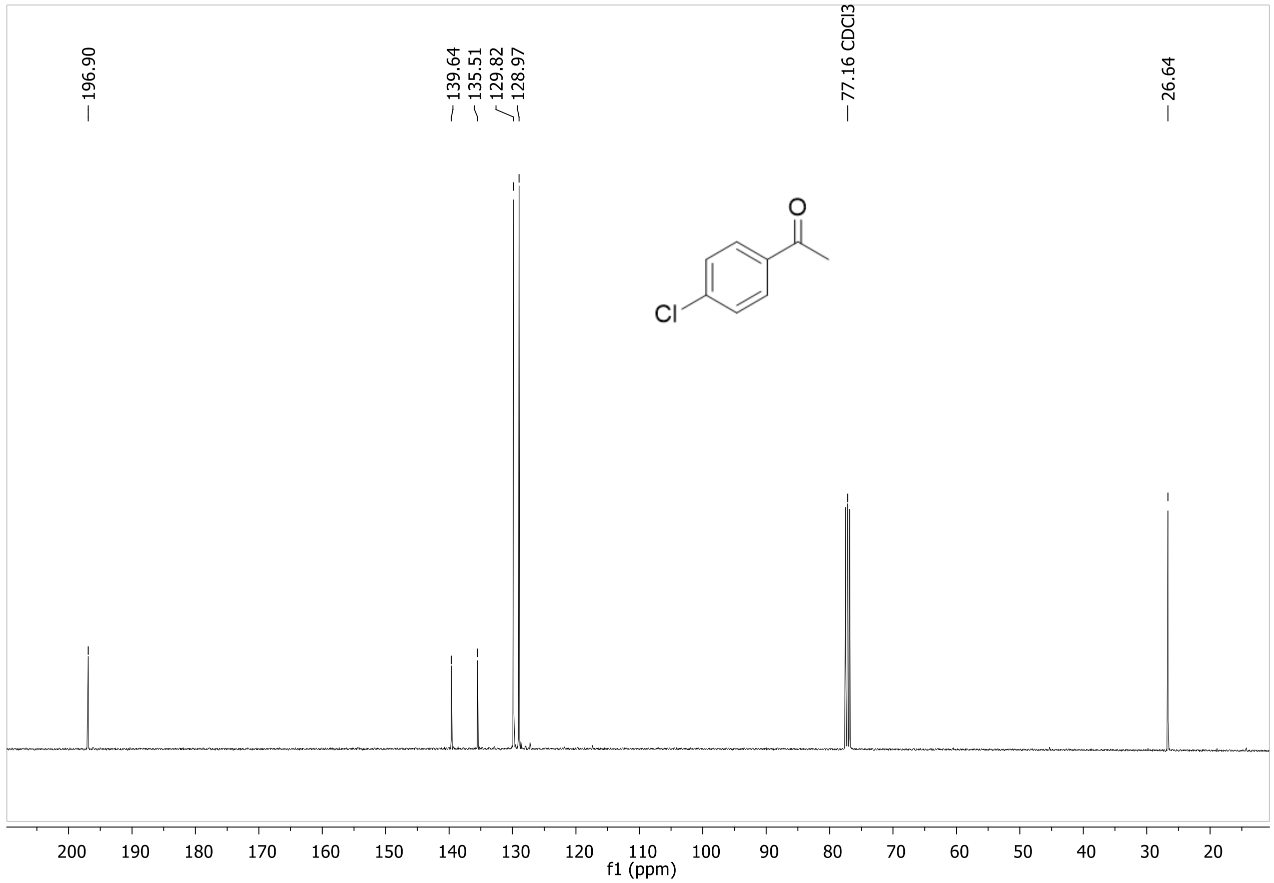

1-(4-Ethynylphenyl)ethan-1-one (2j)

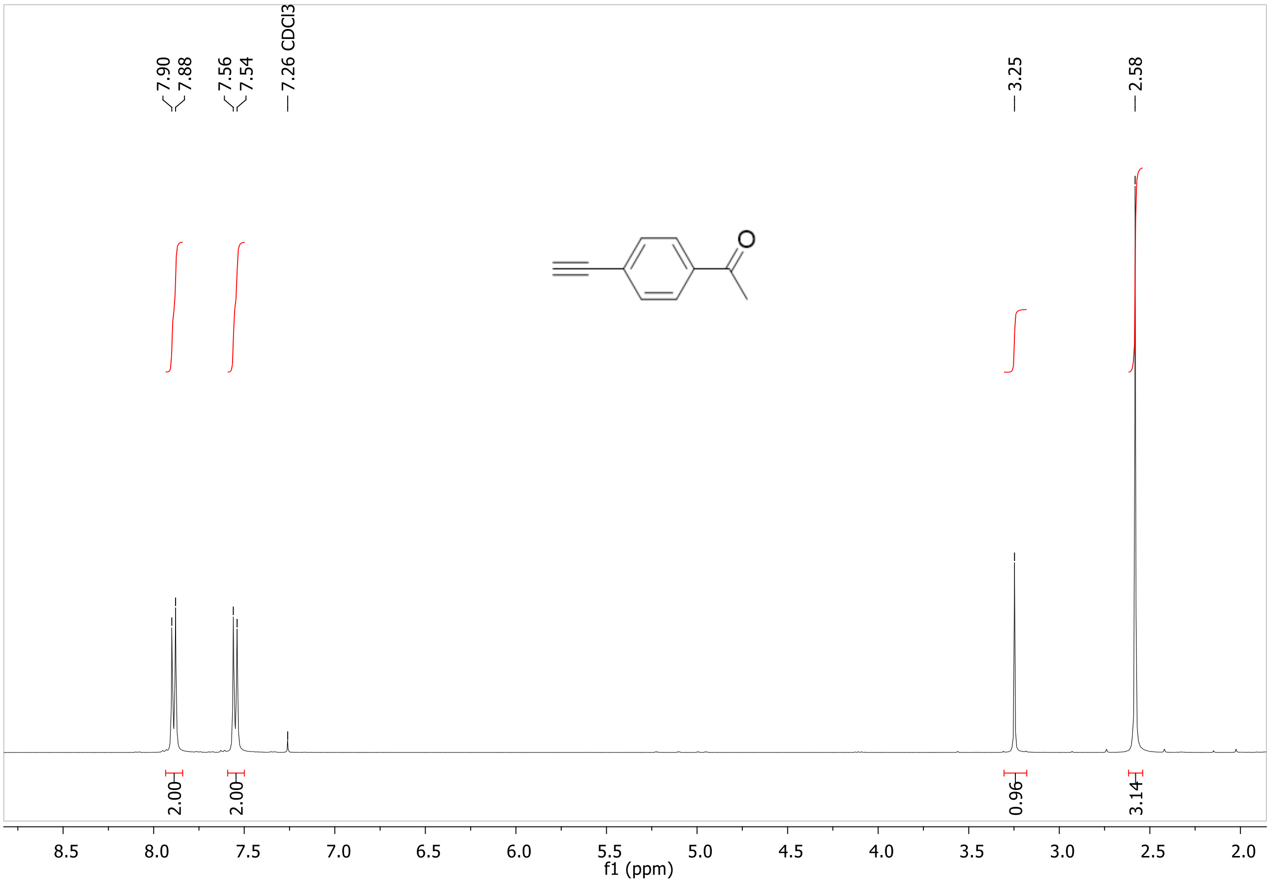

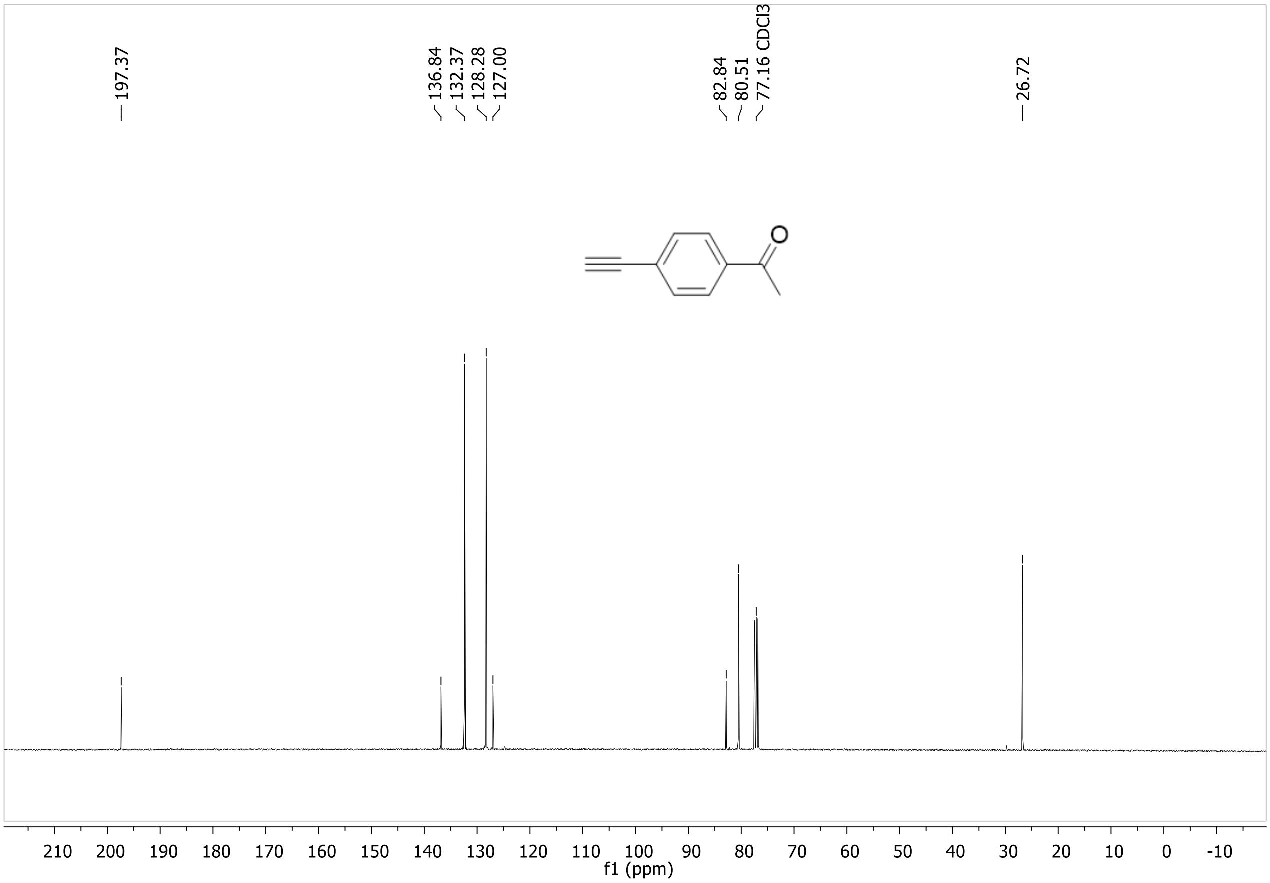

1,1'-(1,4-Phenylene)bis(ethan-1-one) (2jj)

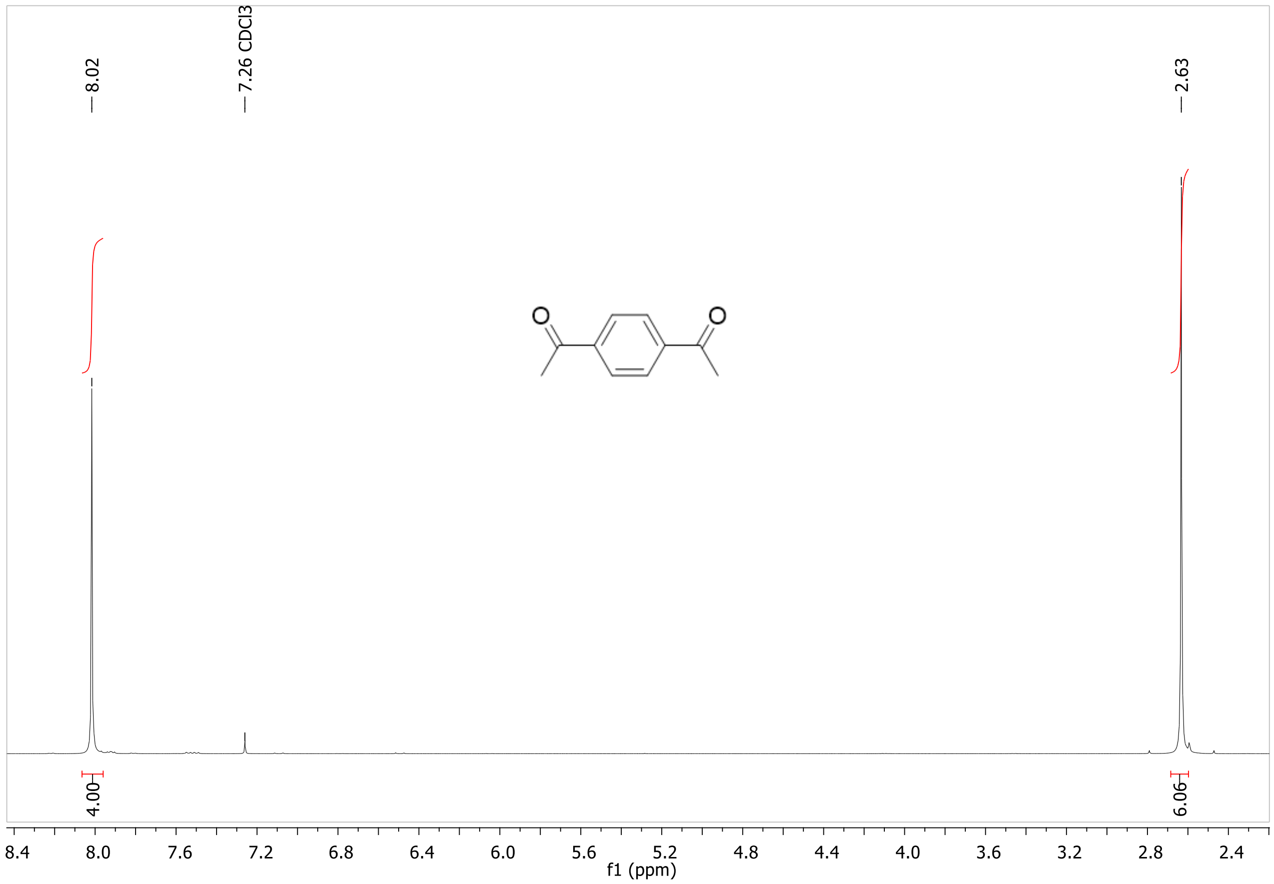

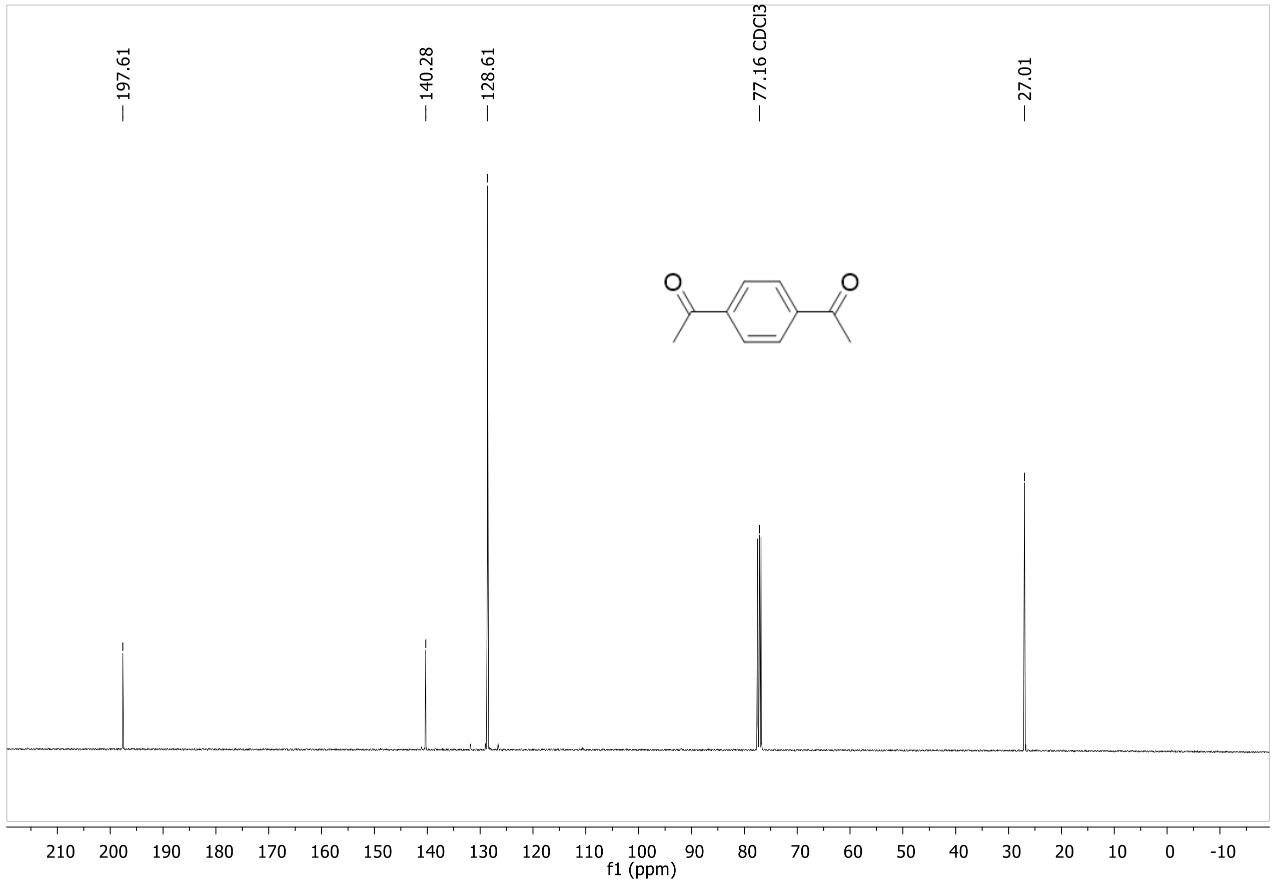

4-Phenylbutan-2-one (2k)

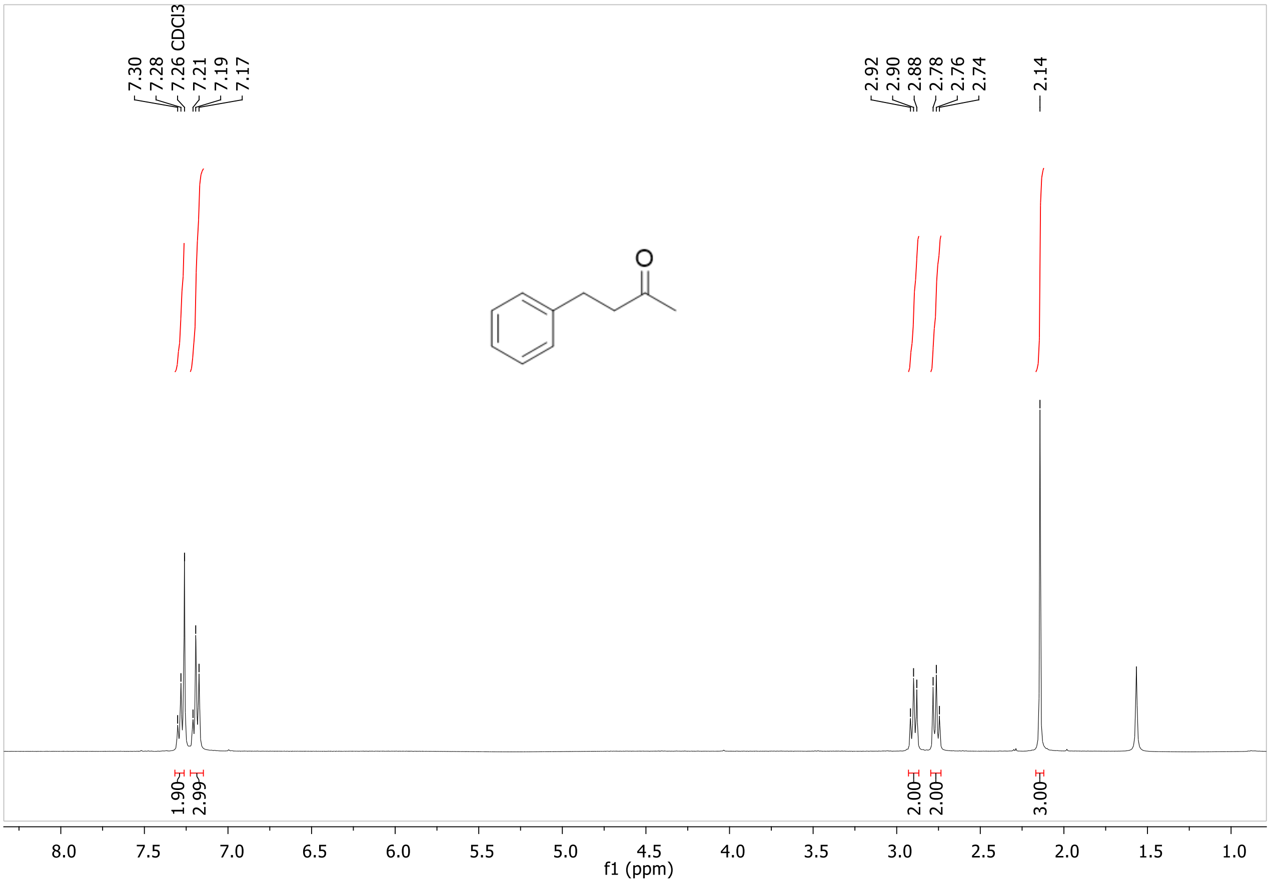

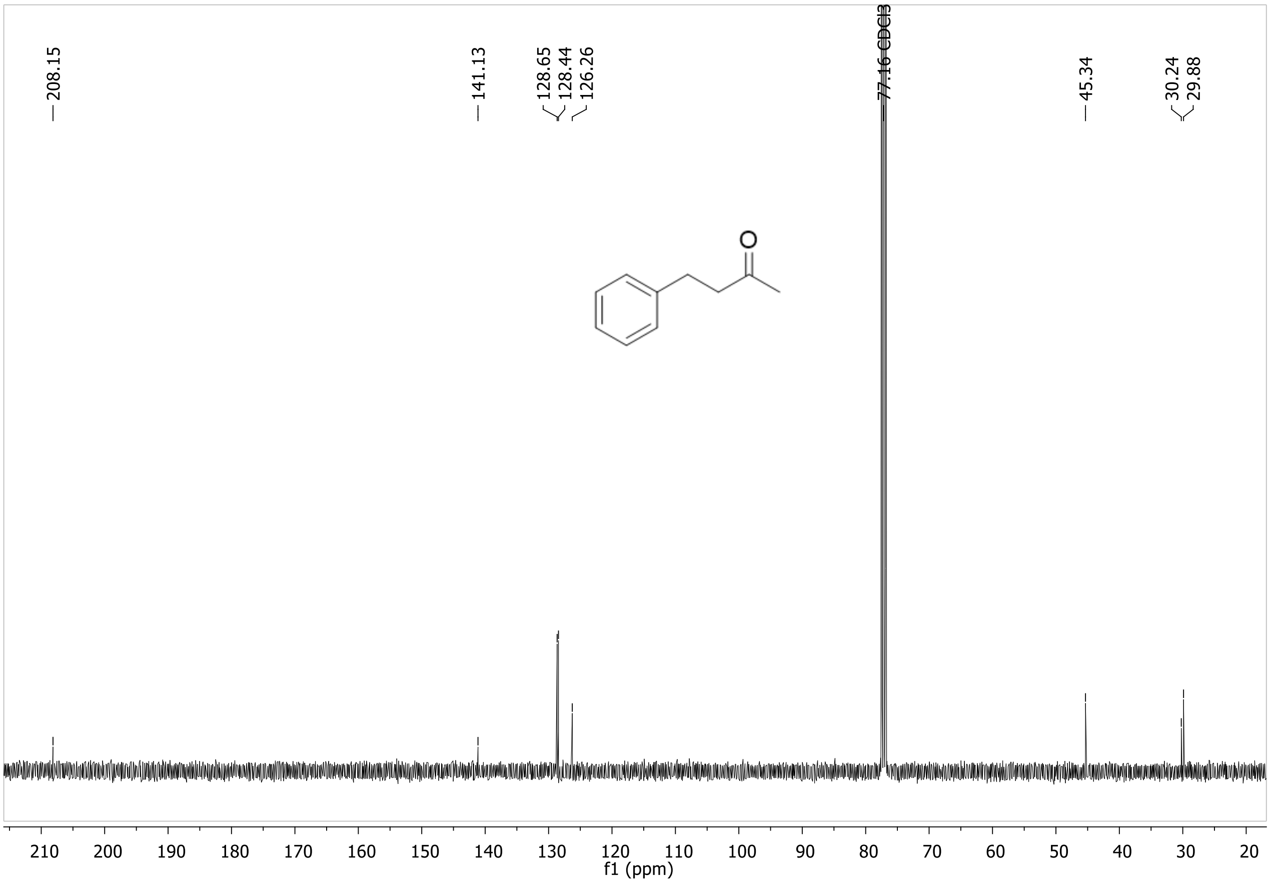

1-Phenylpropan-2-one (2l)

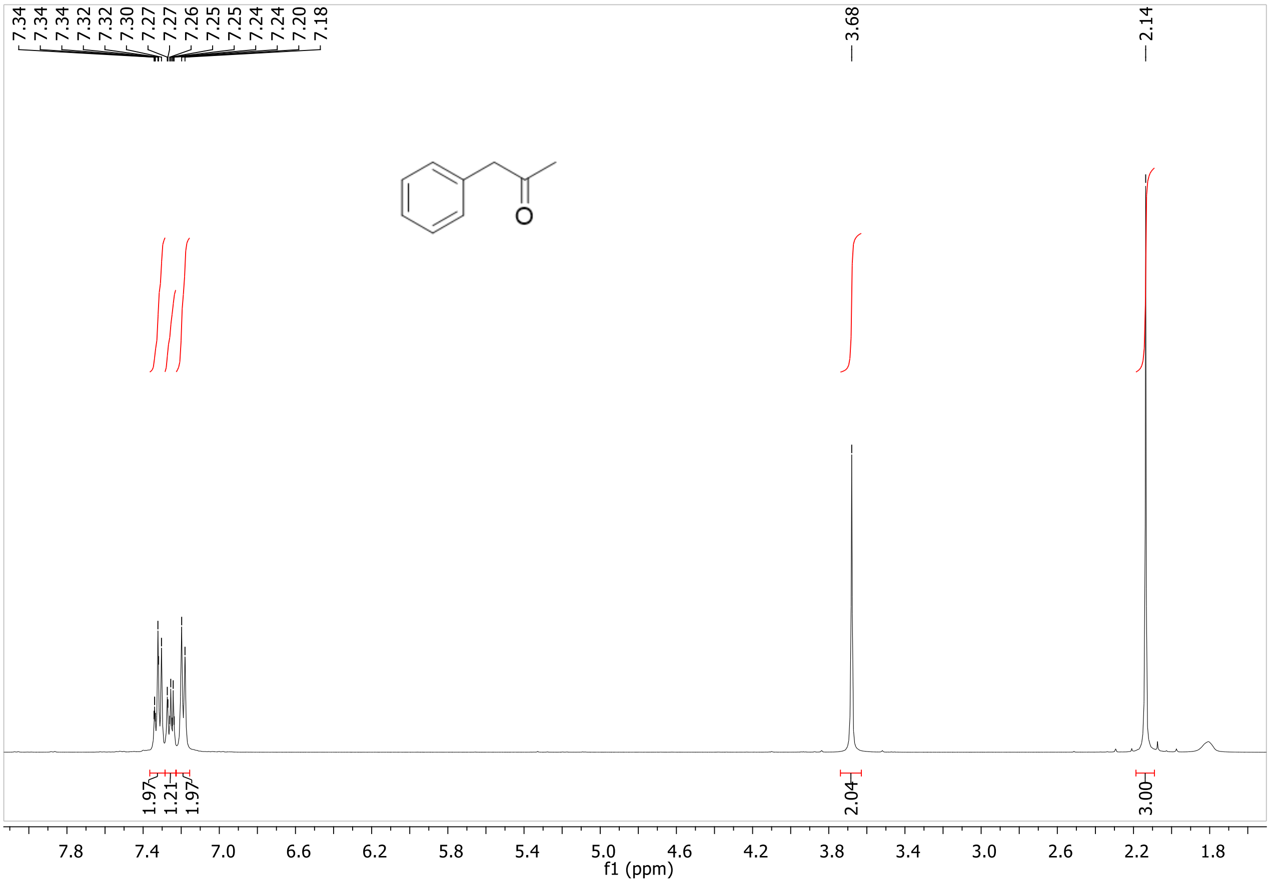

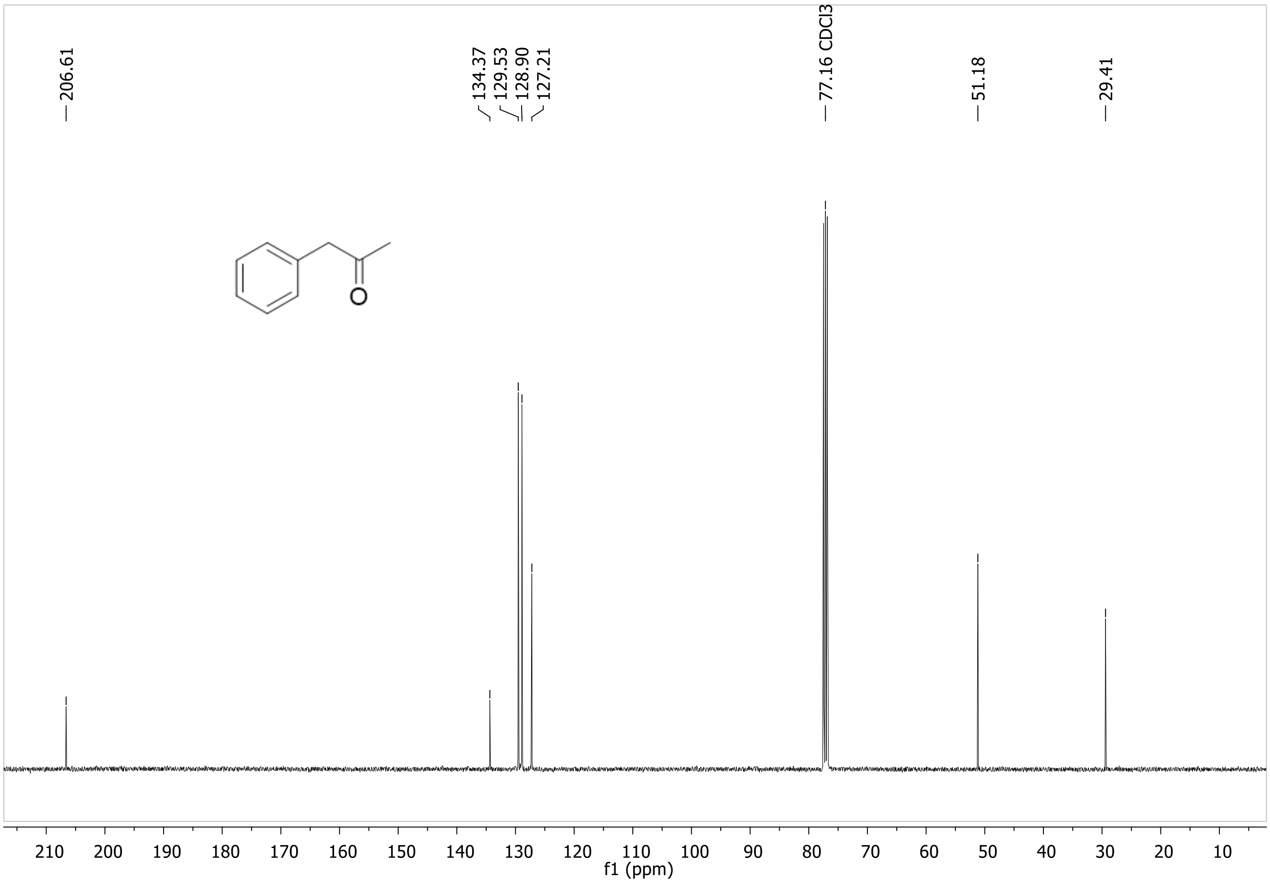

Ethyl 3-oxo-3-phenylpropanoate (2m) (keto/enol)

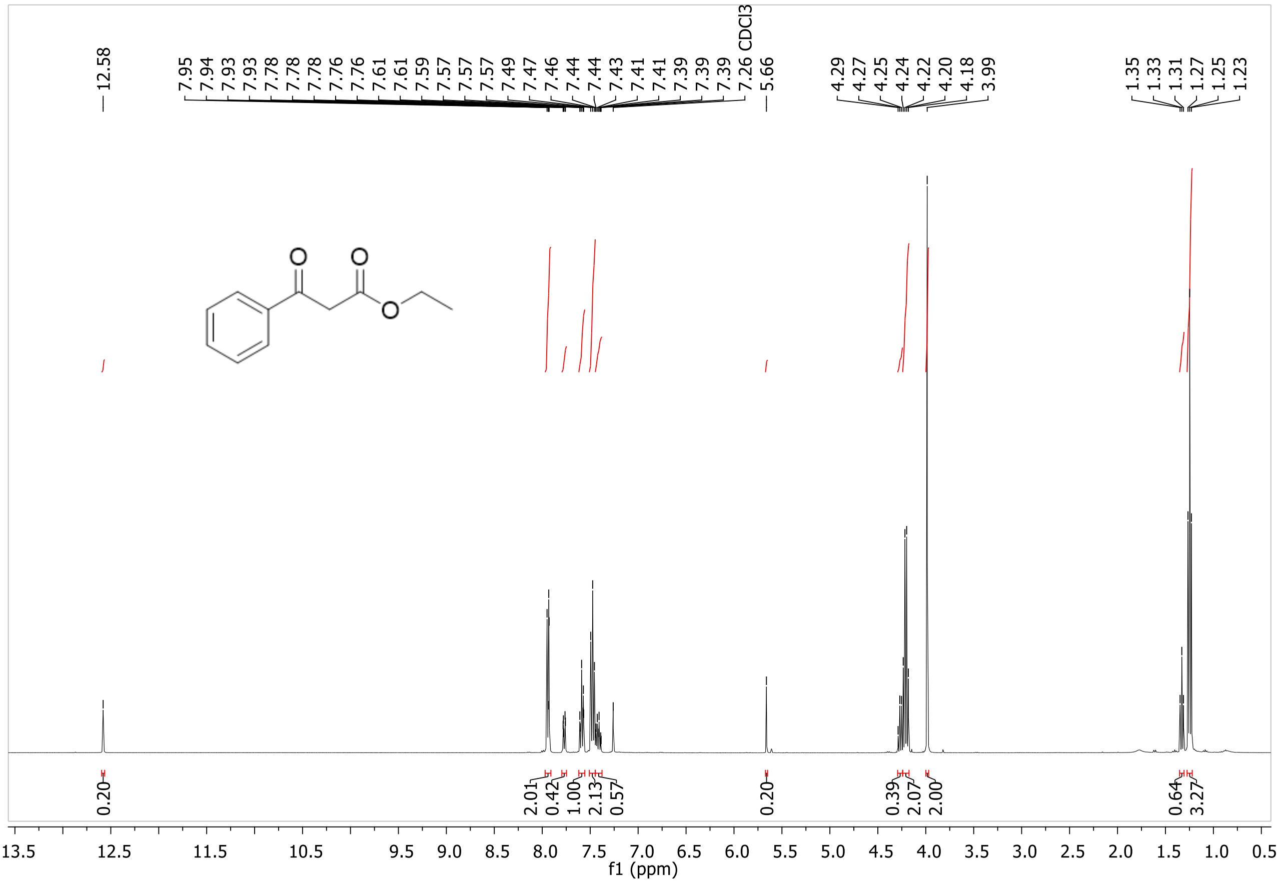

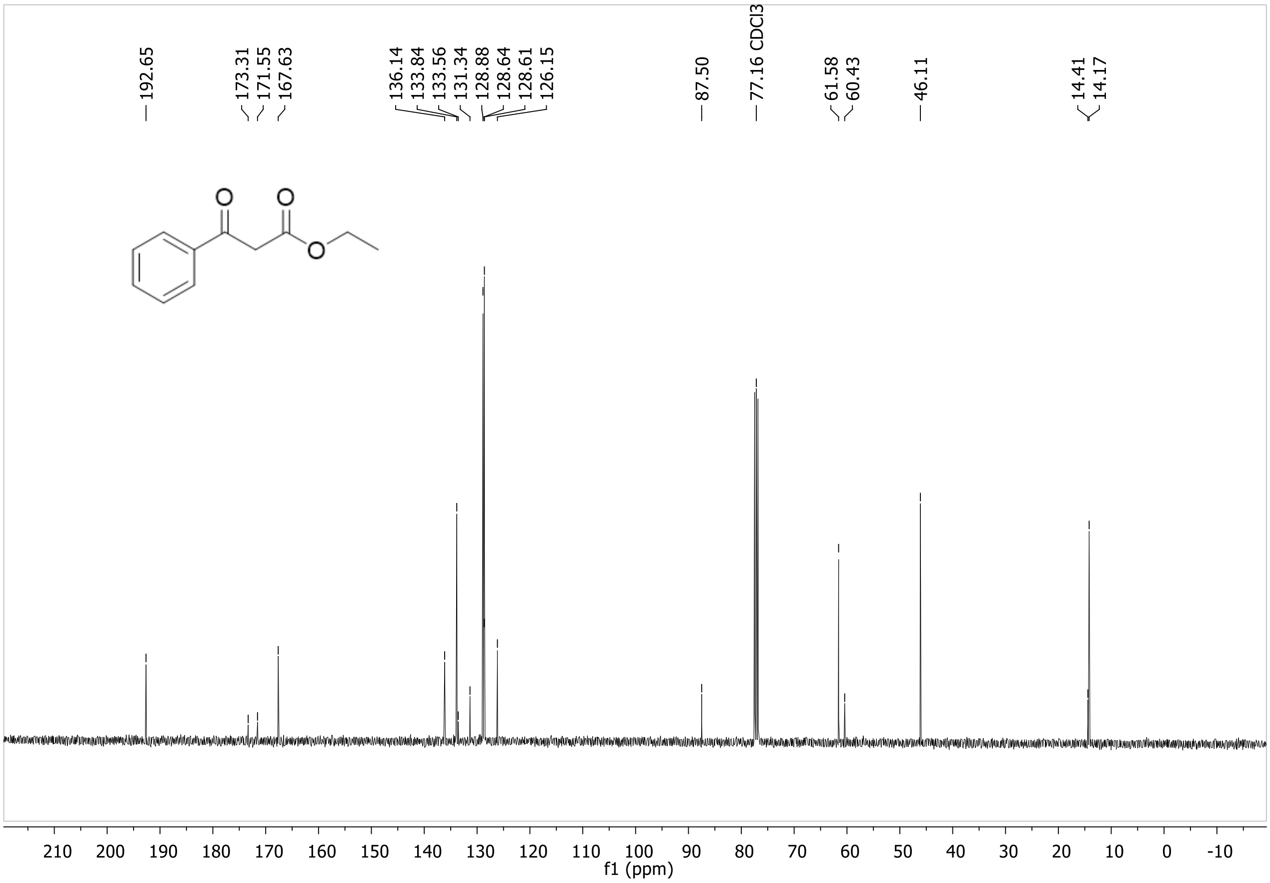

Ethyl 3-(4-chlorophenyl)-3-oxopropanoate (2n) (keto/enol)

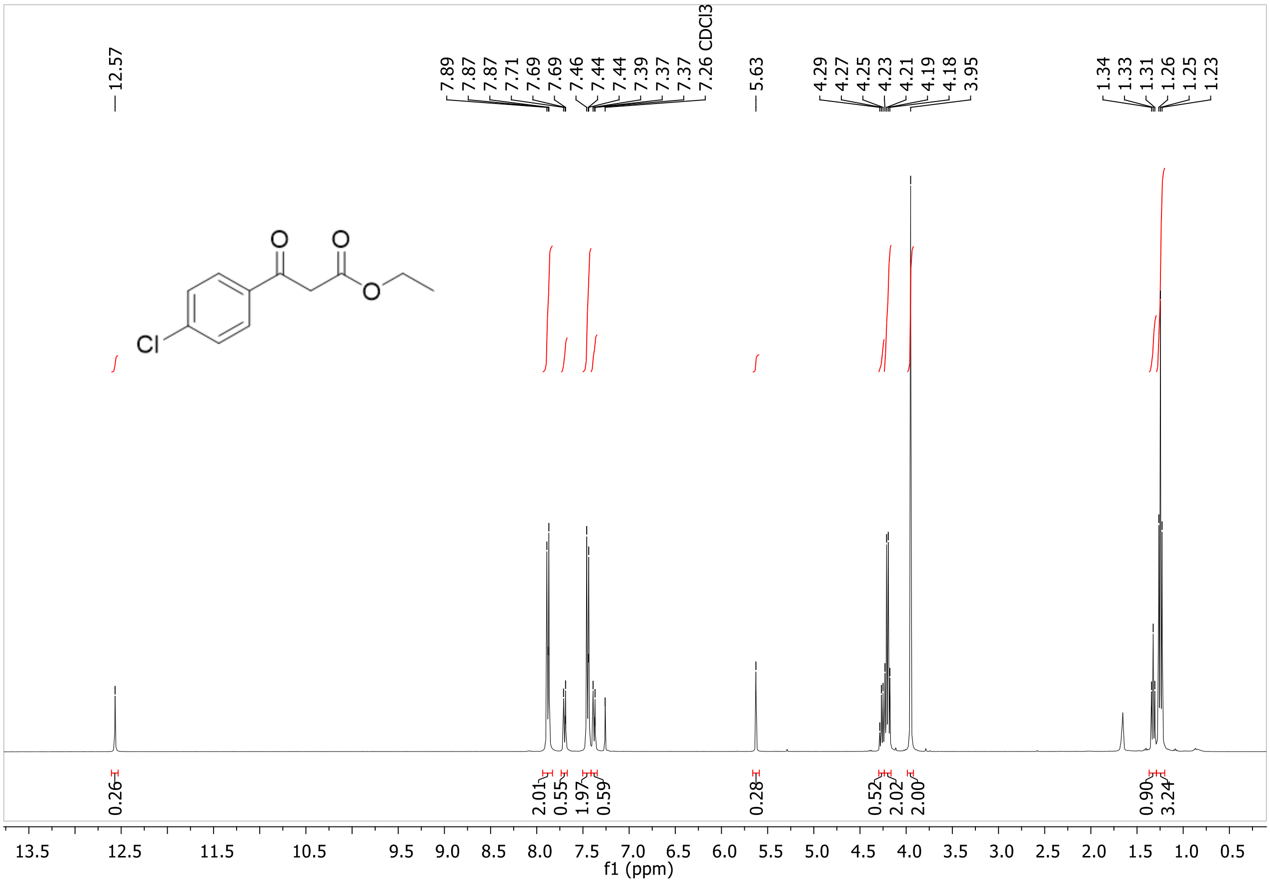

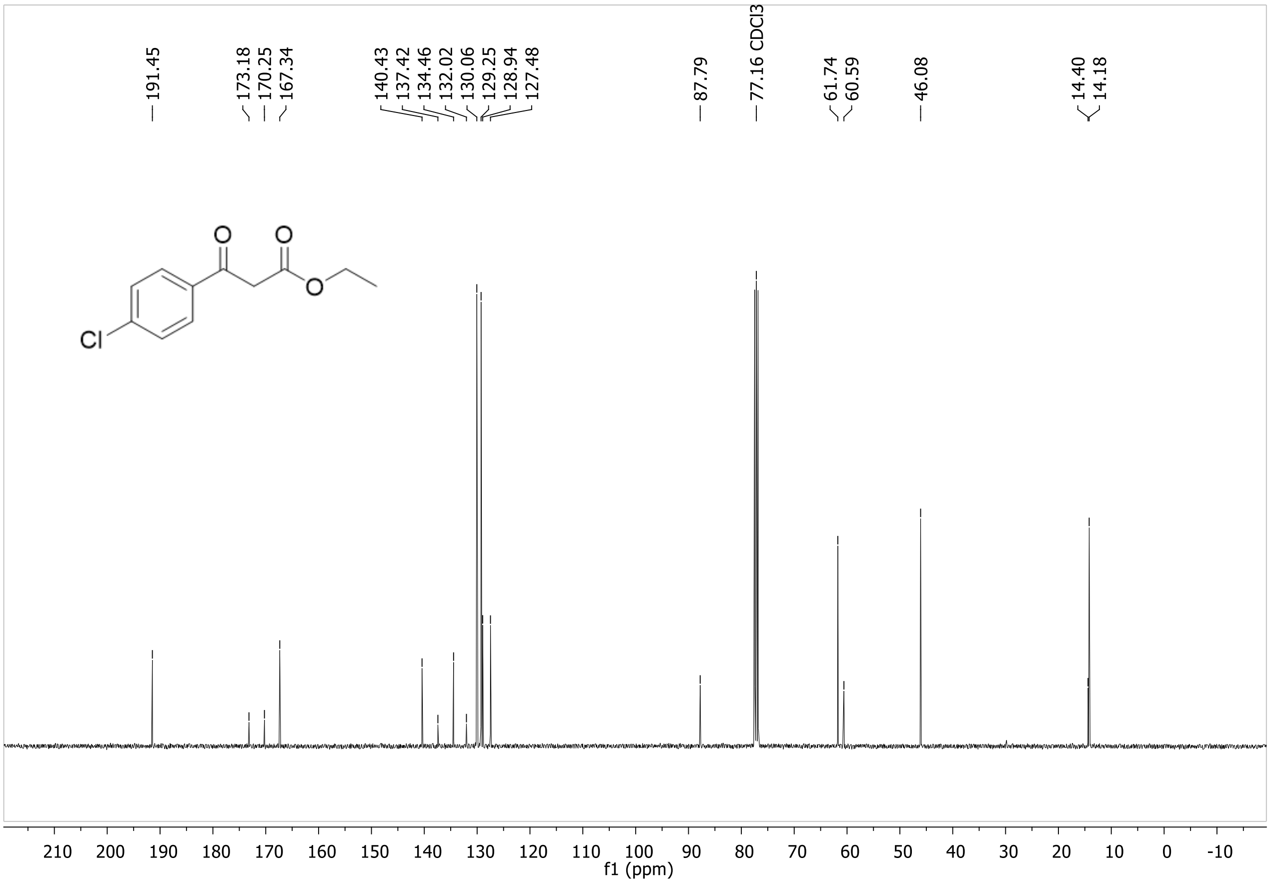

(*E*)-1,3-Diphenylbut-2-en-1-one (3d)

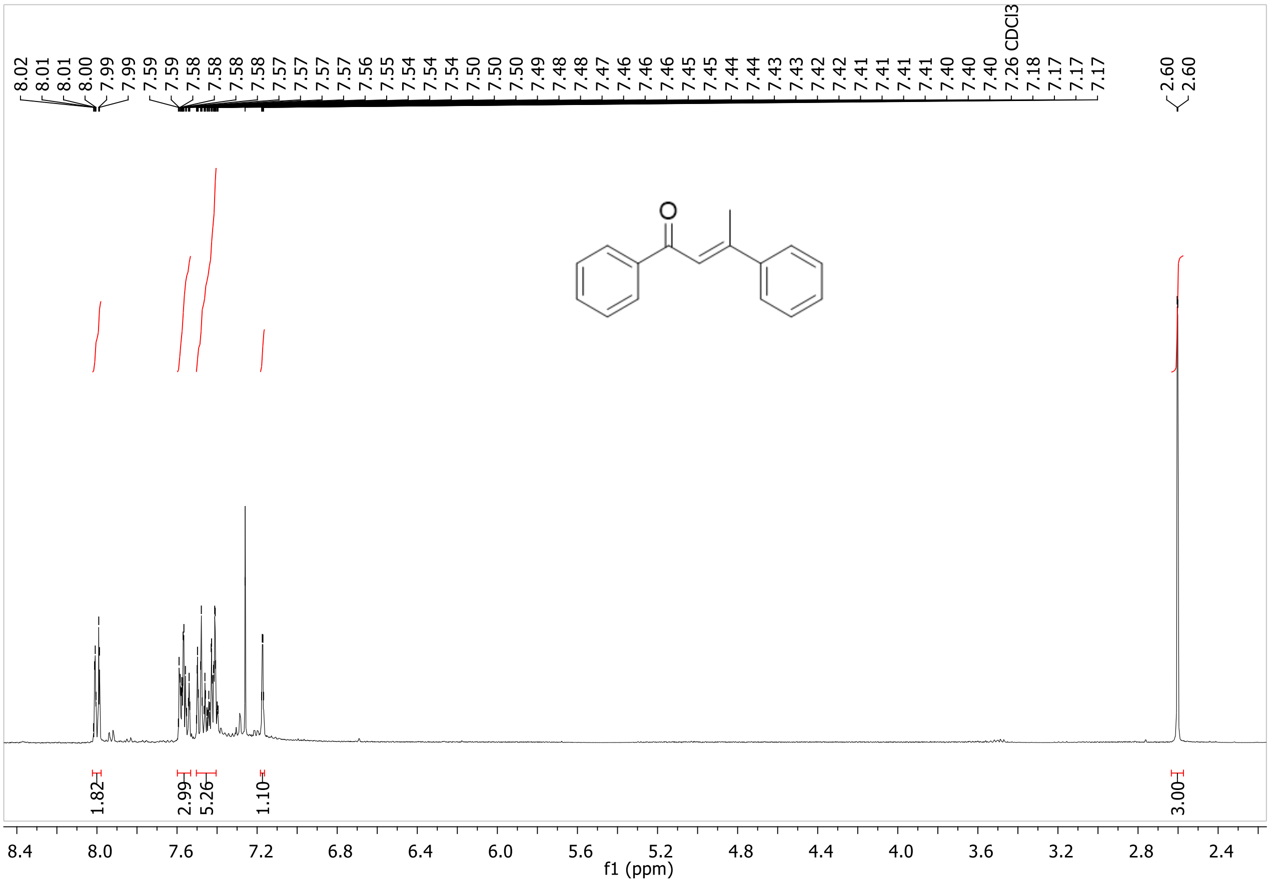

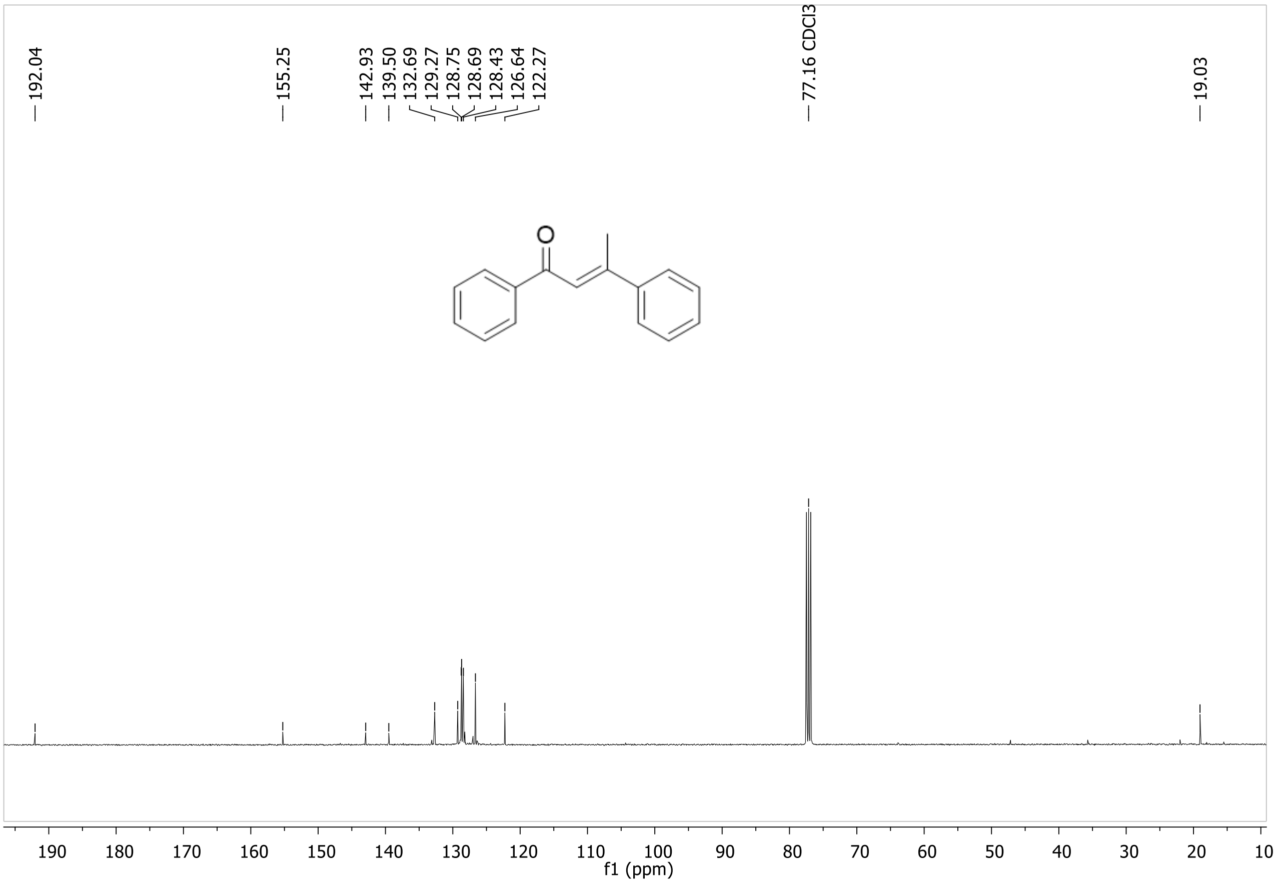

(*E*)-1,3-Di-*p*-tolylbut-2-en-1-one (3e)

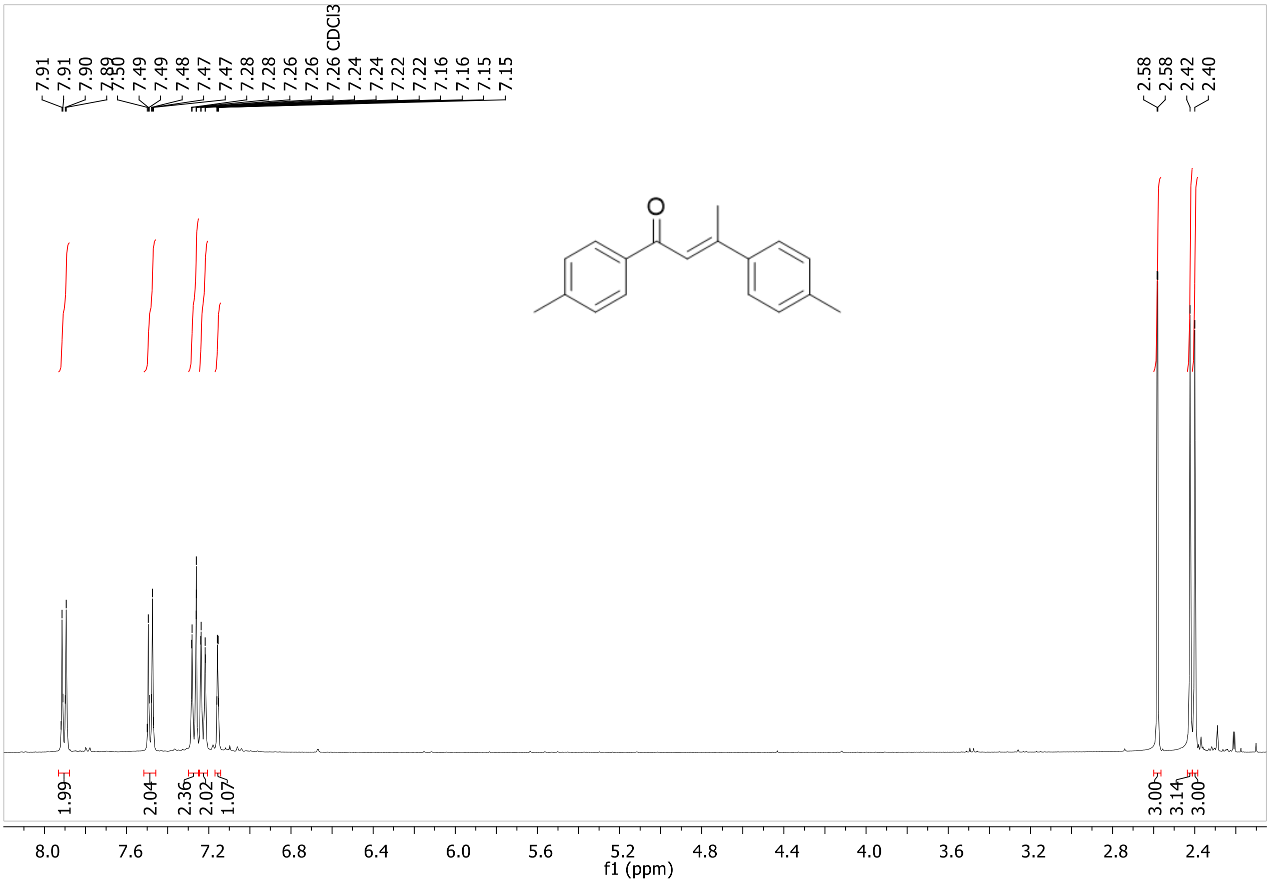

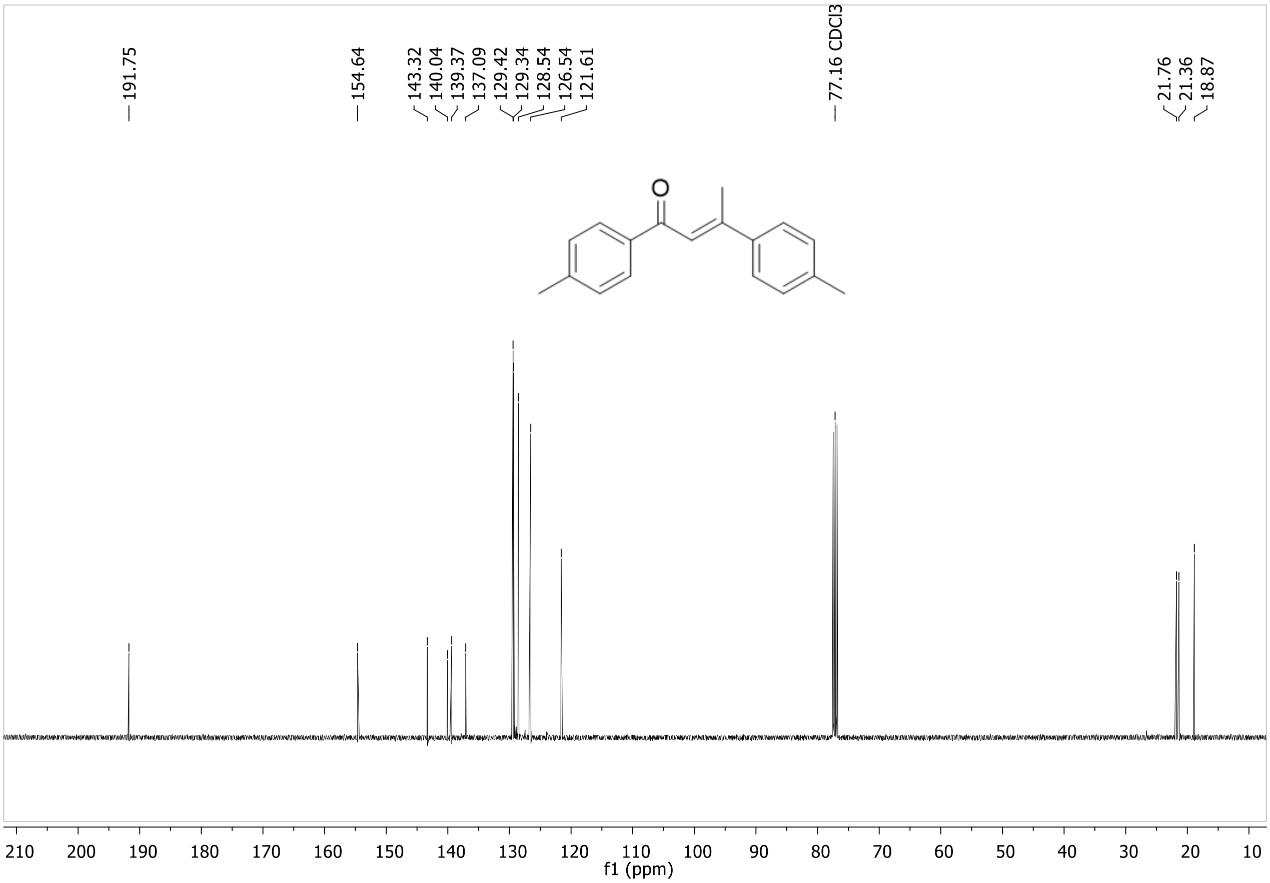

(*E*)-1,3-Di-*m*-tolylbut-2-en-1-one (3f)

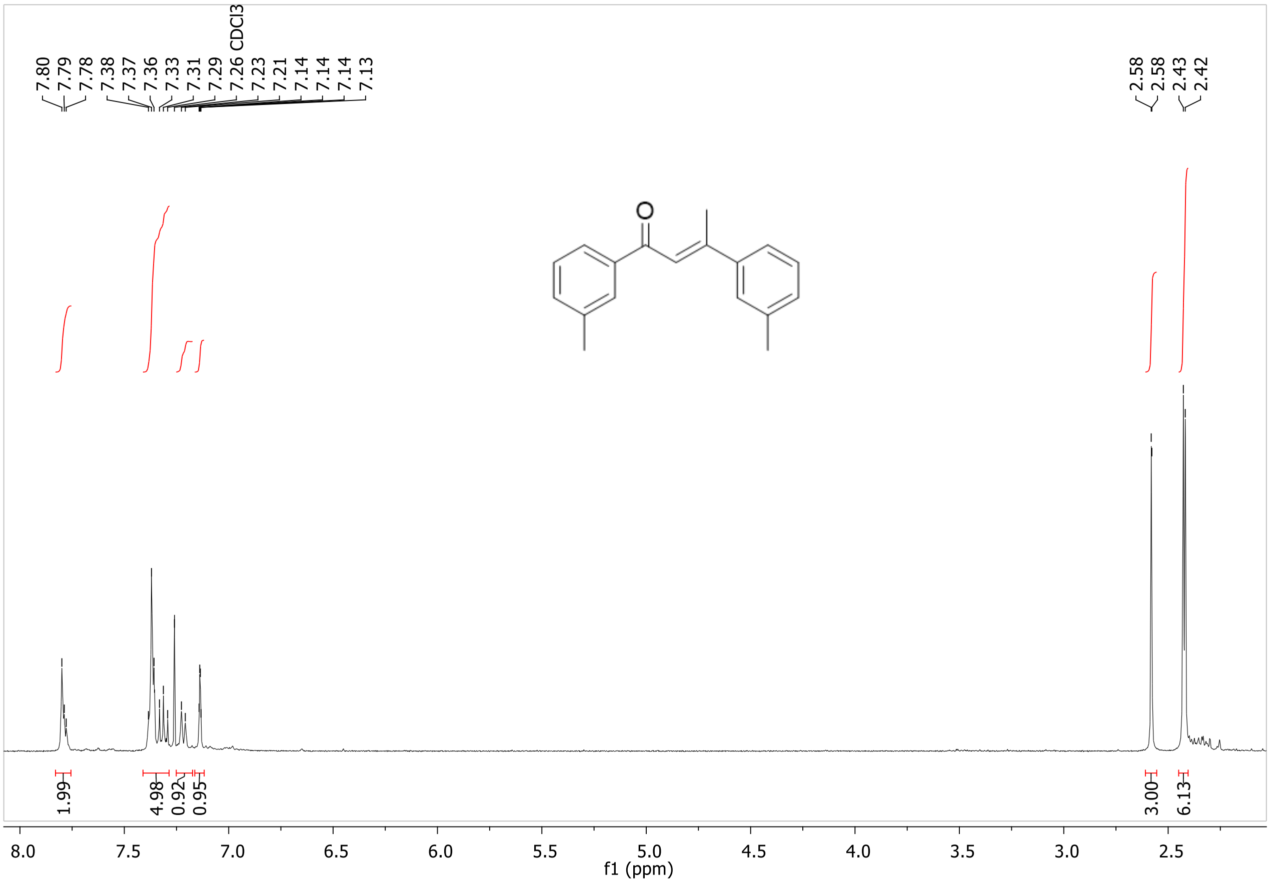

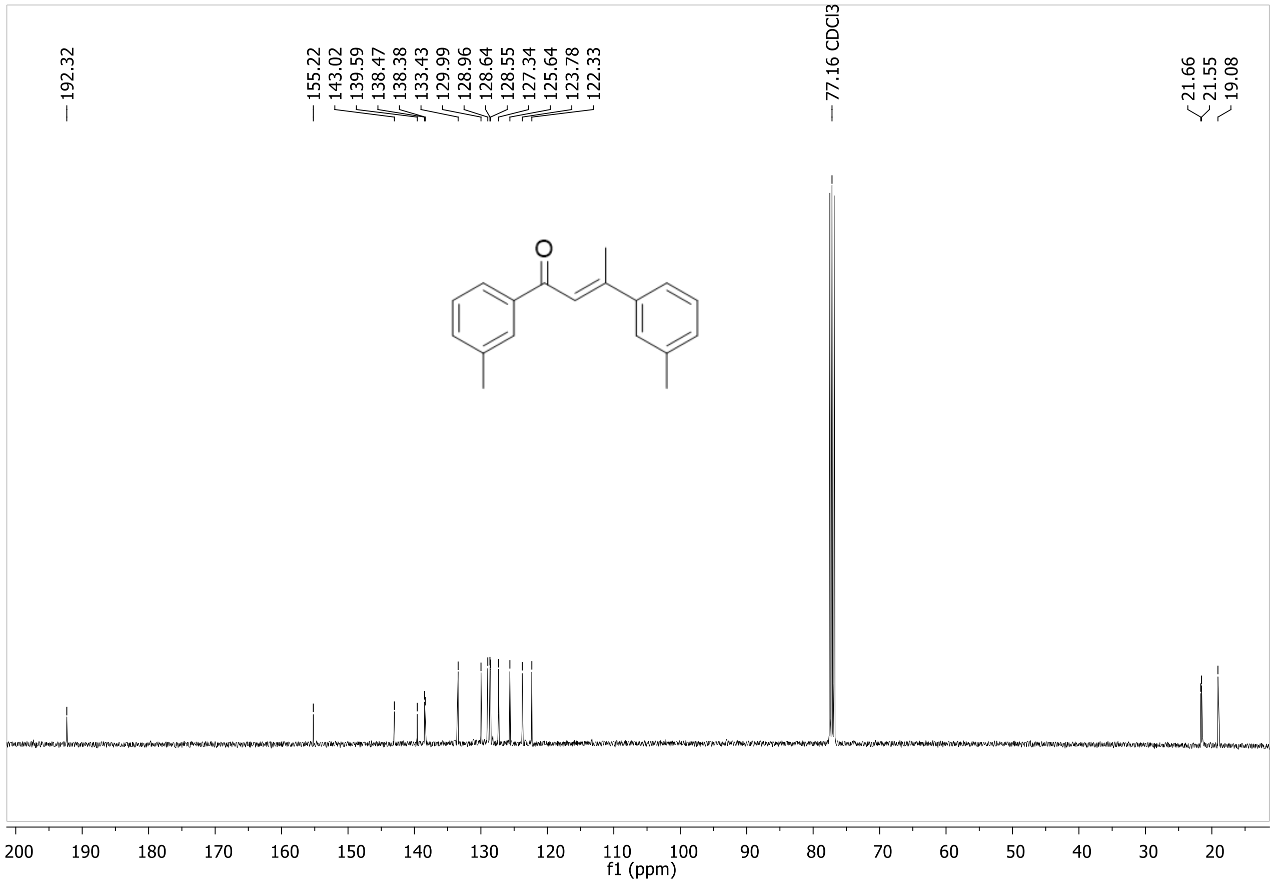

1,3-Di-*o*-tolylbut-2-en-1-one (E/Z mixture) (3g)

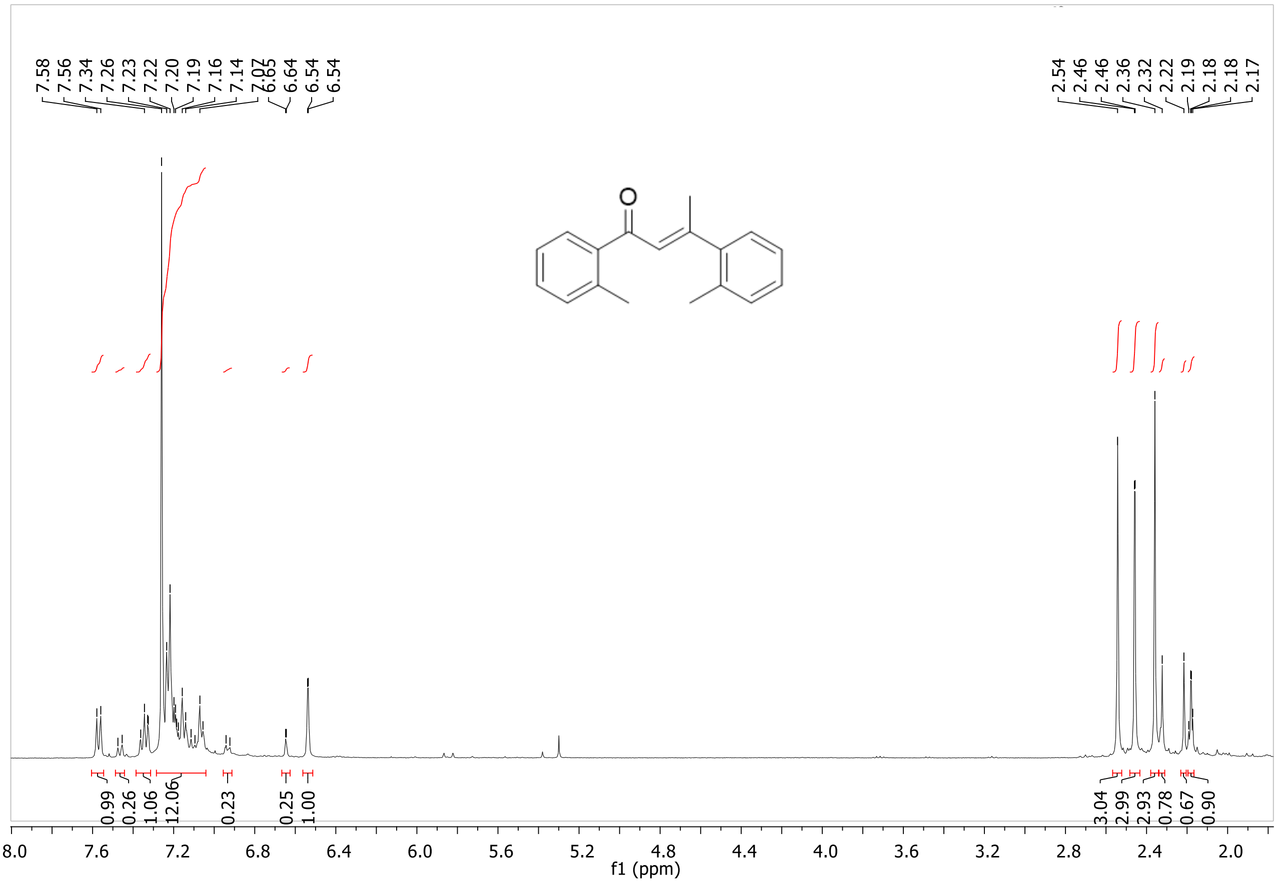

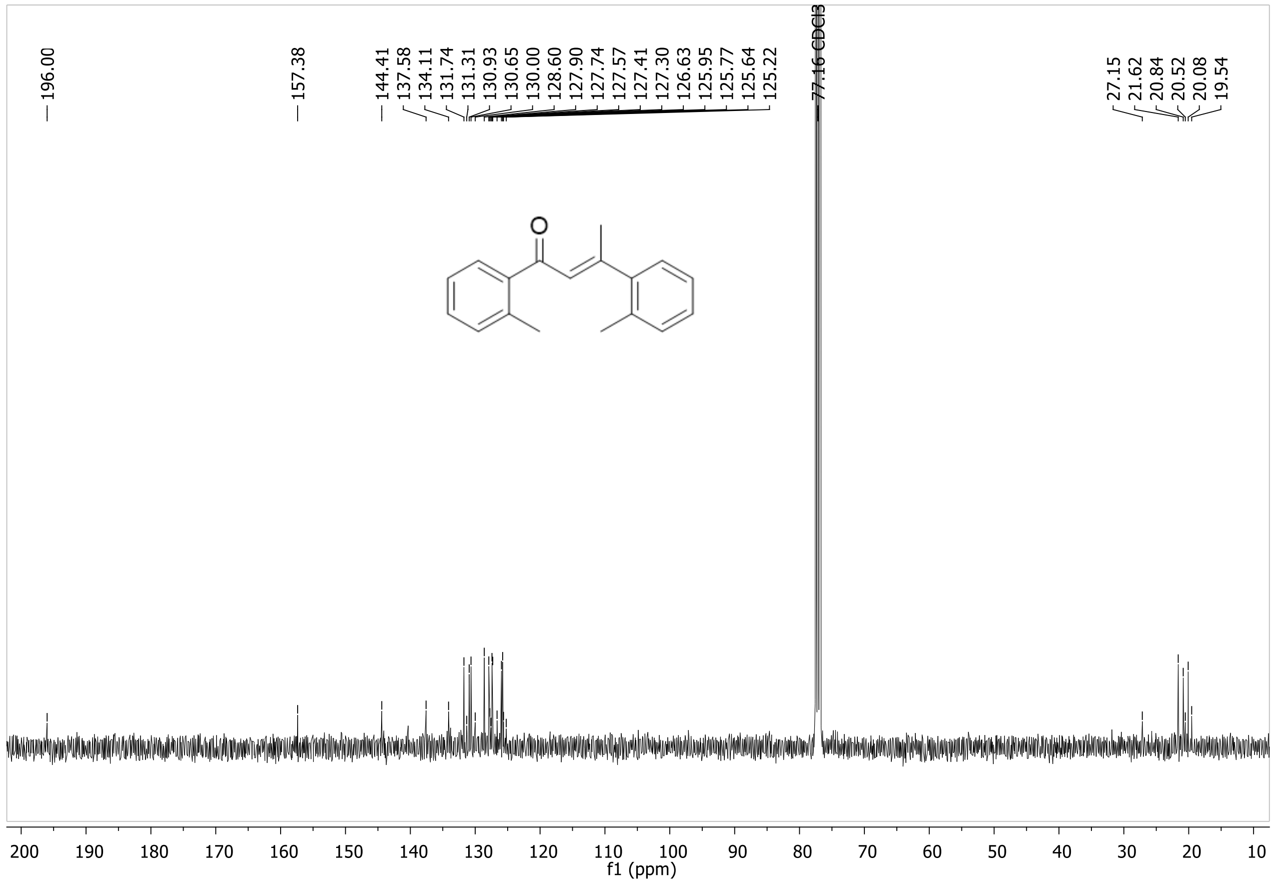

(*E*)-1,3-Di([1,1'-biphenyl]-4-yl)but-2-en-1-one (3h)

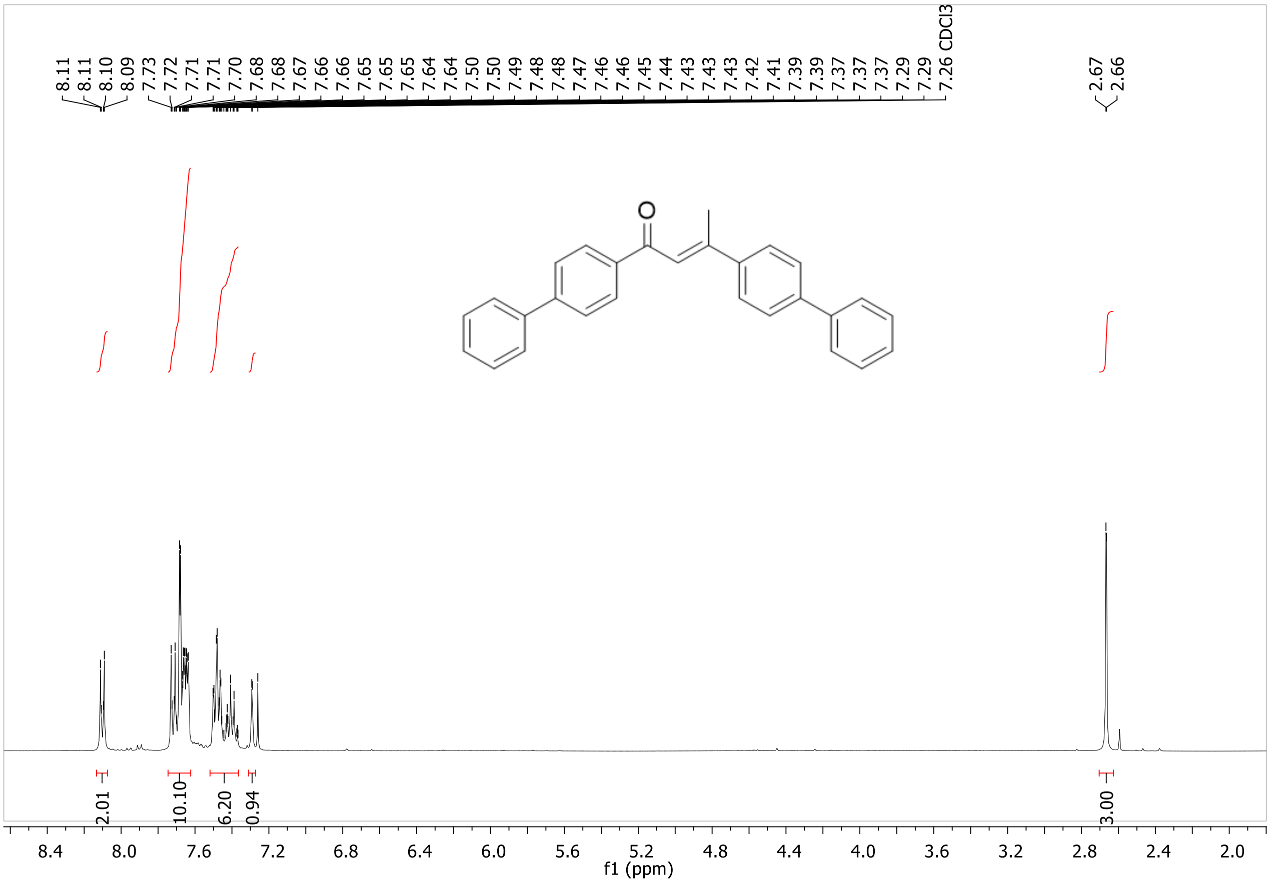

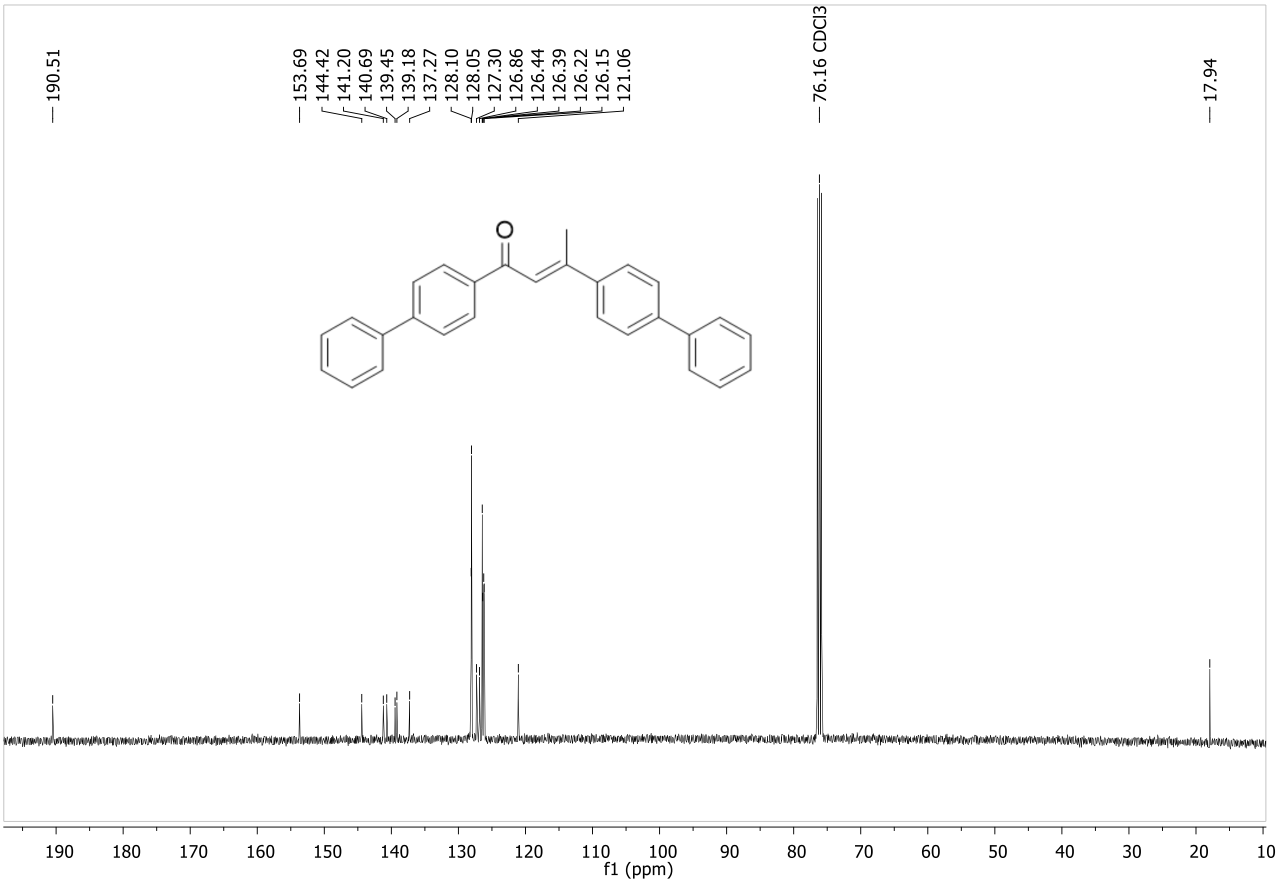

(*E*)-1,3-Bis(4-chlorophenyl)but-2-en-1-one (3i)

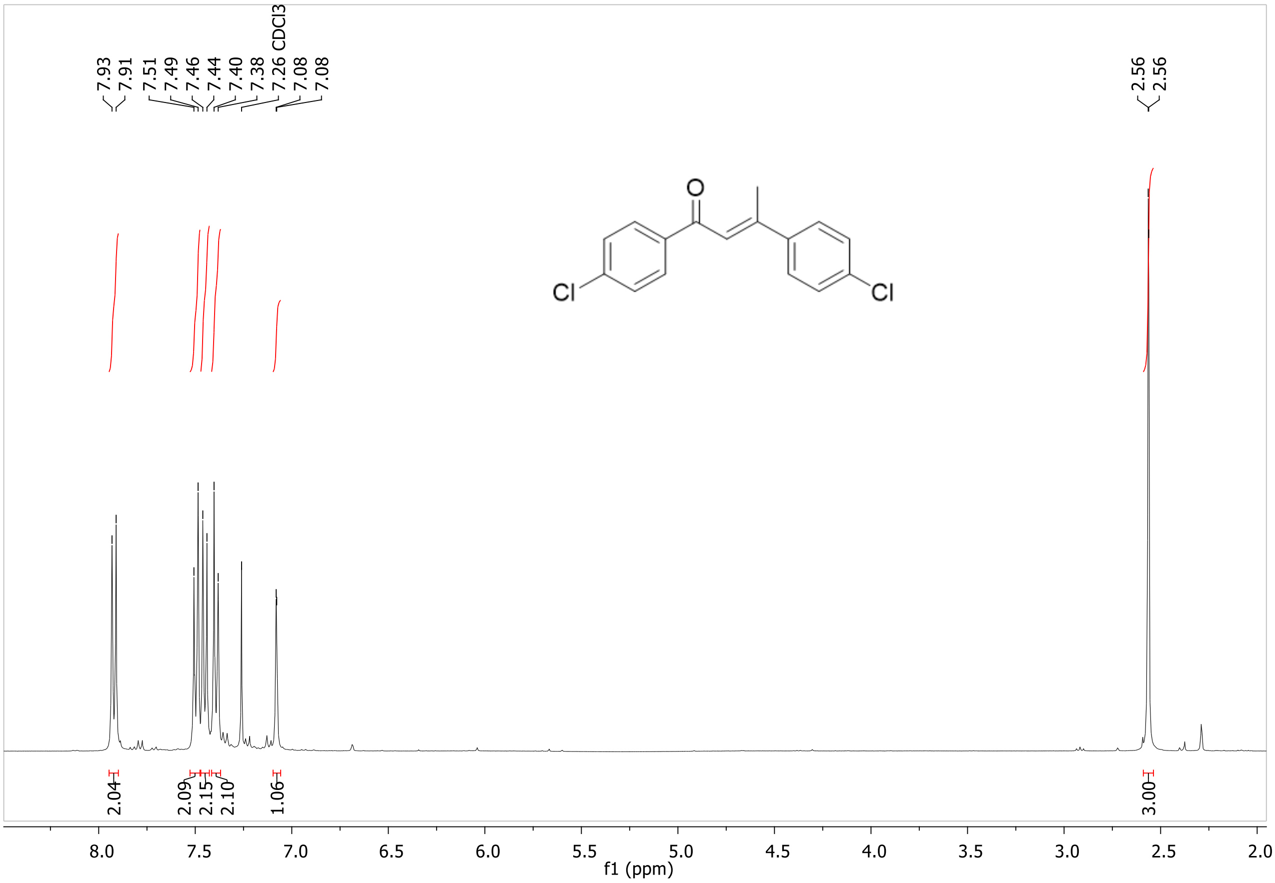

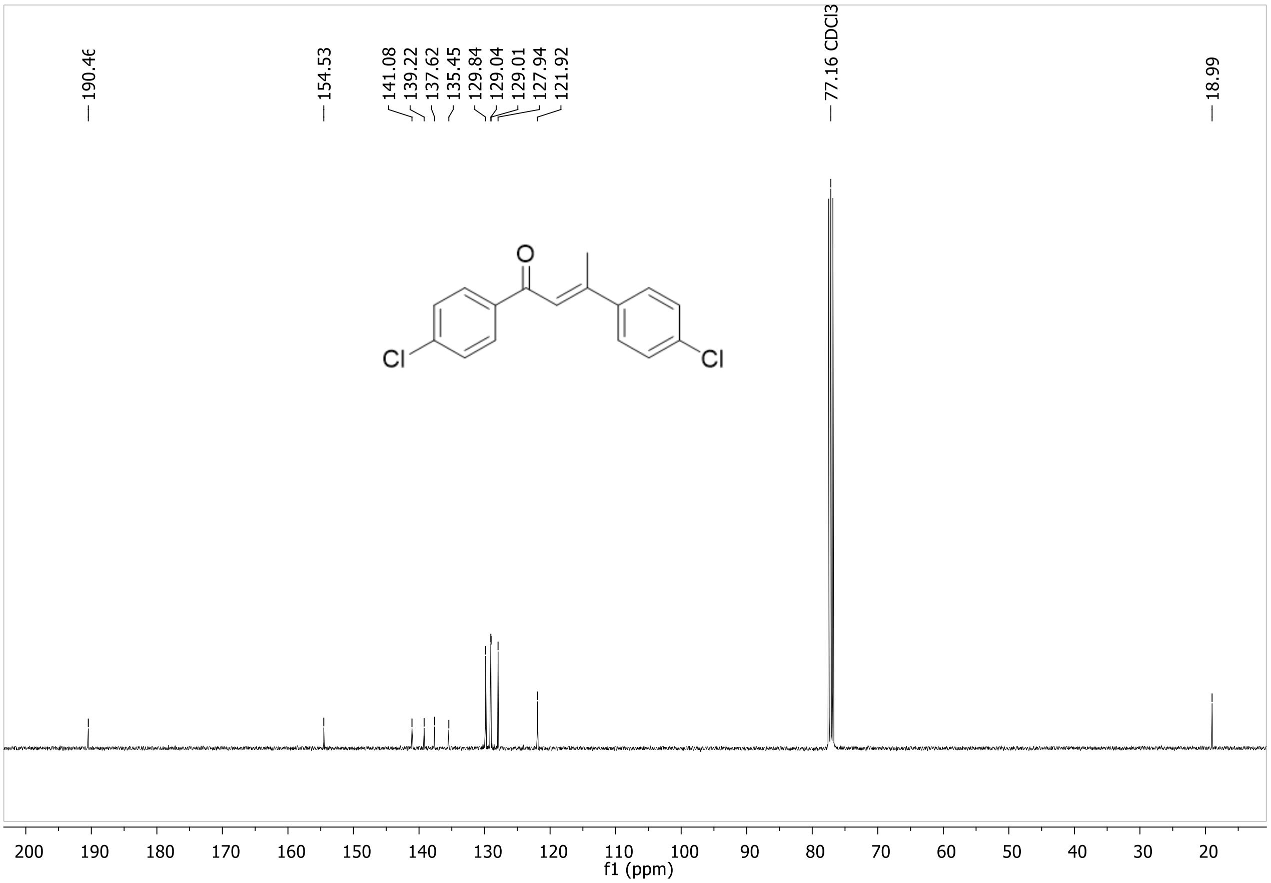

References

1 M. Zheng, C. Huang, J.-Z. Yan, S.-L. Xie, S.-J. Ke, H.-D. Xia and Y.-N. Duan, In Situ Hypoiodite-Catalyzed Oxidative Rearrangement of Chalcones: Scope and Mechanistic Investigation, *J Org Chem*, 2023, **88**, 1504–1514.

2 A. L. Sandoval, K. E. Doherty, G. P. Wadey, C. M. Schroeder and N. E. Leadbeater, Fast, easy oxidation of alcohols using an oxoammonium salt bearing the nitrate anion, *Tetrahedron Lett*, 2023, **116**, 154332.

3 H. Wu, S. Chen, D. Xiao, F. Li, K. Zhou, X. Yin, C. Liu, X. He and Y. Shang, Visible-Light-Mediated Deacylated Alkynylation of Unstrained Ketone, *Org Lett*, 2023, **25**, 1166–1171.

4 S. Zhang, J. Zhang and H. Zou, Pd-Catalyzed TBHP-Mediated Selective Wacker-Type Oxidation and Oxo-acyloxylation of Olefins Using a 2-(1 *H* -Indazol-1-yl)quinoline Ligand, *Org Lett*, 2023, **25**, 1850–1855.

5 J. Huang, X. Yan and Y. Xia, Directing Group-Free Formal Suzuki–Miyaura Coupling of Simple Ketones Enabled by Activation of Unstrained C−C Bonds, *Angew Chem Int Ed Engl.*, 2022, **61**, e202211080.

6 A. Ikeda, M. Omote, K. Kusumoto, A. Tarui, K. Sato and A. Ando, One-pot synthesis of 1,3-enynes with a CF_3 group on the terminal sp^2 carbon by an oxidative Sonogashira cross-coupling reaction, *Org Biomol Chem*, 2015, **13**, 8886–8892.

7 J. Liu, K.-F. Hu, J.-P. Qu and Y.-B. Kang, Organopromoted Selectivity-Switchable Synthesis of Polyketones, *Org Lett*, 2017, **19**, 5593–5596.

8 A. K. Jha, R. Kumari and S. Easwar, Synthesis of 2,2-Disubstituted Dihydro-1,4-benzothiazines from Morita–Baylis–Hillman Ketones by an Oxidative Cyclization, *J Org Chem*, 2022, **87**, 5760–5772.

9 L. Saney, K. E. Christensen, X. Li, M. Genov, A. Pretsch, D. Pretsch and M. G. Moloney, Tetramate Derivatives by Chemoselective Dieckmann Ring Closure of *threo* -Phenylserines and Their Antibacterial Activity, *J Org Chem*, 2022, **87**, 12240–12249.

10 K. Deng, Q.-Y. Huai, Z.-L. Shen, H.-J. Li, C. Liu and Y.-C. Wu, Rearrangement of Dypnones to 1,3,5-Triarylbenzenes, *Org Lett*, 2015, **17**, 1473–1476.

11 B.-H. Xing, X.-X. Zhao, Y.-J. Qin, P. Zhang and Z.-X. Guo, Ferric chloride–catalyzed deoxygenative chlorination of carbonyl compounds: A comparison of chlorodimethylsilane and dichloromethylsilane system, *J Chem Res*, 2020, **44**, 667–675.
